# Cancer‐associated stroma reveals prognostic biomarkers and novel insights into the tumour microenvironment of colorectal cancer and colorectal liver metastases

**DOI:** 10.1002/cam4.4452

**Published:** 2021-12-07

**Authors:** Kai M. Brown, Aiqun Xue, Ross C. Smith, Jaswinder S. Samra, Anthony J. Gill, Thomas J. Hugh

**Affiliations:** ^1^ Cancer Surgery and Metabolism Research Group Kolling Institute of Medical Research Royal North Shore Hospital St Leonards New South Wales Australia; ^2^ Upper GI Surgical Unit Royal North Shore Hospital and North Shore Private Hospital St Leonards New South Wales Australia; ^3^ Northern Clinical School Sydney Medical School University of Sydney Sydney New South Wales Australia; ^4^ Cancer Diagnosis and Pathology Group University of Sydney Kolling Institute of Medical Research Royal North Shore Hospital St Leonards New South Wales Australia

**Keywords:** biomarker, colorectal cancer, liver metastases, stroma, tumour microenvironment

## Abstract

**Background and Aims:**

Cancer‐associated stroma (CAS) is emerging as a key determinant of metastasis in colorectal cancer (CRC); however, little is known about CAS in colorectal liver metastases (CRLM). This study aimed to validate the prognostic significance of stromal protein biomarkers in primary CRC and CRLM. Secondly, this study aimed to describe the transcriptome of the CAS of CRLM and identify novel targetable pathways of metastasis.

**Methods:**

A case–control study design from a prospectively maintained database was adopted. The prognostic value of epithelial and stromal CALD1, IGFBP7, POSTN, FAP, TGF‐β and pSMAD2 expression was assessed by immunohistochemistry (IHC) in multivariate models. Pathway enrichment and sparse partial least square‐discriminant analysis (sPLS‐DA) were performed on a nested cohort after isolating epithelial tumour and CAS by laser capture microdissection.

**Results:**

110 CRCs with 124 paired CRLMs, and 110 matched non‐metastatic control CRCs were included. Median follow‐up was 62 and 45 months for primary and CRLM groups, respectively. Stromal FAP and POSTN were independent predictors for the development of CRLM. After CRLM resection, stromal IGFBP7 and POSTN were predictors of poorer survival. sPLS‐DA on the nested cohort identified a number of novel targetable stromal genes and pathways that defined poor prognosis CRC and the CAS of CRLM.

**Conclusions:**

This study is the first to describe key differences in stromal gene expression between paired primary CRC and CRLM as well as identifying several targetable biomarkers and transcriptomic pathways whose relevance specifically in the CAS of CRC and CRLM have not been previously described.

## Graphical abstract

Cancer‐associated stroma (CAS) is emerging as a key determinant of metastasis in colorectal cancer (CRC), however, little is known about CAS in colorectal liver metastases (CRLM). This study used a large cohort of paired primary CRC and CRLM in a case–control design to examine the transcriptome of the CAS. The key finding was that the only significant difference between patients who developed liver metastases versus those that had long‐term disease‐free survival was found in their CAS transcriptome. These data also identified a number of novel targetable stromal genes and pathways that defined poor prognosis CRC and the CAS of CRLM.

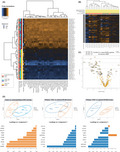

## 1 INTRODUCTION

Colorectal cancer (CRC) is the second leading cause of cancer death worldwide, most commonly from colorectal liver metastases (CRLM).^1^ Although a limited number of molecular characteristics such as *KRAS*, *BRAF* and microsatellite instability (MSI) status have established prognostic and predictive significance,^2^ these along with American Joint Committee on Cancer Staging (AJCC) criteria still fail to accurately identify which CRC patients will develop metastases, with up to 45% of intermediate stage cancer (Stages II and III) developing the metastatic disease within 5 years.^3^

There is a large body of emerging evidence pointing towards the most abundant component of the tumour microenvironment (TME), the cancer‐associated stroma (CAS), as a key factor in enabling invasion of primary CRC, and survival of cancer cells in the metastatic niche.^4^ The CMS4 molecular CRC subtype is characterised by abundant stromal infiltrate and stem‐like/mesenchymal transcriptomic features. CMS4 accounts for 25% of all CRC and harbours the poorest disease‐free (DFS) and overall survival (OS).^5^

Recent studies have shown that the defining transcriptional characteristics of the stem‐like/mesenchymal CMS4 subtype are conferred by the cancer‐associated fibroblasts (CAFs) rather than being intrinsic to the epithelial tumour cells.^6^ Indeed, complex CAF gene expression signatures have been associated with recurrence after primary CRC resection. These prognostic CAF signatures can be refined to a small subset of stromal proteins detectable by immunohistochemistry (IHC), including caldesmon‐1 (CALD1), insulin‐like growth factor binding protein 7 (IGFBP7), fibroblast activation protein (FAP) and periostin (POSTN).^7^ Furthermore, transforming growth factor‐beta (TGF‐β) signalling as identified by its intracellular response hallmark, pSMAD2, seemed to drive the expression of the prognostic CAF signature and was enriched in the poor prognosis CRC subtypes. TGF‐β’s central role in driving CRC metastasis and interaction with CAFs has been also described elsewhere.^8^, ^9^ Together these findings promote the CAS as a critical component of the tumour microenvironment for translational research. Focusing on the stromal TME is an attractive therapeutic strategy as it targets a genetically stable component of the tumour, theoretically less susceptible to treatment resistance, and may complement conventional and biological treatments.^10^ Currently, little is known about the role of CAS in CRLM or how the CAS differs between primary tumour and metastatic niches.

This study firstly aimed to validate the prognostic significance of proteins CALD1, IGFBP7, POSTN, FAP, TGF‐β (product encoded by the gene *TGFB1*) and pSMAD2 in primary CRC and similarly assess these biomarkers in CRLM by IHC with a view towards clinical application. Secondly, this study aimed to identify potential key stromal biomarkers or targetable pathways of metastasis by comparing the transcriptome of the epithelial tumour versus that of the CAS between paired primary CRCs and CRLMs supported with long‐term clinical data. This is, to the authors’ knowledge, the largest published data set of paired primary CRCs and CRLMs.

## 2 MATERIALS AND METHODS

### 2.1 Study design and patient selection

**FIGURE 1:**
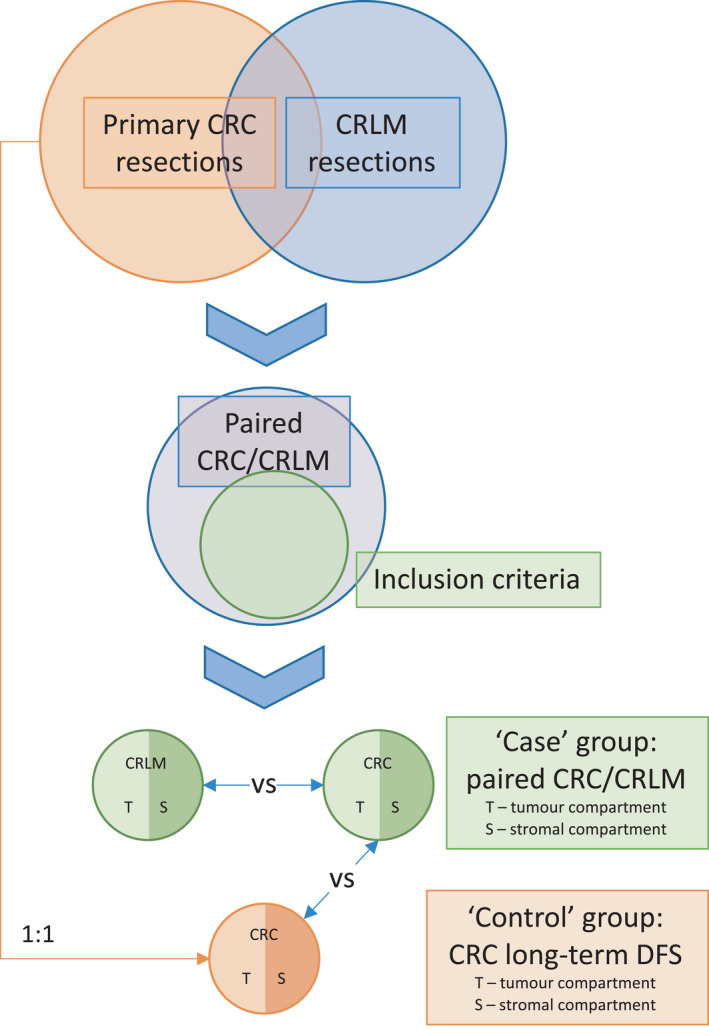
Study design showing the selection of case group of paired CRC/CRLM and matched control group of CRC With long‐term DFS, allowing two analyses to be performed. Each tumour had its epithelium tumour and stromal compartments assessed individually

A retrospective case–control study design was adopted by including all patients from January 1999 to December 2012 at Royal North Shore Hospital with either synchronous or metachronous liver‐only metastatic CRC from two previously described prospectively maintained databases of resected primary CRC^11^ and CRLM.^12^ Patients were excluded if their primary pathology tumour was anal in origin. An equal number of ‘control’ patients matched on age, sex, grade, T and N stage of their primary CRC (AJCC staging guidelines, 7th ed.^13^) who did not develop metastatic disease within at least 36 months were selected from the primary CRC database. Therefore, two analyses could be performed; case primary CRC versus control primary CRC and case primary CRC versus paired CRLM (Figure 1).

Ethics approval for the study was provided by the Human Research Ethics Committee of the Northern Sydney Local Health District, reference RESP/15/213.

### 2.2 Clinicopathological variables

The primary CRC data set variables included the patient age at resection, sex, year of resection, tumour location, histological subtype, use of neoadjuvant treatment stage, grade, pattern of growth, presence of peritumoural lymphocytic response, extramural venous permeation or discontinuous extramural tumour nodules, margin status, microsatellite instability (MSI) status and *BRAFV600E* mutation status.

The CRLM data set factors included age at resection, sex, year of resection, the temporal relationship of liver metastases as either synchronous (presenting within 4 months of the primary CRC diagnosis) or metachronous (identified at after 4 months from primary CRC diagnosis) and order of resection (liver or primary first or combined), type (as per the Brisbane 2000 terminology^14^) and complexity of liver resection,^15^ presence of primary CRC lymph node metastases, whether tumour marker carcinoembryonic antigen (CEA) was elevated at diagnosis, whether pre‐operative or post‐operative (relative to liver resection) chemotherapy was administered, the diameter of the largest resected tumour, the number of tumours, anatomical distribution of CRLM, CRLM tumour differentiation, MSI status, *BRAFV600E* and *KRAS* mutation status, estimated blood loss, the margin status and whether there were any post‐operative complications. R1 resection was defined as a microscopically positive margin. Major complications were defined as either Clavien–Dindo grade III or IV.^16^ Perioperative mortality referred to death during the same admission (in‐hospital) or within 90 days of surgery.

### 2.3 Tissue microarray and immunohistochemistry

Tissue microarrays (TMAs) of the primary tumours were utilised as previously described.^17^ TMAs of CRLMs were fashioned in a similar manner, in brief; inserting quadruplet 1 mm cores of formalin‐fixed, paraffin‐embedded (FFPE) tissue from a given specimen into a recipient paraffin block. Cores were sampled from distinct areas of the viable tumour confirmed on haematoxylin and eosin (H&E) stained whole sections to account for a degree of intra‐tumoural heterogeneity.

**FIGURE 2:**
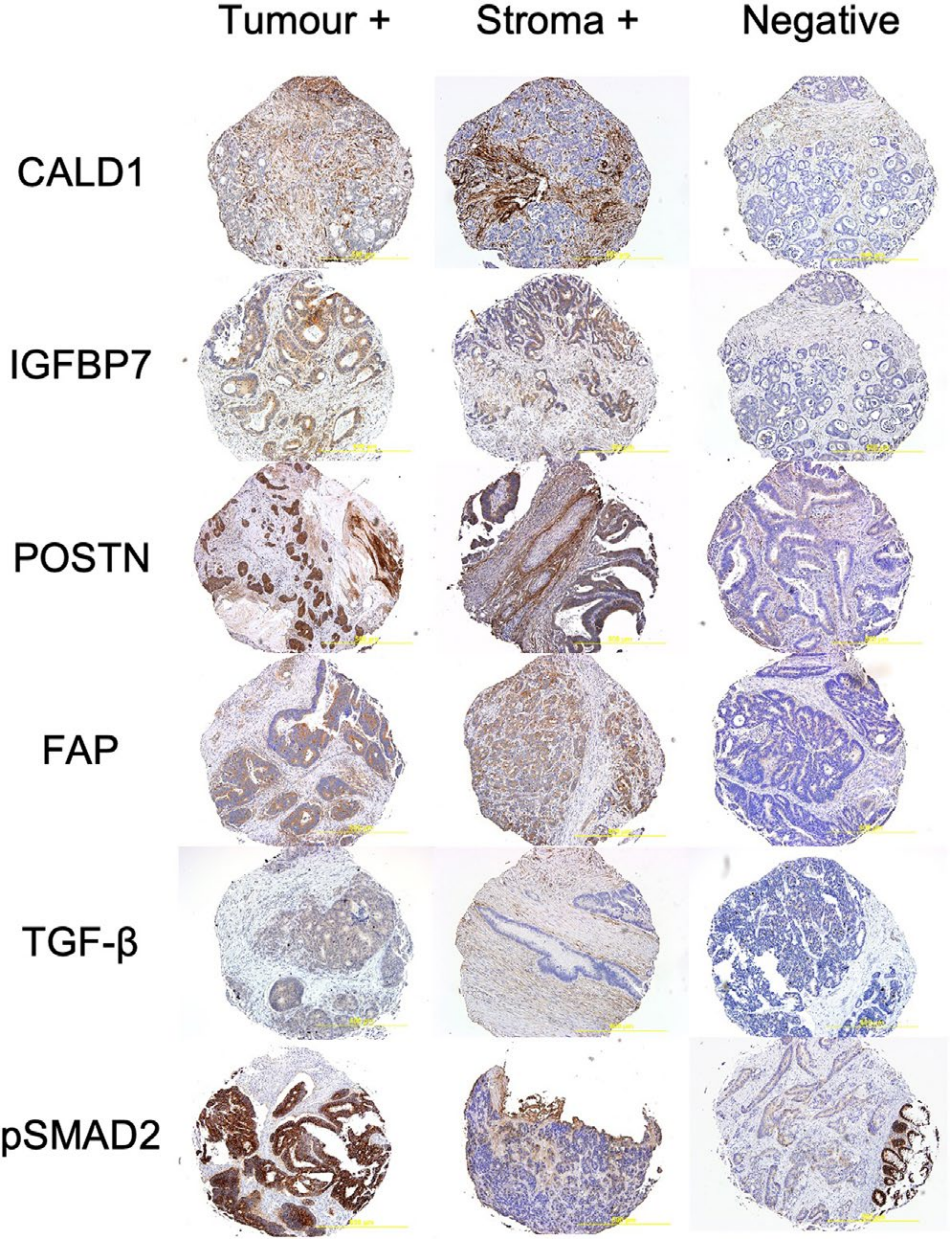
Representative TMA cores of CALD1, IGFBP7, POSTN, FAP, TGF‐β and pSMAD2 tumour and stroma positive samples. Negative shows no staining of CAS or epithelial tumour, ie background

Four micrometre thick sections were taken from each TMA block for IHC. Further details of IHC methodology are summarised in Supplementary Methods 1.1. Epithelial tumour and peritumoural stroma were scored separately over all available cores by two independent blinded assessors as either 0 for absent and 1, 2 or 3 for low, moderate and high intensity, respectively. Scores were allocated on the maximum intensity over most of the slides. The intensity was judged relative to the spectrum of staining across all slides after accounting for any non‐specific staining. Scores later underwent a binary transformation as described below in *Statistical Analysis* into positive and negative for clinical relevance (Figure 2). Missing cores, or cores where no viable cells could be identiﬁed, were excluded from the analysis. At least two viable cores were necessary for inclusion.

### 2.4 Laser capture microdissection, RNA extraction and gene expression analysis

Isolation of epithelial tumour and peritumoural stroma tissue compartments for gene expression analysis on a nested subset of the case–control cohort was achieved by laser capture microdissection (LCM) using an MMI CellCut Plus (MMI Molecular Machines & Industries). Specimens were selected such that they were chemotherapy naïve and had sufficient cell viability observed on H&E that they were likely to yield sufficient RNA for downstream analysis. Extensive measures were taken to optimise RNA yield and quality from archival FFPE specimens as well as ensure pure and specific tumour or stroma RNA, respectively.

After discarding the first 20, 10‐μm‐thick sections of FFPE tissue stained with MMI H&E Staining Kit Plus were mounted onto serial RNAse‐free PEN (polyethylene napthalate) MMI MembraneSlides and dissectates captured in UV cutting mode into MMI IsolationCaps as described previously.^18^ Tumour‐dense regions were easily identifiable based on H&E staining, and stromal regions were selected in the immediate peritumoural margin or in between nests of the tumour, as illustrated in Figure S1. Multiple sections manually dissected over several PEN slides were required per tissue compartment per tumour block to obtain sufficient RNA for analysis. Separate PEN slides and new microtome blades were used for either tumour or stroma within and between tumours, and pre‐LCM and post‐LCM scans of all slides were performed to avoid cross‐contamination. Fixation and staining of LCM slides were conducted under RNAse‐free conditions; all reagents including water were RNase‐free, PEN slides and CapSure^®^ caps were pre‐treated under UV light (max 245 nm) for 30 min and all other equipment and workspaces were treated with RNaseZap (Invitrogen).

After microdissection, dissectates were mixed with 150 μl of Buffer PKD and RNA extraction proceeded using RNeasy FFPE Kit (Qiagen). RNA quantification took place using the Qubit™ RNA HS Assay Kit (Invitrogen).

Total RNA isolates were prepared for nCounter analysis of a curated panel of 770 genes angiogenesis, extracellular matrix remodelling (ECM), epithelial‐to‐mesenchymal transition (EMT) and metastasis according to the manufacturer's instructions (NanoString Technologies). An annotated list of the curated nCounter^®^ PanCancer Progression gene set including housekeeping genes is listed in Table S1. The Nanostring nCounter platform was chosen to leverage the prospectively maintained clinical data of archival FFPE tissue whilst avoiding the issues of degraded RNA quality observed in other gene expression analysis modalities.^19^ Transcriptomic data are available from the corresponding author upon reasonable request.

### 2.5 Statistical analysis

Demographic descriptive statistics were reported median (IQR, interquartile range) values for continuous variables. Categorical variables were compared with the *χ* ^2^ test or Mcnemar's test and continuous variables were compared using a two‐sided Student's *t* test or Mann–Whitney U test, as appropriate.

In order to determine a clinically useful ‘positive’ cut‐off for each IHC biomarker and for downstream analysis, each underwent binary transformation using the ‘Evaluate Cutpoints’ application.^20^ For primary CRC, Youden Indices for the outcome of developing CRLM or not (i.e. belonging to case or control group) were used; and for CRLM, the maximal log‐rank statistic across possible cutpoints in a Cox proportional hazard (CPH) model for OS was used.

Prior to regression or survival analysis, missing data were imputed over 30 data sets using the chained random forest method^21^ over 100 trees and 10 iterations. All respective covariates were used in the random forest algorithm for each of the primary CRC and CRLM data sets, except for *KRAS* mutation status which was missing for 76% of CRLM cases.

A mixed effects logistic regression model was used to determine significant predictive factors for developing CRLM after primary CRC resection to account for the case–control matching. For CRLMs, Cox proportional hazards models were used to determine predictive factors for OS and DFS. OS was defined as the time from hepatic resection to the date of death (all‐cause mortality). DFS was defined as the time from liver surgery to the date of either death or first evidence of recurrence (intrahepatic or extrahepatic). Perioperative mortalities were excluded from survival analysis. Kaplan–Meier curves were constructed for OS and DFS, and univariable survival analysis was performed using the log‐rank test. For both models, potentially significant co‐variables identified in the univariable analysis (*p* < 0.250) were initially included before the purposeful selection of covariates method was used to select variables for the final multivariable models. Internal validation was performed via 10‐fold cross‐validation repeated 50 times (stratified for the random effect of case–control grouping) for the mixed effects model, and 1000 bootstrapped simulations for the survival models.

For gene expression analysis, raw count data were imported into nSolverTM (v3.0.22) and normalised using geometric mean scaling with a flagged minimum of 0.3 and a maximum of 3. Unsupervised hierarchical clustering was performed after *Z*‐score transformation of gene expression values and principal component analysis (PCA) after log2 transformation. Differential gene expression (DGE) was analysed using linear mixed modelling and then adjusted for multiple testing using Bonferroni correction. Finally, sparse partial least square‐discriminant analysis (sPLS‐DA) fitted onto two components with five‐fold cross‐validation was used as a supervised machine learning model to rank the most predictive features in the data that characterised the samples. Significant genes were considered those present in both the DGE analysis and the top 10 from the first sPLS‐DA component. Pathway and Gene Ontology enrichment was performed using BioCyc–HumanCyc database and web portal (Pathway Tools, version 21.0, BIOCYC13A, HumanCyc, version 21.1.).

All statistical analyses used R version 3.6.1 (http://www.r‐project.org) with a full list of packages summarised in Supplementary Methods 1.2. *p* value of <0.05 was considered significant.

## 3 RESULTS

### 3.1 Baseline characteristics

Baseline characteristics of the 110 cases with 124 paired CRLMs (13 cases had a second CRLM resected and one case a third) that met criteria, as well as their 110 matched controls, are shown in Table 1 and Table S2 respectively. Of note there was no difference in T/N stage or grade of the primary CRCs between groups, however, there was a significantly higher prevalence of other poor prognostic histological features amongst the case group. Median (IQR) follow‐up from resection in months was 66.32 (54.28, 87.43), 71.97 (37.85, 98.60) and 44.91 (24.85, 80.75) for case primary, control primary and CRLM groups, respectively.

**TABLE 1:** Primary CRC tumour baseline characteristics

	Control	Case	*p*
*N*	110	110	
Sex; male/female (%)	67/43 (60.9/39.1)	68/42 (61.8/38.2)	1.000
Age at resection (median [IQR])	63.43 [55.21, 71.58]	62.88 [56.26, 72.24]	0.694^^^
Year of resection (%)			
1998–2002	7 (6.4)	13 (11.8)	0.096
2003–2007	36 (32.7)	45 (40.9)	
2008–2012	67 (60.9)	52 (47.3)	
Follow‐up in months (median [IQR])	66.32 [54.28, 87.43]	71.97 [37.85, 98.60]	0.446^^^
Anatomical site of CRC (%)			
Caecum	18 (16.4)	15 (13.6)	0.763^#^
Ascending colon	13 (11.8)	10 (9.1)	
Hepatic flexure	0 (0.0)	1 (0.9)	
Transverse colon	5 (4.5)	8 (7.3)	
Splenic flexure	2 (1.8)	2 (1.8)	
Descending colon	7 (6.4)	3 (2.7)	
Sigmoid colon	26 (23.6)	31 (28.2)	
Rectum	39 (35.5)	40 (36.4)	
Anatomical side of CRC; Right/Left sided (%)	36/74 (32.7/67.3)	34/76 (30.9/69.1)	0.885
Histological subtype (%)			
Adenocarcinoma	106 (96.4)	101 (91.8)	0.620^#^
Mucinous	4 (3.6)	5 (4.5)	
Other	0 (0.0)	1 (0.9)	
NA	0 (0.0)	3 (2.7)	
Neoadjuvant treatment (%)	2 (1.8)	4 (3.6)	0.683^#^
Pathological T and N stage (%)			
I	9 (8.2)	10 (9.1)	0.984^#^
IIa	24 (21.8)	24 (21.8)	
IIb	7 (6.4)	6 (5.5)	
IIc	1 (0.9)	2 (1.8)	
IIIa	9 (8.2)	6 (5.5)	
IIIb	42 (38.2)	42 (38.2)	
IIIc	18 (16.4)	20 (18.2)	
Histological grade (%)			
Low	74 (67.3)	55 (50.0)	0.088
Mod	11 (10.0)	20 (18.2)	
High	16 (14.5)	13 (11.8)	
NA	9 (8.2)	22 (20.0)	
Pattern of growth (%)			
Circumscribed	3 (2.7)	2 (1.8)	0.051^#^
Infiltrative	41 (37.3)	44 (40.0)	
Irregular	0 (0.0)	2 (1.8)	
Pushing	44 (40.0)	23 (20.9)	
NA	22 (20.0)	39 (35.5)	
Peritumoural lymphocytic response (%)			
Absent	26 (23.6)	32 (29.1)	0.038
Present	62 (56.4)	36 (32.7)	
NA	22 (20.0)	42 (38.2)	
Thin‐walled vessel invasion (%)			
Absent	54 (49.1)	30 (27.3)	0.040
Present	32 (29.1)	37 (33.6)	
NA	24 (21.8)	43 (39.1)	
Extramural venous permeation (%)			
Absent	74 (67.3)	46 (41.8)	0.038
Present	15 (13.6)	22 (20.0)	
NA	21 (19.1)	42 (38.2)	
Discontinuous extramural tumour nodules (%)			
Absent	65 (59.1)	43 (39.1)	0.206
Present	24 (21.8)	26 (23.6)	
NA	21 (19.1)	41 (37.3)	
Margin status (%)			
R0	79 (71.8)	65 (59.1)	0.230^#^
R1	5 (4.5)	1 (0.9)	
NA	26 (23.6)	44 (40.0)	
MSI status (%)			
MSI‐L	84 (76.4)	87 (79.1)	0.105^#^
MSI‐H	11 (10.0)	4 (3.6)	
NA	15 (13.6)	19 (17.3)	
BRAF mutation status (%)			
Negative	75 (68.2)	84 (76.4)	0.052
Positive	17 (15.5)	7 (6.4)	
NA	18 (16.4)	19 (17.3)	

Note

Primary CRC baseline characteristics, stratified by case and control groups. All comparisons calculated based on complete cases using *X* ^2^ or otherwise ^ using Mann–Whitney U and # using fisher's exact test where appropriate.

### 3.2 Stromal biomarker IHC expression

Tumoural and stromal CALD1, IGFBP7, POSTN, FAP, TGF‐β and pSMAD2 positivity amongst case and control primary CRCs, and CRLMs are summarised in Table 2. Raw biomarker expression scores and binary transformation based on Youden Index and maximal log‐rank statistics, respectively, are available in Table S3A–C. There were several significant but weakly correlated biomarkers amongst both primary CRCs and CRLMs, as shown in Figure S2. Amongst case versus control primary CRC, there was a significantly higher proportion of POSTN tumour, POSTN stroma and FAP tumour positivity in the case group.

**TABLE 2:** Biomarker expression between case and control primary tumours and case primary tumours with their PAIRED CRLM

	Primaries		*p*	Paired CRLM	*p* ^$^
	Control	Case			
*N*	110	110		124	
CALD1 tumour (%)					
Negative	99 (90.0)	100 (90.9)	0.818	110 (88.7)	0.546
Positive	7 (6.4)	8 (7.3)		5 (4.0)	
Missing	4 (3.6)	2 (1.8)		9 (7.3)	
CALD1 stroma (%)					
Negative	84 (76.4)	86 (78.2)	0.945	4 (3.2)	**<0.001**
Positive	22 (20.0)	22 (20.0)		116 (93.5)	
Missing	4 (3.6)	2 (1.8)		4 (3.2)	
IGFBP7 tumour (%)					
Negative	28 (25.5)	17 (15.5)	0.075	18 (14.5)	0.689
Positive	78 (70.9)	87 (79.1)		97 (78.2)	
Missing	4 (3.6)	6 (5.5)		9 (7.3)	
IGFBP7 stroma (%)					
Negative	105 (95.5)	101 (91.8)	0.367^^^	114 (91.9)	1.000
Positive	1 (0.9)	3 (2.7)		4 (3.2)	
Missing	4 (3.6)	6 (5.5)		6 (4.8)	
POSTN tumour (%)					
Negative	99 (90.0)	89 (80.9)	**0.032^^^**	26 (21.0)	**<0.001**
Positive	5 (4.5)	14 (12.7)		90 (72.6)	
Missing	6 (5.5)	7 (6.4)		8 (6.5)	
POSTN stroma (%)					
Negative	25 (22.7)	11 (10.0)	**0.009**	109 (87.9)	**< 0.001**
Positive	79 (71.8)	94 (85.5)		8 (6.5)	
Missing	6 (5.5)	5 (4.5)		7 (5.6)	
FAP tumour (%)					
Negative	91 (82.7)	74 (67.3)	**0.01**	90 (72.6)	0.486
Positive	13 (11.8)	27 (24.5)		25 (20.2)	
Missing	6 (5.5)	9 (8.2)		9 (7.3)	
FAP stroma (%)					
Negative	86 (78.2)	74 (67.3)	0.131	105 (84.7)	**0.016**
Positive	18 (16.4)	26 (23.6)		10 (8.1)	
Missing	6 (5.5)	10 (9.1)		9 (7.3)	
TGF‐β tumour (%)					
Negative	15 (13.6)	14 (12.7)	0.777	95 (76.6)	**<0.001**
Positive	88 (80.0)	92 (83.6)		19 (15.3)	
Missing	7 (6.4)	4 (3.6)		10 (8.1)	
TGF‐β stroma (%)					
Negative	98 (89.1)	96 (87.3)	0.413	114 (91.9)	**0.016**
Positive	10 (9.1)	14 (12.7)		4 (3.2)	
Missing	2 (1.8)	0 (0.0)		6 (4.8)	
PSMAD2 tumour (%)					
Negative	88 (80.0)	85 (77.3)	0.706	93 (75.0)	1.000
Positive	20 (18.2)	22 (20.0)		24 (19.4)	
Missing	2 (1.8)	3 (2.7)		7 (5.6)	
PSMAD2 stroma (%)					
Negative	92 (83.6)	91 (82.7)	0.9	93 (75.0)	0.404
Positive	18 (16.4)	17 (15.5)		27 (21.8)	
Missing	0 (0.0)	2 (1.8)		4 (3.2)	

Note

All comparisons calculated on the basis of complete cases with ^ calculated using Fisher's test and $ comparing case primary tumours to matched first occurrence CRLM using Mcnemar's test. Significant values are highlighted in bold.

Overall biomarker expression pattern in the stromal compartment between paired primary CRC and CRLM shared no significant similarities (Cohen's Kappa −0.012, 95% CI −0.113 to 0.089, *p* = 0.769), however, tumoural expression was similar (Cohen's Kappa 0.209, 95% CI 0.127–0.291, *p* < 0.001). In terms of specific biomarkers, CALD1 stroma and POSTN tumour positivity was much greater amongst CRLM versus paired primary CRC (93.5% vs. 20%, *p* < 0.001 and 72.6% vs. 12.7%, *p* < 0.001, respectively), and POSTN stroma and TGF‐β tumour positivity was much less (6.5% vs. 85.5%, *p* < 0.001 and 15.3% vs. 83.6%, *p* < 0.001, respectively). TGF‐β stroma and FAP stroma positivity were moderately less amongst CRLM versus primary CRC (3.2% vs. 12.7%, *p* = 0.016 and 8.1% vs. 23.6%, *p* = 0.016, respectively). Across the whole panel, there was substantial (84.1%) persistence of biomarker expression pattern between paired first and second occurrence CRLMs (Cohen's Kappa 0.627, 95% CI 0.481–0.773, *p* < 0.001).

### 3.3 Prognostic significance of stromal biomarkers

**FIGURE 3:**
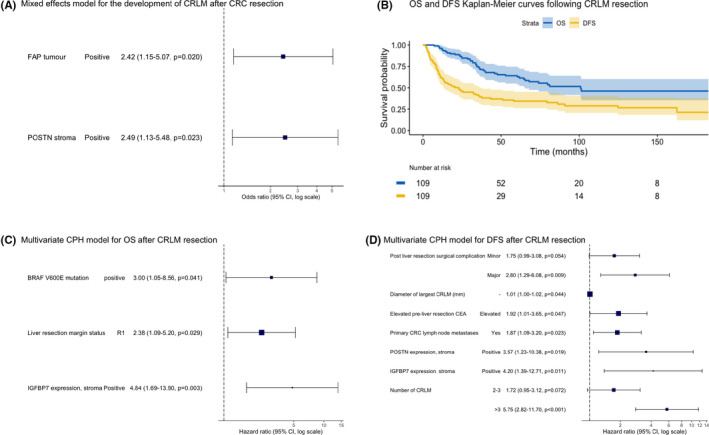
Prognostic stromal IHC biomarkers and survival modelling. (A) Odds ratio plot of independent predictors for the development of CRLM following primary CRC resection in a multivariate mixed effects model, (B) Kaplan–Meier curves for OS and DFS following CRLM resection, (C) Hazard ratio plot of independent predictors for OS after CRLM resection in a multivariate Cox proportional hazards model, (D) Hazard ratio plot of independent predictors for DFS after CRLM resection in a multivariate Cox proportional hazards model. 95% CI, 95% confidence interval

Full univariable and multivariable models for the development of CRLM after resection of primary CRC and for DFS and OS after resection of CRLM are shown in Tables S4 and S5, whereas hazard ratio plots of the final models are shown in Figure 3.

Amongst primary CRC, FAP stroma and POSTN stroma positivity were the only two independently predictive factors for the development of CRLM. There was no significant additive interaction between these biomarkers.

Following CRLM resection, median and 5‐year survivals were 101 months and 65%, and 23 months and 34%, for OS and DFS, respectively. Kaplan–Meier curves for OS and DFS are shown in Figure 3. *BRAFV600E* mutation, R1 margin status and IGFBP7 stroma positivity were independent predictors of poorer OS. Elevated pre‐operative CEA, larger CRLM, a greater number of CRLM, post‐operative complications, presence of primary lymph node metastases, IGFBP7 stroma positivity and POSTN stroma positivity were all independent predictors of poorer DFS.

### 3.4 Stromal gene expression analysis

A nested cohort of 10 case primary CRCs and corresponding CRLM as well as 10 matched control primary CRCs were technically suitable for DGE analysis after isolating tumour and stroma by LCM (Figure S1), yielding a total of 60 RNA samples. All patients in this nested cohort were chemotherapy naïve. There were no quality control flags in the normalisation process. Unsupervised hierarchical clustering and PCA on all 60 samples showed that the only discriminating factor between samples was their tissue compartment of origin (tumour or stroma) (Figure S3A,B).

Firstly, DGE analysis for the corresponding protein biomarkers measured by IHC was performed. Of these, none were differentially expressed comparing either tumour or stroma between case and control primary CRC nor between case primary CRC and paired CRLM (Figure S4).

**FIGURE 4:**
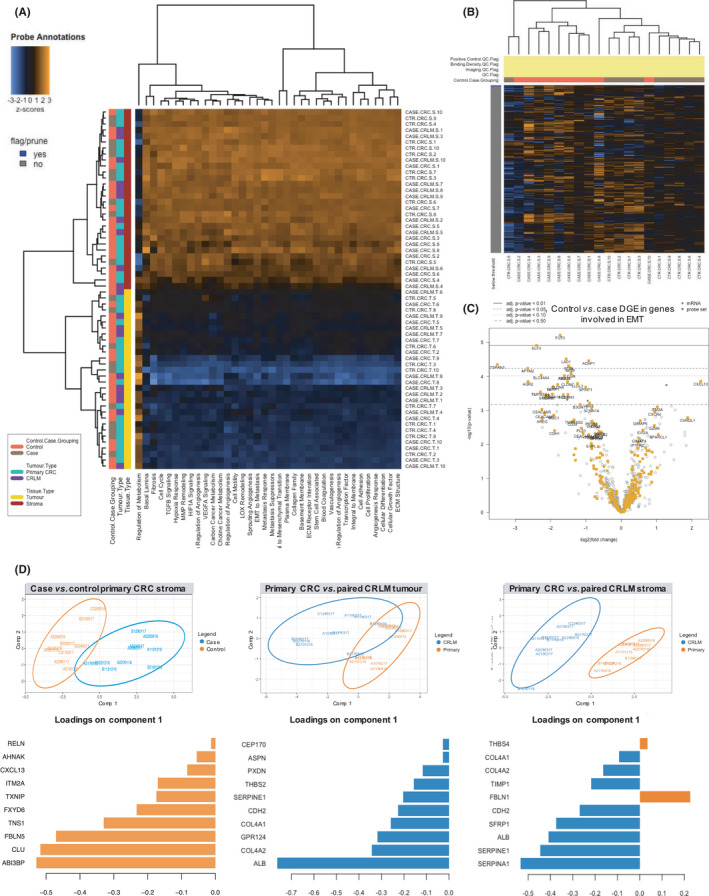
Stromal gene expression analysis. (A) DGE analysis heatmap with hierarchical clustering on key pathways present in the curated gene panel showing the major discriminating factor amongst samples was their tissue compartment (tumour or stroma), (B) DGE analysis heatmap with hierarchical clustering on genes involved in EMT amongst primary CRC stromal samples only showing a grouping tendency on the basis of case or control group, (C) Volcano plot of genes involved in EMT amongst primary CRC stromal samples only showing a general downregulation in the control group relative to cases, (D) sPLSDA analysis fitted onto two components with the topmost weighted genes accounting for the separation between groups and thus predicting their group membership

Next, DGE, pathway analysis and sPLS‐DA were performed for the entire 770 gene panel (Figure 4). A full list of DGE significant after Bonferroni correction and enriched pathways for all comparisons are contained in Tables S6 and S7. The only pattern on unsupervised hierarchical clustering of gene pathways (Figure 4A) revealed enrichment for genes involved in EMT in the stroma of case versus control primary CRCs (Figure 4B,C).

Notably, there was no DGE between case and control tumour compartments. Between case versus control primary CRC stroma, there were 16 differentially expressed genes, with *phospholipases* (LIPASYN‐PWY, *p* = 0.032) as the only enriched pathway. sPLS‐DA showed good separation between groups (classification error rate 13.5%), with 6 of the top 10 weighted genes in the first sPLS‐DA component (Figure 4D) also present in the DGE analysis (*ABI3BP*, *FBLN5*, *FXYD6*, *ITM2A*, *AHNAK* and *RELN*).

Only two genes were found to be differentially expressed between case primary and paired CRLM tumours; albumin (*ALB*) and collagen type IV alpha 2 chain (*COL4A2*). sPLS‐DA showed primary and CRLM tumour compartments to be similar (classification error rate 40.5%), with *ALB* overexpression in the CRLM tumour compartment responsible for most of the discrimination between groups (Figure 4D).

Finally, for case primary versus CRLM stroma, there were 726 differentially expressed genes, with 34 separate pathways enriched, the most statistically significant being the *BMP Signalling Pathway* (PWY66‐11, *p* = 8.4573465e‐8) and *MAP kinase cascade* (PWY66‐14, *p* = 1.915719e‐6). sPLS‐DA revealed very good discrimination between groups (classification error rate 5%). Nine of the top 10 weighted genes in the first sPLS‐DA component were also present in the DGE analysis (*SERPINA1*, *SERPINE1*, *ALB*, *SFRP1*, *CHD2*, *FBLN1*, *TIMP1*, *COL4A2* and *COL4A1*) (Figure 4D).

## 4 DISCUSSION

Overall, this study offers novel molecular insights into a central role for the CAS in CRC metastasis, both by identifying IHC biomarkers whose expression specifically in the stromal compartment were prognostic after both primary CRC and CRLM resection and by demonstrating that the defining transcriptomic features of primary CRC patients who develop CRLM also lay specifically in their stromal compartment. This study is the first to describe key differences in stromal gene expression between paired primary CRC and CRLM.

Multivariate mixed effect modelling identified two biomarkers whose expression specifically in the stromal compartment, FAP and POSTN, were prognostic for the development of CRLM after primary CRC resection. FAP is a widely accepted marker of CAFs—an activated functional subset of fibroblast demonstrated to interact with several epithelial tumours including colorectal, pancreatic and ovarian.^22^ CAFs in CRC have been shown to facilitate EMT and tumour invasion,^8^ promote tumour survival in the metastatic niche, affect treatment response^23^ and modulate immune response.^24^ POSTN is a cell adhesion protein overexpressed by CAFs in a number of cancers including CRC and has been suggested as a poor prognostic factor in a handful of studies.^25^, ^26^ A study using a murine breast cancer model found POSTN was necessary for cancer stem cell maintenance and survival in the metastatic niche by increasing Wnt signalling—a core pathway in CRC tumourigenesis.^27^ In vitro studies with human CRC cell lines have also suggested POSTN activates the PI3K/Akt pathway, blockade of which reversed the observed increase in proliferation, migration and chemoresistance conferred by POSTN overexpression.^28^ One study has even found serum levels of POSTN in patients correlated with primary CRC mRNA expression as well as the risk of distant metastasis.^29^

This study could not replicate all the findings of Calon et al., who defined prognostic CAF gene expression signatures and found that in addition to stromal FAP, stromal CALD1 and IGFBP7 were independently associated with poorer DFS after primary CRC resection.^7^ However, these conclusions were in a smaller retrospective cohort with an arbitrarily defined categorical cut‐off for the biomarkers, whereas the present study sought to define a clinically applicable ‘positive’ status using the robust statistical method described above.

The absence of any DGE in the case versus control primary epithelial tumour samples supports the case‐control matching and indicates that the study design had been selected appropriately to allow for the identification of prognostic stromal features. It is also noteworthy that there were so few changes in DGE between primary and paired CRLM tumours, and discrimination between groups in sPLS‐DA was predominantly due to albumin overexpression in the liver samples, possibly as a contaminant. This suggests that the fundamental change from primary to metastasis could be driven by gene expression changes elsewhere rather than within the tumour cells themselves.

Whilst genes involved in EMT appeared enriched in case primary CRC stroma on hierarchical clustering, only the phospholipases pathway was significantly enriched after DGE analysis. This is a high‐order pathway involved in a range of inflammatory,^30^ Wnt signalling^31^ and autophagy^32^ processes in CRC. Interestingly, aspirin, which is protective against the development^33^ and recurrence^34^ of CRC, directly and indirectly affects several targets in the phospholipase pathway. Research in this area has been predominantly limited to aspirin's effect on platelets and tumour cells, therefore further studies investigating the CAS as a therapeutic target of aspirin in CRC are warranted.

Interestingly, the top ten genes differentiating case versus control primary CRC stroma by sPLS‐DA were all over‐expressed in the control group. The two most highly weighted discriminant genes also present in the DGE analysis are *ABI3BP* and *FBLN5*, both of whose proteins are secreted into the extracellular matrix. *ABI3BP* has been reported to be downregulated in a number of cancers with supporting evidence that its expression promotes cellular senescence.^35^ Elsewhere, *ABI3BP* has been shown to regulate mesenchymal stem cell (MSC) biology with knockout mice increasing MSC in bone, liver and lung.^36^ MSC has been purported to differentiate into the functional CAF subset in the TME.^37^ *FBLN5* suppresses matrix metalloprotease 9, angiogenesis and epithelial cell motility in ovarian cancer,^38^ and blocks reactive oxygen species (ROS) in pancreatic cancer.^39^ *FBLN5* expression in CRC has been inversely correlated with the advanced AJCC stage.^40^ A decrease in *FBLN5* expression in CRC relative to normal tissue has been observed in one other study, which also showed *FBLN5* enhanced apoptosis via the ROS/MAP kinase (MAPK) and Akt signal pathways in eight CRC cell lines.^41^ The present study's novel observation that these classically considered ‘tumour suppressors’ prognostic relevance is derived from the CAS likely represents the product of some paracrine manipulation by the epithelial tumour and a potential target for the therapeutic blockade.

There was no significant maintenance of IHC biomarker expression specifically in the stromal compartment between paired primary CRCs and CRLMs. It is unclear from this study whether this represents greater intrinsic variability in the stromal transcriptome between primary and metastatic lesions or reflects their differing tissues of origin. Nonetheless, this corresponds closely with the observation elsewhere of 60% overall concordance in CMS gene expression subtype seen between primary and metastatic CRC tumours, but only 47% for the CMS4 mesenchymal subtype,^42^ which is defined by its stromal transcriptome.^6^, ^7^ It should also be noted that significant effort was made to only extract the peritumoural stroma from both primary CRC and CRLM, which also shared the same histological appearance across anatomical locations.

This study identified stromal IGFBP7 expression by IHC as being prognostic for poorer OS, and stromal IGFBP7 and POSTN as prognostic for poorer DFS after CRLM resection. Of note, POSTN was also prognostic following primary CRC resection. IGFBP7 (also known as IGFBP‐rP1 or MAC25) is a secreted growth regulatory and adhesion protein. The literature surrounding IGFBP7 and CRC is conflicting. Early work on epithelial CRC cell lines found IGFBP7 expression is associated with tumour suppression.^43^ More recently, IGFBP7 knockdown in a CRC cell line was found to induce an EMT phenotype, and IGFBP7 overexpression resulted in significantly smaller tumours with fewer lung metastases in a murine model.^44^ Others have similarly found IGFBP7 to inhibit EMT.^45^ In stark contrast, however, IGFBP7 expression has also been associated with an increase in liver metastasis in rat models.^46^ Specifically, *stromal* IGFBP7 has been shown to be co‐regulated by Wnt and TGF‐β signalling^47^ and additionally detected in malignant epithelial CRC cells with a mesenchymal phenotype having undergone EMT.^48^ In the same study, stromal IGBP7 was also shown to promote CRC colony formation in vitro. It is possible that IGFBP7 in CRC has functionally opposite roles in the stromal versus epithelial compartment of the tumour, explaining its prognostic significance specifically in the stroma in the present study.

This study has also described the transcriptome of the stromal microenvironment of CRLM. The greatest number of DGE were between the paired primary CRC and CRLM stroma, again possibly due to the greater intrinsic variability of CAS or due to their different tissue of origin. sPLS‐DA revealed that the topmost discriminating genes were the overexpression of two serine protease inhibitors (*SERPINA1 and SERPINE1*), *ALB* and *SFRP1* in the CRLM stroma. Again, albumin may also reflect the tissue of origin. Overexpression of both *SERPINA1* and *SERPINE1* has been associated with a poorer prognosis in primary CRC,^49^ whereas the SFRP family of secreted proteins are counterintuitively considered tumour suppressors. SFRP normally bind to Wnt ligands to suppress Wnt signalling.^50^ One study found silencing of *SFRP1* via hypermethylation of itself or its promoter regions are associated with CRC carcinogenesis.^51^ Another recent study similarly observed higher methylation levels of *SFRP1* in primary CRC tissue compared to the normal colon, however, it also found *SFRP1* hypermethylation to be a favourable prognostic factor in multivariate analysis.^52^ The aforementioned studies were all conducted on whole mixed tumour samples, so it may be that the overexpression of *SFRP1* in the CRLM stroma in sPLS‐DA reflects a different functional significance in the stromal versus epithelial compartments or similarly over advancing cancer stage.

Pathway analysis revealed both the BMP signalling pathway and the MAPK cascade were enriched amongst the CRLM versus primary CRC stromal compartment. The role of these pathways in driving carcinogenesis in epithelial CRC cells is well established,^53^ however, their significance specifically within the stromal compartment of CRC is yet to be fully elucidated. BMP signalling has been associated with mesenchymal‐type poor prognosis CRC, where it induces an EMT phenotype via interactions with the NOTCH pathway,^54^ which in turn drives metastasis.^55^ MAPK activation in intestinal mesenchymal cells has been found to promote tumourigenesis in a murine CRC model.^56^ It is possible that activation of the MAP kinase cascade in the stroma may be due to interactions with the s‘malignant phenotype’ extracellular matrix, which has been observed in breast cancer in vitro.^57^

An alternate explanation is that the enrichment of BMP and MAPK pathways in the stromal compartment reflects a broader TGF‐β activation of the CAS, as both are targets of TGF‐β.^58^ Although this study observed significantly lower TGF‐β expression by IHC in both CRLM tumour and stroma versus paired primary cancers, evidence of TGF‐β signalling via pSMAD2 expression was similar. Stromal TGF‐β also correlated positively with stromal IGFBP7 expression in CRLMs. TGF‐β has been shown in a number of in vitro and in vivo studies to be a key mediator activating CAFs and enhancing tumour survival in the metastatic niche.^8^ Notably, several drugs including RAF and MEK inhibitors (for MAPK),^59^ TGF‐β inhibitors (for MAPK and BMP)^60^, ^61^ and direct BMP antagonists^62^ that target these pathways currently exist or are in development. Further studies are warranted to investigate the role of these drugs in potentially rendering the metastatic niche inhospitable specifically by targeting the CAS.

Only one other study has attempted to examine CAFs in CRLM. Berdiel‐Acer et al. assessed change in gene expression from normal colonic fibroblasts (NCF), to CAFs from primary CRC to (unpaired) CAFs from CRLM.^63^ CAFs were isolated into a culture medium by homogenising fresh specimens before depleting epithelial cells. The study defined stromal gene signatures as strongly prognostic for DFS following primary CRC resection independent of the AJCC stage, supporting the role of CAS in CRC progression. Whilst the authors acknowledge their methodology derived a prognostic signature defined by the transition from NCF to CAF and thus there could exist other prognostic CAF‐specific genes, a number of the highest weighted genes in their signatures were also significant in the present study, including *POSTN*, *SERPINE1*, *CDH2* and *FBLN1*.

Limitations of this study include missing data regarding *KRAS* and *BRAF* status. It was also incongruent that the prognostic markers identified by IHC were not significantly different between case and control groups in the DGE analysis. This was most likely due to the low total number of patients in this nested cohort. However, this study's results are supported by a large number of paired primary and metastatic tumour samples with a well‐matched control group, the high transcriptomic resolution of isolated stromal and epithelial tumour compartments and the long clinical follow‐up time.

In conclusion, the broad observation from these data was that the main biological difference between case and control groups lay not in the tumour itself but in how the host (stroma) responds to the tumour. This study has confirmed the independent prognostic significance of stromal IHC biomarkers FAP and POSTN in primary CRC, and IGFBP7 and POSTN in CRLM. Although these prognostic markers need to be validated in an external cohort, these findings do suggest that in the same manner that tumour–stroma interactions in the primary tumour in part determine their potential for recurrence, so do they in the metastatic niche. Finally, this study has also described transcriptomic characteristics specific to the stromal compartment of poor‐prognosis primary CRC as well as CRLM, offering several potential novel therapeutic targets for further investigation.

## CONFLICT OF INTEREST

The authors declare no potential conflicts of interest.

## AUTHOR CONTRIBUTION

Kai M. Brown was involved in the study conception, design, performance of experiments, collection of data, analysis and manuscript writing. Aiqun Xue was involved in the study design and performance of experiments. Ross C. Smith was involved in the study conception and design. Jaswinder S. Samra was involved in the study conception and collection of data. Anthony J. Gill was involved in the study conception, design and analysis. Thomas J. Hugh was involved in the study conception, design, collection of data, analysis and manuscript writing.

## ETHICS STATEMENT

Ethics approval for the study was provided by the Human Research Ethics Committee of the Northern Sydney Local Health District (reference RESP/15/213).

## Supporting information

Fig S1‐S3

Table S1

Table S2‐S7

Method S1

## Data Availability

Original data may be made available upon request to the corresponding author.
