# Supplementary material for: Cancer‐associated stroma reveals prognostic biomarkers and novel insights into the tumour microenvironment of colorectal cancer and colorectal liver metastases
Source: Cancer Med. 2021 Dec 7;11(2):492–506. doi: 10.1002/cam4.4452 (PMC8729056; doi:10.1002/cam4.4452)
Supplement: Supplementary file 1 — Fig S1‐S3 [file CAM4-11-492-s004.docx]

**Supplementary Figures**

**
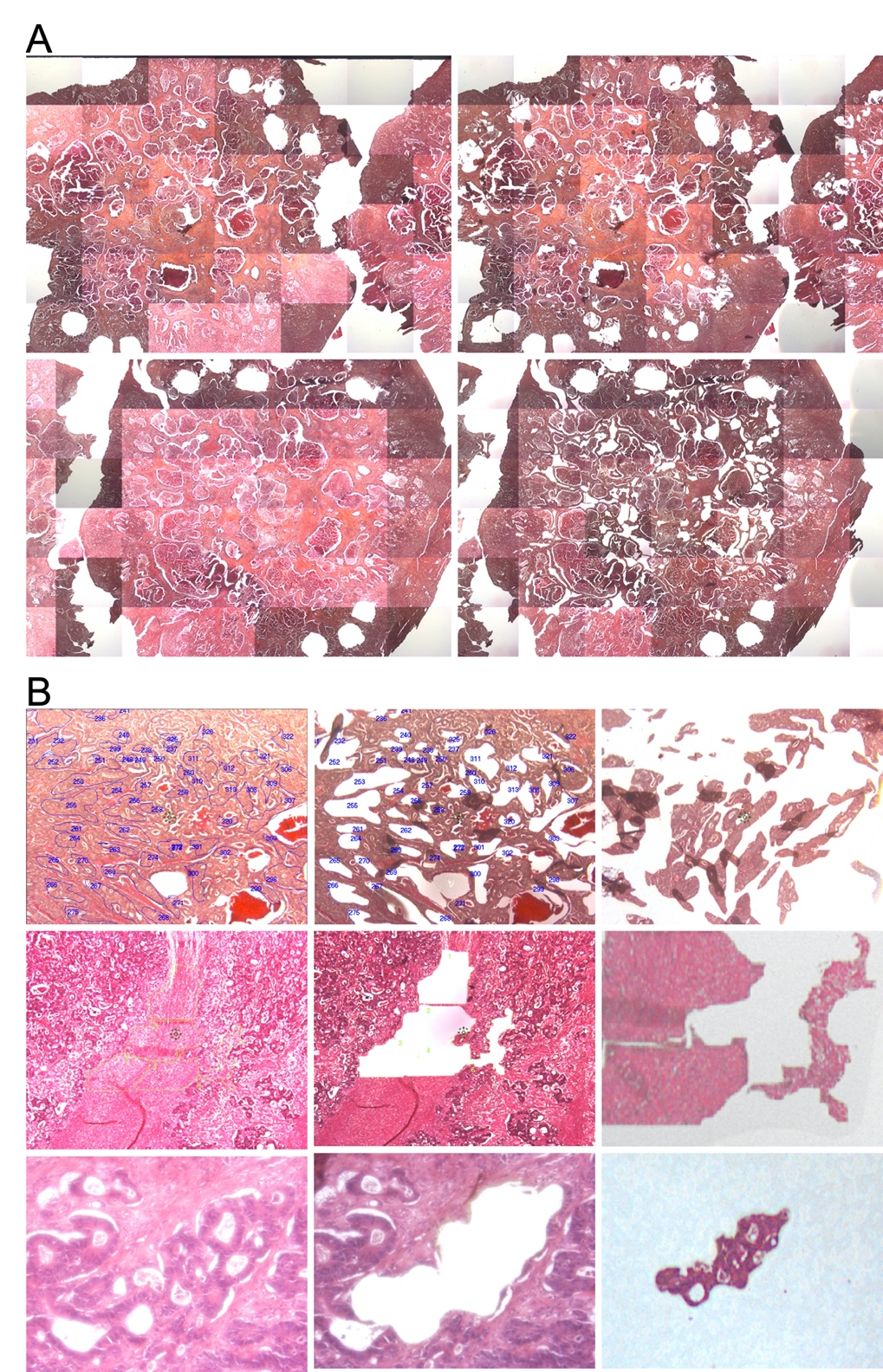
**

**Supplementary Figure 1 -** Representative LCM dissections of epithelial tumour and peri-tumoral stroma. (A) (composite of whole slide scanning at 4x magnification); top row shows pre- and post-dissection of tumour, bottom row shows pre- and post-dissection of stroma. 1mm tissue cores from TMA fabrication are visible. (B) consecutive columns show pre- and post-dissection and dissectate, respectfully. First row shows epithelial tumour dissection at 4x magnification. Second row shows stromal dissection at 4x magnification. Third row shows tumour dissection at 20x magnification.

**Supplementary Figure 2 -** DGE analysis on all samples with (A) hierarchical clustering on all genes and (B) PCA on two components highlighting cases vs. controls and tumour vs. stroma showing that the only discriminating factor amongst samples was their tissue compartment (tumour or stroma).

**Supplementary Figure 3 -** DGE analysis of genes represented in IHC stromal biomarkers panel showing no significant difference in expression between case and control primary CRC nor case primary CRC and CRLM, for either tumour or stromal compartments.
