# Supplementary material for: Cancer‐associated stroma reveals prognostic biomarkers and novel insights into the tumour microenvironment of colorectal cancer and colorectal liver metastases
Source: Cancer Med. 2021 Dec 7;11(2):492–506. doi: 10.1002/cam4.4452 (PMC8729056; doi:10.1002/cam4.4452)
Supplement: Supplementary file 2 — Table S1 [file CAM4-11-492-s002.docx]

**Supplementary Tables**

**Supplementary Table 1** – Full list of genes included in the the nCounter® PanCancer Progression gene set.

| **Official Symbol** | **Accession** | **Alias / Prev Symbol** | **Official Full Name** |
| --- | --- | --- | --- |
| AAMP | [NM_001087.3](http://www.ncbi.nlm.nih.gov/entrez/viewer.fcgi?db=nucleotide&val=NM_001087.3) | - | angio-associated, migratory cell protein |
| ABI3BP | [NM_015429.3](http://www.ncbi.nlm.nih.gov/entrez/viewer.fcgi?db=nucleotide&val=NM_015429.3) | NESHBP\|TARSH | ABI family, member 3 (NESH) binding protein |
| ACHE | [NM_000665.3](http://www.ncbi.nlm.nih.gov/entrez/viewer.fcgi?db=nucleotide&val=NM_000665.3) | ACEE\|ARACHE\|N-ACHE\|YT | acetylcholinesterase |
| ACTG2 | [NM_001615.3](http://www.ncbi.nlm.nih.gov/entrez/viewer.fcgi?db=nucleotide&val=NM_001615.3) | ACT\|ACTA3\|ACTE\|ACTL3\|ACTSG | actin, gamma 2, smooth muscle, enteric |
| ACVR1 | [NM_001105.2](http://www.ncbi.nlm.nih.gov/entrez/viewer.fcgi?db=nucleotide&val=NM_001105.2) | ACTRI\|ACVR1A\|ACVRLK2\|ALK2\|FOP\|SKR1\|TSRI | activin A receptor, type I |
| ACVR1C | [NM_145259.2](http://www.ncbi.nlm.nih.gov/entrez/viewer.fcgi?db=nucleotide&val=NM_145259.2) | ACVRLK7\|ALK7 | activin A receptor, type IC |
| ACVRL1 | [NM_000020.1](http://www.ncbi.nlm.nih.gov/entrez/viewer.fcgi?db=nucleotide&val=NM_000020.1) | ACVRLK1\|ALK-1\|ALK1\|HHT\|HHT2\|ORW2\|SKR3\|TSR-I | activin A receptor type II-like 1 |
| ADAM15 | [NM_207195.1](http://www.ncbi.nlm.nih.gov/entrez/viewer.fcgi?db=nucleotide&val=NM_207195.1) | MDC15 | ADAM metallopeptidase domain 15 |
| ADAM17 | [NM_003183.4](http://www.ncbi.nlm.nih.gov/entrez/viewer.fcgi?db=nucleotide&val=NM_003183.4) | ADAM18\|CD156B\|CSVP\|NISBD\|TACE | ADAM metallopeptidase domain 17 |
| ADAM28 | [NM_014265.4](http://www.ncbi.nlm.nih.gov/entrez/viewer.fcgi?db=nucleotide&val=NM_014265.4) | ADAM 28\|ADAM23\|MDC-L\|MDC-Lm\|MDC-Ls\|MDCL\|eMDC II\|eMDCII | ADAM metallopeptidase domain 28 |
| ADAM8 | [NM_001109.4](http://www.ncbi.nlm.nih.gov/entrez/viewer.fcgi?db=nucleotide&val=NM_001109.4) | CD156\|MS2 | ADAM metallopeptidase domain 8 |
| ADAM9 | [NM_001005845.1](http://www.ncbi.nlm.nih.gov/entrez/viewer.fcgi?db=nucleotide&val=NM_001005845.1) | CORD9\|MCMP\|MDC9\|Mltng | ADAM metallopeptidase domain 9 |
| ADAMTS1 | [NM_006988.3](http://www.ncbi.nlm.nih.gov/entrez/viewer.fcgi?db=nucleotide&val=NM_006988.3) | C3-C5\|METH1 | ADAM metallopeptidase with thrombospondin type 1 motif, 1 |
| ADAMTS12 | [NM_030955.2](http://www.ncbi.nlm.nih.gov/entrez/viewer.fcgi?db=nucleotide&val=NM_030955.2) | PRO4389 | ADAM metallopeptidase with thrombospondin type 1 motif, 12 |
| ADAMTS8 | [NM_007037.4](http://www.ncbi.nlm.nih.gov/entrez/viewer.fcgi?db=nucleotide&val=NM_007037.4) | ADAM-TS8\|METH2 | ADAM metallopeptidase with thrombospondin type 1 motif, 8 |
| ADAP1 | [NM_006869.2](http://www.ncbi.nlm.nih.gov/entrez/viewer.fcgi?db=nucleotide&val=NM_006869.2) | CENTA1\|GCS1L\|p42IP4 | ArfGAP with dual PH domains 1 |
| ADD1 | [NM_001119.4](http://www.ncbi.nlm.nih.gov/entrez/viewer.fcgi?db=nucleotide&val=NM_001119.4) | ADDA | adducin 1 (alpha) |
| ADM2 | [NM_001253845.1](http://www.ncbi.nlm.nih.gov/entrez/viewer.fcgi?db=nucleotide&val=NM_001253845.1) | AM2\|dJ579N16.4 | adrenomedullin 2 |
| ADRA2B | [NM_000682.4](http://www.ncbi.nlm.nih.gov/entrez/viewer.fcgi?db=nucleotide&val=NM_000682.4) | ADRA2L1\|ADRA2RL1\|ADRARL1\|ALPHA2BAR\|alpha-2BAR | adrenoceptor alpha 2B |
| AEBP1 | [NM_001129.3](http://www.ncbi.nlm.nih.gov/entrez/viewer.fcgi?db=nucleotide&val=NM_001129.3) | ACLP | AE binding protein 1 |
| AGGF1 | [NM_018046.3](http://www.ncbi.nlm.nih.gov/entrez/viewer.fcgi?db=nucleotide&val=NM_018046.3) | GPATC7\|GPATCH7\|HSU84971\|HUS84971\|VG5Q | angiogenic factor with G patch and FHA domains 1 |
| AGR2 | [NM_006408.3](http://www.ncbi.nlm.nih.gov/entrez/viewer.fcgi?db=nucleotide&val=NM_006408.3) | AG2\|GOB-4\|HAG-2\|PDIA17\|XAG-2 | anterior gradient 2 homolog (Xenopus laevis) |
| AGRN | [NM_198576.2](http://www.ncbi.nlm.nih.gov/entrez/viewer.fcgi?db=nucleotide&val=NM_198576.2) | - | agrin |
| AGT | [NM_000029.3](http://www.ncbi.nlm.nih.gov/entrez/viewer.fcgi?db=nucleotide&val=NM_000029.3) | ANHU\|SERPINA8 | angiotensinogen (serpin peptidase inhibitor, clade A, member 8) |
| AHNAK | [NM_001620.2](http://www.ncbi.nlm.nih.gov/entrez/viewer.fcgi?db=nucleotide&val=NM_001620.2) | AHNAKRS | AHNAK nucleoprotein |
| AKAP12 | [NM_005100.3](http://www.ncbi.nlm.nih.gov/entrez/viewer.fcgi?db=nucleotide&val=NM_005100.3) | AKAP250\|SSeCKS | A kinase (PRKA) anchor protein 12 |
| AKAP2 | [NM_001004065.4](http://www.ncbi.nlm.nih.gov/entrez/viewer.fcgi?db=nucleotide&val=NM_001004065.4) | AKAP-2\|AKAPKL\|PRKA2 | A kinase (PRKA) anchor protein 2 |
| AKT1 | [NM_005163.2](http://www.ncbi.nlm.nih.gov/entrez/viewer.fcgi?db=nucleotide&val=NM_005163.2) | AKT\|PKB\|PKB-ALPHA\|PRKBA\|RAC\|RAC-ALPHA | v-akt murine thymoma viral oncogene homolog 1 |
| AKT2 | [NM_001626.2](http://www.ncbi.nlm.nih.gov/entrez/viewer.fcgi?db=nucleotide&val=NM_001626.2) | HIHGHH\|PKBB\|PKBBETA\|PRKBB\|RAC-BETA | v-akt murine thymoma viral oncogene homolog 2 |
| AKT3 | [NM_005465.4](http://www.ncbi.nlm.nih.gov/entrez/viewer.fcgi?db=nucleotide&val=NM_005465.4) | PKB-GAMMA\|PKBG\|PRKBG\|RAC-PK-gamma\|RAC-gamma\|STK-2 | v-akt murine thymoma viral oncogene homolog 3 (protein kinase B, gamma) |
| ALB | [NM_000477.5](http://www.ncbi.nlm.nih.gov/entrez/viewer.fcgi?db=nucleotide&val=NM_000477.5) | PRO0883\|PRO0903\|PRO1341 | albumin |
| ALDOA | [NM_184041.2](http://www.ncbi.nlm.nih.gov/entrez/viewer.fcgi?db=nucleotide&val=NM_184041.2) | ALDA\|GSD12 | aldolase A, fructose-bisphosphate |
| ALOX5 | [NM_000698.2](http://www.ncbi.nlm.nih.gov/entrez/viewer.fcgi?db=nucleotide&val=NM_000698.2) | 5-LO\|5-LOX\|5LPG\|LOG5 | arachidonate 5-lipoxygenase |
| AMH | [NM_000479.3](http://www.ncbi.nlm.nih.gov/entrez/viewer.fcgi?db=nucleotide&val=NM_000479.3) | MIF\|MIS | anti-Mullerian hormone |
| ANG | [NM_001145.4](http://www.ncbi.nlm.nih.gov/entrez/viewer.fcgi?db=nucleotide&val=NM_001145.4) | ALS9\|HEL168\|RNASE4\|RNASE5 | angiogenin, ribonuclease, RNase A family, 5 |
| ANGPT1 | [NM_001146.3](http://www.ncbi.nlm.nih.gov/entrez/viewer.fcgi?db=nucleotide&val=NM_001146.3) | AGP1\|AGPT\|ANG1 | angiopoietin 1 |
| ANGPT2 | [NM_001147.2](http://www.ncbi.nlm.nih.gov/entrez/viewer.fcgi?db=nucleotide&val=NM_001147.2) | AGPT2\|ANG2 | angiopoietin 2 |
| ANGPTL2 | [NM_012098.2](http://www.ncbi.nlm.nih.gov/entrez/viewer.fcgi?db=nucleotide&val=NM_012098.2) | ARP2\|HARP | angiopoietin-like 2 |
| ANGPTL4 | [NR_104213.1](http://www.ncbi.nlm.nih.gov/entrez/viewer.fcgi?db=nucleotide&val=NR_104213.1) | ANGPTL2\|ARP4\|FIAF\|HFARP\|NL2\|PGAR\|pp1158 | angiopoietin-like 4 |
| ANPEP | [NM_001150.1](http://www.ncbi.nlm.nih.gov/entrez/viewer.fcgi?db=nucleotide&val=NM_001150.1) | APN\|CD13\|GP150\|LAP1\|P150\|PEPN | alanyl (membrane) aminopeptidase |
| ANXA2 | [NR_003573.1](http://www.ncbi.nlm.nih.gov/entrez/viewer.fcgi?db=nucleotide&val=NR_003573.1) | ANX2\|ANX2L4\|CAL1H\|LIP2\|LPC2\|LPC2D\|P36\|PAP-IV | annexin A2 |
| AP1M2 | [NM_005498.4](http://www.ncbi.nlm.nih.gov/entrez/viewer.fcgi?db=nucleotide&val=NM_005498.4) | AP1-mu2\|HSMU1B\|MU-1B\|MU1B\|mu2 | adaptor-related protein complex 1, mu 2 subunit |
| APC | [NM_000038.3](http://www.ncbi.nlm.nih.gov/entrez/viewer.fcgi?db=nucleotide&val=NM_000038.3) | BTPS2\|DP2\|DP2.5\|DP3\|GS\|PPP1R46 | adenomatous polyposis coli |
| APOD | [NM_001647.3](http://www.ncbi.nlm.nih.gov/entrez/viewer.fcgi?db=nucleotide&val=NM_001647.3) | - | apolipoprotein D |
| APOE | [NM_000041.2](http://www.ncbi.nlm.nih.gov/entrez/viewer.fcgi?db=nucleotide&val=NM_000041.2) | AD2\|LDLCQ5\|LPG | apolipoprotein E |
| APOH | [NM_000042.2](http://www.ncbi.nlm.nih.gov/entrez/viewer.fcgi?db=nucleotide&val=NM_000042.2) | B2G1\|B2GP1\|BG | apolipoprotein H (beta-2-glycoprotein I) |
| AQP1 | [NM_198098.1](http://www.ncbi.nlm.nih.gov/entrez/viewer.fcgi?db=nucleotide&val=NM_198098.1) | AQP-CHIP\|CHIP28\|CO | aquaporin 1 (Colton blood group) |
| ARAP2 | [NM_015230.2](http://www.ncbi.nlm.nih.gov/entrez/viewer.fcgi?db=nucleotide&val=NM_015230.2) | CENTD1\|PARX | ArfGAP with RhoGAP domain, ankyrin repeat and PH domain 2 |
| AREG | [NM_001657.2](http://www.ncbi.nlm.nih.gov/entrez/viewer.fcgi?db=nucleotide&val=NM_001657.2) | AR\|CRDGF\|SDGF | amphiregulin |
| ARHGAP32 | [NM_001142685.1](http://www.ncbi.nlm.nih.gov/entrez/viewer.fcgi?db=nucleotide&val=NM_001142685.1) | GC-GAP\|GRIT\|PX-RICS\|RICS\|p200RhoGAP\|p250GAP | Rho GTPase activating protein 32 |
| ARHGDIB | [NM_001175.4](http://www.ncbi.nlm.nih.gov/entrez/viewer.fcgi?db=nucleotide&val=NM_001175.4) | D4\|GDIA2\|GDID4\|LYGDI\|Ly-GDI\|RAP1GN1\|RhoGDI2 | Rho GDP dissociation inhibitor (GDI) beta |
| ASPN | [NM_017680.3](http://www.ncbi.nlm.nih.gov/entrez/viewer.fcgi?db=nucleotide&val=NM_017680.3) | OS3\|PLAP-1\|PLAP1\|SLRR1C | asporin |
| ATPIF1 | [NM_178190.2](http://www.ncbi.nlm.nih.gov/entrez/viewer.fcgi?db=nucleotide&val=NM_178190.2) | ATPI\|ATPIP\|IP | ATPase inhibitory factor 1 |
| B3GNT3 | [NM_014256.3](http://www.ncbi.nlm.nih.gov/entrez/viewer.fcgi?db=nucleotide&val=NM_014256.3) | B3GAL-T8\|B3GN-T3\|B3GNT-3\|HP10328\|TMEM3\|beta3Gn-T3 | UDP-GlcNAc:betaGal beta-1,3-N-acetylglucosaminyltransferase 3 |
| BAD | [NM_004322.3](http://www.ncbi.nlm.nih.gov/entrez/viewer.fcgi?db=nucleotide&val=NM_004322.3) | BBC2\|BCL2L8 | BCL2-associated agonist of cell death |
| BAG2 | [NM_004282.3](http://www.ncbi.nlm.nih.gov/entrez/viewer.fcgi?db=nucleotide&val=NM_004282.3) | BAG-2\|dJ417I1.2 | BCL2-associated athanogene 2 |
| BAI1 | [NM_001702.1](http://www.ncbi.nlm.nih.gov/entrez/viewer.fcgi?db=nucleotide&val=NM_001702.1) | GDAIF | brain-specific angiogenesis inhibitor 1 |
| BAI3 | [NM_001704.1](http://www.ncbi.nlm.nih.gov/entrez/viewer.fcgi?db=nucleotide&val=NM_001704.1) | - | brain-specific angiogenesis inhibitor 3 |
| BCAS1 | [NM_003657.2](http://www.ncbi.nlm.nih.gov/entrez/viewer.fcgi?db=nucleotide&val=NM_003657.2) | AIBC1\|NABC1 | breast carcinoma amplified sequence 1 |
| BGN | [NM_001711.3](http://www.ncbi.nlm.nih.gov/entrez/viewer.fcgi?db=nucleotide&val=NM_001711.3) | DSPG1\|PG-S1\|PGI\|SLRR1A | biglycan |
| BICC1 | [NM_001080512.1](http://www.ncbi.nlm.nih.gov/entrez/viewer.fcgi?db=nucleotide&val=NM_001080512.1) | BICC\|CYSRD | bicaudal C homolog 1 (Drosophila) |
| BMP4 | [NM_001202.3](http://www.ncbi.nlm.nih.gov/entrez/viewer.fcgi?db=nucleotide&val=NM_001202.3) | BMP2B\|BMP2B1\|MCOPS6\|OFC11\|ZYME | bone morphogenetic protein 4 |
| BMP5 | [NM_021073.2](http://www.ncbi.nlm.nih.gov/entrez/viewer.fcgi?db=nucleotide&val=NM_021073.2) | - | bone morphogenetic protein 5 |
| BMP7 | [NM_001719.2](http://www.ncbi.nlm.nih.gov/entrez/viewer.fcgi?db=nucleotide&val=NM_001719.2) | OP-1 | bone morphogenetic protein 7 |
| BMPER | [NM_133468.4](http://www.ncbi.nlm.nih.gov/entrez/viewer.fcgi?db=nucleotide&val=NM_133468.4) | CRIM3\|CV-2\|CV2 | BMP binding endothelial regulator |
| BMPR1A | [NM_004329.2](http://www.ncbi.nlm.nih.gov/entrez/viewer.fcgi?db=nucleotide&val=NM_004329.2) | 10q23del\|ACVRLK3\|ALK3\|CD292\|SKR5 | bone morphogenetic protein receptor, type IA |
| BMPR1B | [NM_001203.1](http://www.ncbi.nlm.nih.gov/entrez/viewer.fcgi?db=nucleotide&val=NM_001203.1) | ALK-6\|ALK6\|CDw293 | bone morphogenetic protein receptor, type IB |
| BMPR2 | [NM_001204.5](http://www.ncbi.nlm.nih.gov/entrez/viewer.fcgi?db=nucleotide&val=NM_001204.5) | BMPR-II\|BMPR3\|BMR2\|BRK-3\|PPH1\|T-ALK | bone morphogenetic protein receptor, type II (serine/threonine kinase) |
| BNC2 | [NM_017637.5](http://www.ncbi.nlm.nih.gov/entrez/viewer.fcgi?db=nucleotide&val=NM_017637.5) | BSN2 | basonuclin 2 |
| BRMS1 | [NM_015399.3](http://www.ncbi.nlm.nih.gov/entrez/viewer.fcgi?db=nucleotide&val=NM_015399.3) | - | breast cancer metastasis suppressor 1 |
| BTG1 | [NM_001731.2](http://www.ncbi.nlm.nih.gov/entrez/viewer.fcgi?db=nucleotide&val=NM_001731.2) | - | B-cell translocation gene 1, anti-proliferative |
| C1S | [NM_001734.2](http://www.ncbi.nlm.nih.gov/entrez/viewer.fcgi?db=nucleotide&val=NM_001734.2) | - | complement component 1, s subcomponent |
| C3 | [NM_000064.2](http://www.ncbi.nlm.nih.gov/entrez/viewer.fcgi?db=nucleotide&val=NM_000064.2) | AHUS5\|ARMD9\|ASP\|CPAMD1 | complement component 3 |
| C3AR1 | [NM_004054.2](http://www.ncbi.nlm.nih.gov/entrez/viewer.fcgi?db=nucleotide&val=NM_004054.2) | AZ3B\|C3AR\|HNFAG09 | complement component 3a receptor 1 |
| CADM1 | [NM_014333.3](http://www.ncbi.nlm.nih.gov/entrez/viewer.fcgi?db=nucleotide&val=NM_014333.3) | BL2\|IGSF4\|IGSF4A\|NECL2\|Necl-2\|RA175\|ST17\|SYNCAM\|TSLC1\|sTSLC-1\|sgIGSF\|synCAM1 | cell adhesion molecule 1 |
| CALCRL | [NM_005795.3](http://www.ncbi.nlm.nih.gov/entrez/viewer.fcgi?db=nucleotide&val=NM_005795.3) | CGRPR\|CRLR | calcitonin receptor-like |
| CALD1 | [NM_004342.6](http://www.ncbi.nlm.nih.gov/entrez/viewer.fcgi?db=nucleotide&val=NM_004342.6) | CDM\|H-CAD\|HCAD\|L-CAD\|LCAD\|NAG22 | caldesmon 1 |
| CAMK2A | [NM_171825.1](http://www.ncbi.nlm.nih.gov/entrez/viewer.fcgi?db=nucleotide&val=NM_171825.1) | CAMKA | calcium/calmodulin-dependent protein kinase II alpha |
| CAMK2B | [NM_001220.3](http://www.ncbi.nlm.nih.gov/entrez/viewer.fcgi?db=nucleotide&val=NM_001220.3) | CAM2\|CAMK2\|CAMKB | calcium/calmodulin-dependent protein kinase II beta |
| CAMK2D | [NM_172127.1](http://www.ncbi.nlm.nih.gov/entrez/viewer.fcgi?db=nucleotide&val=NM_172127.1) | CAMKD | calcium/calmodulin-dependent protein kinase II delta |
| CAMP | [NM_004345.3](http://www.ncbi.nlm.nih.gov/entrez/viewer.fcgi?db=nucleotide&val=NM_004345.3) | CAP-18\|CAP18\|CRAMP\|FALL-39\|FALL39\|LL37 | cathelicidin antimicrobial peptide |
| CASP8 | [NM_001228.4](http://www.ncbi.nlm.nih.gov/entrez/viewer.fcgi?db=nucleotide&val=NM_001228.4) | ALPS2B\|CAP4\|Casp-8\|FLICE\|MACH\|MCH5 | caspase 8, apoptosis-related cysteine peptidase |
| CAV1 | [NM_001753.3](http://www.ncbi.nlm.nih.gov/entrez/viewer.fcgi?db=nucleotide&val=NM_001753.3) | BSCL3\|CGL3\|MSTP085\|VIP21 | caveolin 1, caveolae protein, 22kDa |
| CBLC | [NM_012116.3](http://www.ncbi.nlm.nih.gov/entrez/viewer.fcgi?db=nucleotide&val=NM_012116.3) | CBL-3\|CBL-SL\|RNF57 | Cbl proto-oncogene, E3 ubiquitin protein ligase C |
| CCBE1 | [NM_133459.3](http://www.ncbi.nlm.nih.gov/entrez/viewer.fcgi?db=nucleotide&val=NM_133459.3) | - | collagen and calcium binding EGF domains 1 |
| CCDC80 | [NM_199511.1](http://www.ncbi.nlm.nih.gov/entrez/viewer.fcgi?db=nucleotide&val=NM_199511.1) | DRO1\|SSG1\|URB\|okuribin | coiled-coil domain containing 80 |
| CCL11 | [NM_002986.2](http://www.ncbi.nlm.nih.gov/entrez/viewer.fcgi?db=nucleotide&val=NM_002986.2) | SCYA11 | chemokine (C-C motif) ligand 11 |
| CCL21 | [NM_002989.2](http://www.ncbi.nlm.nih.gov/entrez/viewer.fcgi?db=nucleotide&val=NM_002989.2) | 6Ckine\|CKb9\|ECL\|SCYA21\|SLC\|TCA4 | chemokine (C-C motif) ligand 21 |
| CCL5 | [NM_002985.2](http://www.ncbi.nlm.nih.gov/entrez/viewer.fcgi?db=nucleotide&val=NM_002985.2) | D17S136E\|RANTES\|SCYA5\|SISd\|TCP228 | chemokine (C-C motif) ligand 5 |
| CCL7 | [NM_006273.2](http://www.ncbi.nlm.nih.gov/entrez/viewer.fcgi?db=nucleotide&val=NM_006273.2) | FIC\|MARC\|MCP-3\|MCP3\|NC28\|SCYA6\|SCYA7 | chemokine (C-C motif) ligand 7 |
| CCL8 | [NM_005623.2](http://www.ncbi.nlm.nih.gov/entrez/viewer.fcgi?db=nucleotide&val=NM_005623.2) | HC14\|MCP-2\|MCP2\|SCYA10\|SCYA8 | chemokine (C-C motif) ligand 8 |
| CCR2 | [NM_001123041.2](http://www.ncbi.nlm.nih.gov/entrez/viewer.fcgi?db=nucleotide&val=NM_001123041.2) | CC-CKR-2\|CCR-2\|CCR2A\|CCR2B\|CD192\|CKR2\|CKR2A\|CKR2B\|CMKBR2\|MCP-1-R | chemokine (C-C motif) receptor 2 |
| CCR3 | [NM_001837.2](http://www.ncbi.nlm.nih.gov/entrez/viewer.fcgi?db=nucleotide&val=NM_001837.2) | CC-CKR-3\|CD193\|CKR3\|CMKBR3 | chemokine (C-C motif) receptor 3 |
| CD163 | [NM_004244.4](http://www.ncbi.nlm.nih.gov/entrez/viewer.fcgi?db=nucleotide&val=NM_004244.4) | M130\|MM130 | CD163 molecule |
| CD24 | [NM_013230.2](http://www.ncbi.nlm.nih.gov/entrez/viewer.fcgi?db=nucleotide&val=NM_013230.2) | CD24A | CD24 molecule |
| CD2AP | [NM_012120.2](http://www.ncbi.nlm.nih.gov/entrez/viewer.fcgi?db=nucleotide&val=NM_012120.2) | CMS | CD2-associated protein |
| CD34 | [NM_001773.2](http://www.ncbi.nlm.nih.gov/entrez/viewer.fcgi?db=nucleotide&val=NM_001773.2) | - | CD34 molecule |
| CD36 | [NM_000072.3](http://www.ncbi.nlm.nih.gov/entrez/viewer.fcgi?db=nucleotide&val=NM_000072.3) | BDPLT10\|CHDS7\|FAT\|GP3B\|GP4\|GPIV\|PASIV\|SCARB3 | CD36 molecule (thrombospondin receptor) |
| CD44 | [NM_001001392.1](http://www.ncbi.nlm.nih.gov/entrez/viewer.fcgi?db=nucleotide&val=NM_001001392.1) | CDW44\|CSPG8\|ECMR-III\|HCELL\|HUTCH-I\|IN\|LHR\|MC56\|MDU2\|MDU3\|MIC4\|Pgp1 | CD44 molecule (Indian blood group) |
| CD46 | [NM_172350.1](http://www.ncbi.nlm.nih.gov/entrez/viewer.fcgi?db=nucleotide&val=NM_172350.1) | AHUS2\|MCP\|MIC10\|TLX\|TRA2.10 | CD46 molecule, complement regulatory protein |
| CD82 | [NM_002231.3](http://www.ncbi.nlm.nih.gov/entrez/viewer.fcgi?db=nucleotide&val=NM_002231.3) | 4F9\|C33\|GR15\|IA4\|KAI1\|R2\|SAR2\|ST6\|TSPAN27 | CD82 molecule |
| CDC42 | [NM_001039802.1](http://www.ncbi.nlm.nih.gov/entrez/viewer.fcgi?db=nucleotide&val=NM_001039802.1) | CDC42Hs\|G25K | cell division cycle 42 (GTP binding protein, 25kDa) |
| CDH1 | [NM_004360.2](http://www.ncbi.nlm.nih.gov/entrez/viewer.fcgi?db=nucleotide&val=NM_004360.2) | Arc-1\|CD324\|CDHE\|ECAD\|LCAM\|UVO | cadherin 1, type 1, E-cadherin (epithelial) |
| CDH11 | [NM_001797.2](http://www.ncbi.nlm.nih.gov/entrez/viewer.fcgi?db=nucleotide&val=NM_001797.2) | CAD11\|CDHOB\|OB\|OSF-4 | cadherin 11, type 2, OB-cadherin (osteoblast) |
| CDH13 | [NM_001220488.1](http://www.ncbi.nlm.nih.gov/entrez/viewer.fcgi?db=nucleotide&val=NM_001220488.1) | CDHH\|P105 | cadherin 13, H-cadherin (heart) |
| CDH2 | [NM_001792.3](http://www.ncbi.nlm.nih.gov/entrez/viewer.fcgi?db=nucleotide&val=NM_001792.3) | CD325\|CDHN\|CDw325\|NCAD | cadherin 2, type 1, N-cadherin (neuronal) |
| CDK14 | [NM_012395.2](http://www.ncbi.nlm.nih.gov/entrez/viewer.fcgi?db=nucleotide&val=NM_012395.2) | PFTAIRE1\|PFTK1 | cyclin-dependent kinase 14 |
| CDKN1A | [NM_000389.2](http://www.ncbi.nlm.nih.gov/entrez/viewer.fcgi?db=nucleotide&val=NM_000389.2) | CAP20\|CDKN1\|CIP1\|MDA-6\|P21\|SDI1\|WAF1\|p21CIP1 | cyclin-dependent kinase inhibitor 1A (p21, Cip1) |
| CDKN2A | [NM_000077.3](http://www.ncbi.nlm.nih.gov/entrez/viewer.fcgi?db=nucleotide&val=NM_000077.3) | ARF\|CDK4I\|CDKN2\|CMM2\|INK4\|INK4A\|MLM\|MTS-1\|MTS1\|P14\|P14ARF\|P16\|P16-INK4A\|P16INK4\|P16INK4A\|P19\|P19ARF\|TP16 | cyclin-dependent kinase inhibitor 2A |
| CDS1 | [NM_001263.3](http://www.ncbi.nlm.nih.gov/entrez/viewer.fcgi?db=nucleotide&val=NM_001263.3) | CDS | CDP-diacylglycerol synthase (phosphatidate cytidylyltransferase) 1 |
| CEACAM1 | [NM_001712.3](http://www.ncbi.nlm.nih.gov/entrez/viewer.fcgi?db=nucleotide&val=NM_001712.3) | BGP\|BGP1\|BGPI | carcinoembryonic antigen-related cell adhesion molecule 1 (biliary glycoprotein) |
| CEACAM5 | [NM_004363.2](http://www.ncbi.nlm.nih.gov/entrez/viewer.fcgi?db=nucleotide&val=NM_004363.2) | CD66e\|CEA | carcinoembryonic antigen-related cell adhesion molecule 5 |
| CEACAM6 | [NM_002483.4](http://www.ncbi.nlm.nih.gov/entrez/viewer.fcgi?db=nucleotide&val=NM_002483.4) | CD66c\|CEAL\|NCA | carcinoembryonic antigen-related cell adhesion molecule 6 (non-specific cross reacting antigen) |
| CEP170 | [NM_001042404.1](http://www.ncbi.nlm.nih.gov/entrez/viewer.fcgi?db=nucleotide&val=NM_001042404.1) | FAM68A\|KAB\|KIAA0470 | centrosomal protein 170kDa |
| CEP295 | [NM_033395.1](http://www.ncbi.nlm.nih.gov/entrez/viewer.fcgi?db=nucleotide&val=NM_033395.1) | #N/A | #N/A |
| CFP | [NM_002621.2](http://www.ncbi.nlm.nih.gov/entrez/viewer.fcgi?db=nucleotide&val=NM_002621.2) | BFD\|PFC\|PFD\|PROPERDIN | complement factor properdin |
| CGN | [NM_020770.2](http://www.ncbi.nlm.nih.gov/entrez/viewer.fcgi?db=nucleotide&val=NM_020770.2) | - | cingulin |
| CHAD | [NM_001267.2](http://www.ncbi.nlm.nih.gov/entrez/viewer.fcgi?db=nucleotide&val=NM_001267.2) | SLRR4A | chondroadherin |
| CHD4 | [NM_001273.2](http://www.ncbi.nlm.nih.gov/entrez/viewer.fcgi?db=nucleotide&val=NM_001273.2) | Mi-2b\|Mi2-BETA | chromodomain helicase DNA binding protein 4 |
| CHI3L1 | [NM_001276.2](http://www.ncbi.nlm.nih.gov/entrez/viewer.fcgi?db=nucleotide&val=NM_001276.2) | ASRT7\|CGP-39\|GP-39\|GP39\|HC-gp39\|HCGP-3P\|YKL-40\|YKL40\|YYL-40\|hCGP-39 | chitinase 3-like 1 (cartilage glycoprotein-39) |
| CHP1 | [XM_005254140.1](http://www.ncbi.nlm.nih.gov/entrez/viewer.fcgi?db=nucleotide&val=XM_005254140.1) | CHP\|SLC9A1BP\|Sid470p\|p22\|p24 | calcineurin-like EF hand protein 1 |
| CHP2 | [NM_022097.3](http://www.ncbi.nlm.nih.gov/entrez/viewer.fcgi?db=nucleotide&val=NM_022097.3) | - | calcineurin-like EF hand protein 2 |
| CHRDL1 | [NM_001143981.1](http://www.ncbi.nlm.nih.gov/entrez/viewer.fcgi?db=nucleotide&val=NM_001143981.1) | CHL\|NRLN1\|VOPT\|dA141H5.1 | chordin-like 1 |
| CHRNA7 | [NR_046324.1](http://www.ncbi.nlm.nih.gov/entrez/viewer.fcgi?db=nucleotide&val=NR_046324.1) | CHRNA7-2\|NACHRA7 | cholinergic receptor, nicotinic, alpha 7 (neuronal) |
| CIB1 | [NM_001277764.1](http://www.ncbi.nlm.nih.gov/entrez/viewer.fcgi?db=nucleotide&val=NM_001277764.1) | CIB\|KIP\|KIP1\|SIP2-28 | calcium and integrin binding 1 (calmyrin) |
| CKMT1A | [NM_001015001.1](http://www.ncbi.nlm.nih.gov/entrez/viewer.fcgi?db=nucleotide&val=NM_001015001.1) | CKMT1 | creatine kinase, mitochondrial 1A |
| CLDN1 | [NM_021101.3](http://www.ncbi.nlm.nih.gov/entrez/viewer.fcgi?db=nucleotide&val=NM_021101.3) | CLD1\|ILVASC\|SEMP1 | claudin 1 |
| CLDN3 | [NM_001306.3](http://www.ncbi.nlm.nih.gov/entrez/viewer.fcgi?db=nucleotide&val=NM_001306.3) | C7orf1\|CPE-R2\|CPETR2\|HRVP1\|RVP1 | claudin 3 |
| CLDN4 | [NM_001305.3](http://www.ncbi.nlm.nih.gov/entrez/viewer.fcgi?db=nucleotide&val=NM_001305.3) | CPE-R\|CPER\|CPETR\|CPETR1\|WBSCR8\|hCPE-R | claudin 4 |
| CLDN7 | [NM_001307.3](http://www.ncbi.nlm.nih.gov/entrez/viewer.fcgi?db=nucleotide&val=NM_001307.3) | CEPTRL2\|CLDN-7\|CPETRL2\|Hs.84359\|claudin-1 | claudin 7 |
| CLEC2B | [NM_005127.2](http://www.ncbi.nlm.nih.gov/entrez/viewer.fcgi?db=nucleotide&val=NM_005127.2) | AICL\|CLECSF2\|HP10085\|IFNRG1 | C-type lectin domain family 2, member B |
| CLEC3B | [NM_003278.2](http://www.ncbi.nlm.nih.gov/entrez/viewer.fcgi?db=nucleotide&val=NM_003278.2) | TN\|TNA | C-type lectin domain family 3, member B |
| CLIC4 | [NM_013943.2](http://www.ncbi.nlm.nih.gov/entrez/viewer.fcgi?db=nucleotide&val=NM_013943.2) | CLIC4L\|H1\|MTCLIC\|huH1\|p64H1 | chloride intracellular channel 4 |
| CLU | [NM_203339.2](http://www.ncbi.nlm.nih.gov/entrez/viewer.fcgi?db=nucleotide&val=NM_203339.2) | APO-J\|APOJ\|CLI\|KUB1\|NA1/NA2\|SGP-2\|SGP2\|SP-40\|TRPM-2\|TRPM2 | clusterin |
| CMA1 | [NM_001836.2](http://www.ncbi.nlm.nih.gov/entrez/viewer.fcgi?db=nucleotide&val=NM_001836.2) | CYH\|MCT1\|chymase | chymase 1, mast cell |
| CNN1 | [NM_001299.4](http://www.ncbi.nlm.nih.gov/entrez/viewer.fcgi?db=nucleotide&val=NM_001299.4) | SMCC\|Sm-Calp | calponin 1, basic, smooth muscle |
| COL18A1 | [NM_030582.3](http://www.ncbi.nlm.nih.gov/entrez/viewer.fcgi?db=nucleotide&val=NM_030582.3) | KNO\|KNO1\|KS | collagen, type XVIII, alpha 1 |
| COL1A1 | [NM_000088.3](http://www.ncbi.nlm.nih.gov/entrez/viewer.fcgi?db=nucleotide&val=NM_000088.3) | OI4 | collagen, type I, alpha 1 |
| COL1A2 | [NM_000089.3](http://www.ncbi.nlm.nih.gov/entrez/viewer.fcgi?db=nucleotide&val=NM_000089.3) | OI4 | collagen, type I, alpha 2 |
| COL3A1 | [NM_000090.3](http://www.ncbi.nlm.nih.gov/entrez/viewer.fcgi?db=nucleotide&val=NM_000090.3) | EDS4A | collagen, type III, alpha 1 |
| COL4A1 | [NM_001845.4](http://www.ncbi.nlm.nih.gov/entrez/viewer.fcgi?db=nucleotide&val=NM_001845.4) | HANAC\|POREN1\|arresten | collagen, type IV, alpha 1 |
| COL4A2 | [NM_001846.2](http://www.ncbi.nlm.nih.gov/entrez/viewer.fcgi?db=nucleotide&val=NM_001846.2) | ICH\|POREN2 | collagen, type IV, alpha 2 |
| COL4A6 | [NM_001847.2](http://www.ncbi.nlm.nih.gov/entrez/viewer.fcgi?db=nucleotide&val=NM_001847.2) | CXDELq22.3\|DELXq22.3 | collagen, type IV, alpha 6 |
| COL5A1 | [NM_000093.3](http://www.ncbi.nlm.nih.gov/entrez/viewer.fcgi?db=nucleotide&val=NM_000093.3) | - | collagen, type V, alpha 1 |
| COL5A2 | [NM_000393.3](http://www.ncbi.nlm.nih.gov/entrez/viewer.fcgi?db=nucleotide&val=NM_000393.3) | - | collagen, type V, alpha 2 |
| COL6A1 | [NM_001848.2](http://www.ncbi.nlm.nih.gov/entrez/viewer.fcgi?db=nucleotide&val=NM_001848.2) | OPLL | collagen, type VI, alpha 1 |
| COL6A2 | [NM_001849.2](http://www.ncbi.nlm.nih.gov/entrez/viewer.fcgi?db=nucleotide&val=NM_001849.2) | PP3610 | collagen, type VI, alpha 2 |
| COL6A3 | [NM_004369.3](http://www.ncbi.nlm.nih.gov/entrez/viewer.fcgi?db=nucleotide&val=NM_004369.3) | - | collagen, type VI, alpha 3 |
| COL7A1 | [NM_000094.2](http://www.ncbi.nlm.nih.gov/entrez/viewer.fcgi?db=nucleotide&val=NM_000094.2) | EBD1\|EBDCT\|EBR1 | collagen, type VII, alpha 1 |
| COMP | [NM_000095.2](http://www.ncbi.nlm.nih.gov/entrez/viewer.fcgi?db=nucleotide&val=NM_000095.2) | EDM1\|EPD1\|MED\|PSACH\|THBS5 | cartilage oligomeric matrix protein |
| CREBBP | [NM_001079846.1](http://www.ncbi.nlm.nih.gov/entrez/viewer.fcgi?db=nucleotide&val=NM_001079846.1) | CBP\|KAT3A\|RSTS | CREB binding protein |
| CRIP2 | [NM_001270837.1](http://www.ncbi.nlm.nih.gov/entrez/viewer.fcgi?db=nucleotide&val=NM_001270837.1) | CRIP\|CRP2\|ESP1 | cysteine-rich protein 2 |
| CRISPLD2 | [NM_031476.3](http://www.ncbi.nlm.nih.gov/entrez/viewer.fcgi?db=nucleotide&val=NM_031476.3) | CRISP11\|LCRISP2 | cysteine-rich secretory protein LCCL domain containing 2 |
| CSF2RB | [NM_000395.2](http://www.ncbi.nlm.nih.gov/entrez/viewer.fcgi?db=nucleotide&val=NM_000395.2) | CD131\|CDw131\|IL3RB\|IL5RB\|SMDP5 | colony stimulating factor 2 receptor, beta, low-affinity (granulocyte-macrophage) |
| CSPG4 | [NM_001897.4](http://www.ncbi.nlm.nih.gov/entrez/viewer.fcgi?db=nucleotide&val=NM_001897.4) | HMW-MAA\|MCSP\|MCSPG\|MEL-CSPG\|MSK16\|NG2 | chondroitin sulfate proteoglycan 4 |
| CST7 | [NM_003650.3](http://www.ncbi.nlm.nih.gov/entrez/viewer.fcgi?db=nucleotide&val=NM_003650.3) | CMAP | cystatin F (leukocystatin) |
| CTNNB1 | [NM_001098210.1](http://www.ncbi.nlm.nih.gov/entrez/viewer.fcgi?db=nucleotide&val=NM_001098210.1) | CTNNB | catenin (cadherin-associated protein), beta 1, 88kDa |
| CTNND1 | [NM_001331.2](http://www.ncbi.nlm.nih.gov/entrez/viewer.fcgi?db=nucleotide&val=NM_001331.2) | CAS\|CTNND\|P120CAS\|P120CTN\|p120\|p120(CAS)\|p120(CTN) | catenin (cadherin-associated protein), delta 1 |
| CTSG | [NM_001911.2](http://www.ncbi.nlm.nih.gov/entrez/viewer.fcgi?db=nucleotide&val=NM_001911.2) | CATG\|CG | cathepsin G |
| CTSH | [NM_148979.2](http://www.ncbi.nlm.nih.gov/entrez/viewer.fcgi?db=nucleotide&val=NM_148979.2) | ACC-4\|ACC-5\|CPSB\|minichain | cathepsin H |
| CTSK | [NM_000396.2](http://www.ncbi.nlm.nih.gov/entrez/viewer.fcgi?db=nucleotide&val=NM_000396.2) | CTS02\|CTSO\|CTSO1\|CTSO2\|PKND\|PYCD | cathepsin K |
| CTSL | [NM_001912.4](http://www.ncbi.nlm.nih.gov/entrez/viewer.fcgi?db=nucleotide&val=NM_001912.4) | #N/A | #N/A |
| CUL1 | [NM_003592.2](http://www.ncbi.nlm.nih.gov/entrez/viewer.fcgi?db=nucleotide&val=NM_003592.2) | - | cullin 1 |
| CX3CL1 | [NM_002996.3](http://www.ncbi.nlm.nih.gov/entrez/viewer.fcgi?db=nucleotide&val=NM_002996.3) | ABCD-3\|C3Xkine\|CXC3\|CXC3C\|NTN\|NTT\|SCYD1\|fractalkine\|neurotactin | chemokine (C-X3-C motif) ligand 1 |
| CXADR | [NM_001338.3](http://www.ncbi.nlm.nih.gov/entrez/viewer.fcgi?db=nucleotide&val=NM_001338.3) | CAR\|CAR4/6\|HCAR | coxsackie virus and adenovirus receptor |
| CXCL10 | [NM_001565.1](http://www.ncbi.nlm.nih.gov/entrez/viewer.fcgi?db=nucleotide&val=NM_001565.1) | C7\|IFI10\|INP10\|IP-10\|SCYB10\|crg-2\|gIP-10\|mob-1 | chemokine (C-X-C motif) ligand 10 |
| CXCL11 | [NM_005409.3](http://www.ncbi.nlm.nih.gov/entrez/viewer.fcgi?db=nucleotide&val=NM_005409.3) | H174\|I-TAC\|IP-9\|IP9\|SCYB11\|SCYB9B\|b-R1 | chemokine (C-X-C motif) ligand 11 |
| CXCL12 | [NM_199168.3](http://www.ncbi.nlm.nih.gov/entrez/viewer.fcgi?db=nucleotide&val=NM_199168.3) | IRH\|PBSF\|SCYB12\|SDF1\|SDF1A\|SDF1B\|TLSF\|TPAR1 | chemokine (C-X-C motif) ligand 12 |
| CXCL13 | [NM_006419.2](http://www.ncbi.nlm.nih.gov/entrez/viewer.fcgi?db=nucleotide&val=NM_006419.2) | ANGIE\|ANGIE2\|BCA-1\|BCA1\|BLC\|BLR1L\|SCYB13 | chemokine (C-X-C motif) ligand 13 |
| CXCL17 | [NM_198477.1](http://www.ncbi.nlm.nih.gov/entrez/viewer.fcgi?db=nucleotide&val=NM_198477.1) | DMC\|Dcip1\|UNQ473\|VCC-1\|VCC1 | chemokine (C-X-C motif) ligand 17 |
| CXCL8 | [NM_000584.2](http://www.ncbi.nlm.nih.gov/entrez/viewer.fcgi?db=nucleotide&val=NM_000584.2) | #N/A | #N/A |
| CXCR2 | [NM_001168298.1](http://www.ncbi.nlm.nih.gov/entrez/viewer.fcgi?db=nucleotide&val=NM_001168298.1) | CD182\|CDw128b\|CMKAR2\|IL8R2\|IL8RA\|IL8RB | chemokine (C-X-C motif) receptor 2 |
| CXCR3 | [NM_001504.1](http://www.ncbi.nlm.nih.gov/entrez/viewer.fcgi?db=nucleotide&val=NM_001504.1) | CD182\|CD183\|CKR-L2\|CMKAR3\|GPR9\|IP10-R\|Mig-R\|MigR | chemokine (C-X-C motif) receptor 3 |
| CXCR4 | [NM_003467.2](http://www.ncbi.nlm.nih.gov/entrez/viewer.fcgi?db=nucleotide&val=NM_003467.2) | CD184\|D2S201E\|FB22\|HM89\|HSY3RR\|LAP3\|LCR1\|LESTR\|NPY3R\|NPYR\|NPYRL\|NPYY3R\|WHIM | chemokine (C-X-C motif) receptor 4 |
| CYB561 | [NM_001915.3](http://www.ncbi.nlm.nih.gov/entrez/viewer.fcgi?db=nucleotide&val=NM_001915.3) | FRRS2 | cytochrome b-561 |
| CYBB | [NM_000397.3](http://www.ncbi.nlm.nih.gov/entrez/viewer.fcgi?db=nucleotide&val=NM_000397.3) | AMCBX2\|CGD\|GP91-1\|GP91-PHOX\|GP91PHOX\|NOX2\|p91-PHOX | cytochrome b-245, beta polypeptide |
| CYP1B1 | [NM_000104.3](http://www.ncbi.nlm.nih.gov/entrez/viewer.fcgi?db=nucleotide&val=NM_000104.3) | CP1B\|CYPIB1\|GLC3A\|P4501B1 | cytochrome P450, family 1, subfamily B, polypeptide 1 |
| DAG1 | [NM_001165928.2](http://www.ncbi.nlm.nih.gov/entrez/viewer.fcgi?db=nucleotide&val=NM_001165928.2) | 156DAG\|A3a\|AGRNR\|DAG\|MDDGC7 | dystroglycan 1 (dystrophin-associated glycoprotein 1) |
| DCC | [NM_005215.1](http://www.ncbi.nlm.nih.gov/entrez/viewer.fcgi?db=nucleotide&val=NM_005215.1) | CRC18\|CRCR1\|IGDCC1 | deleted in colorectal carcinoma |
| DCN | [NM_001920.3](http://www.ncbi.nlm.nih.gov/entrez/viewer.fcgi?db=nucleotide&val=NM_001920.3) | CSCD\|DSPG2\|PG40\|PGII\|PGS2\|SLRR1B | decorin |
| DDR2 | [NM_006182.2](http://www.ncbi.nlm.nih.gov/entrez/viewer.fcgi?db=nucleotide&val=NM_006182.2) | MIG20a\|NTRKR3\|TKT\|TYRO10 | discoidin domain receptor tyrosine kinase 2 |
| DENND5A | [NM_015213.2](http://www.ncbi.nlm.nih.gov/entrez/viewer.fcgi?db=nucleotide&val=NM_015213.2) | RAB6IP1 | DENN/MADD domain containing 5A |
| DENR | [NM_003677.3](http://www.ncbi.nlm.nih.gov/entrez/viewer.fcgi?db=nucleotide&val=NM_003677.3) | DRP\|DRP1\|SMAP-3 | density-regulated protein |
| DESI1 | [NM_015704.2](http://www.ncbi.nlm.nih.gov/entrez/viewer.fcgi?db=nucleotide&val=NM_015704.2) | D15Wsu75e\|DESI2\|DJ347H13.4\|DeSI-1\|FAM152B\|PPPDE2 | desumoylating isopeptidase 1 |
| DICER1 | [NM_177438.2](http://www.ncbi.nlm.nih.gov/entrez/viewer.fcgi?db=nucleotide&val=NM_177438.2) | DCR1\|Dicer\|HERNA\|MNG1 | dicer 1, ribonuclease type III |
| DLC1 | [NM_006094.3](http://www.ncbi.nlm.nih.gov/entrez/viewer.fcgi?db=nucleotide&val=NM_006094.3) | ARHGAP7\|HP\|STARD12\|p122-RhoGAP | deleted in liver cancer 1 |
| DLG1 | [NM_001098424.1](http://www.ncbi.nlm.nih.gov/entrez/viewer.fcgi?db=nucleotide&val=NM_001098424.1) | DLGH1\|SAP-97\|SAP97\|dJ1061C18.1.1\|hdlg | discs, large homolog 1 (Drosophila) |
| DLL4 | [NM_019074.2](http://www.ncbi.nlm.nih.gov/entrez/viewer.fcgi?db=nucleotide&val=NM_019074.2) | hdelta2 | delta-like 4 (Drosophila) |
| DPT | [NM_001937.3](http://www.ncbi.nlm.nih.gov/entrez/viewer.fcgi?db=nucleotide&val=NM_001937.3) | TRAMP | dermatopontin |
| DPYSL3 | [NM_001387.2](http://www.ncbi.nlm.nih.gov/entrez/viewer.fcgi?db=nucleotide&val=NM_001387.2) | CRMP-4\|CRMP4\|DRP-3\|DRP3\|LCRMP\|ULIP\|ULIP-1 | dihydropyrimidinase-like 3 |
| DSC2 | [NM_024422.3](http://www.ncbi.nlm.nih.gov/entrez/viewer.fcgi?db=nucleotide&val=NM_024422.3) | ARVD11\|CDHF2\|DG2\|DGII/III\|DSC3 | desmocollin 2 |
| DST | [NM_001723.4](http://www.ncbi.nlm.nih.gov/entrez/viewer.fcgi?db=nucleotide&val=NM_001723.4) | BP240\|BPA\|BPAG1\|CATX-15\|CATX15\|D6S1101\|DMH\|DT\|HSAN6\|MACF2 | dystonin |
| ECM1 | [NM_004425.3](http://www.ncbi.nlm.nih.gov/entrez/viewer.fcgi?db=nucleotide&val=NM_004425.3) | - | extracellular matrix protein 1 |
| ECM2 | [NM_001393.2](http://www.ncbi.nlm.nih.gov/entrez/viewer.fcgi?db=nucleotide&val=NM_001393.2) | - | extracellular matrix protein 2, female organ and adipocyte specific |
| ECSCR | [NM_001077693.3](http://www.ncbi.nlm.nih.gov/entrez/viewer.fcgi?db=nucleotide&val=NM_001077693.3) | ARIA\|ECSM2 | endothelial cell surface expressed chemotaxis and apoptosis regulator |
| EDN1 | [NM_001955.2](http://www.ncbi.nlm.nih.gov/entrez/viewer.fcgi?db=nucleotide&val=NM_001955.2) | ET1\|HDLCQ7\|PPET1 | endothelin 1 |
| EGF | [NM_001963.4](http://www.ncbi.nlm.nih.gov/entrez/viewer.fcgi?db=nucleotide&val=NM_001963.4) | HOMG4\|URG | epidermal growth factor |
| EGFL7 | [NM_016215.3](http://www.ncbi.nlm.nih.gov/entrez/viewer.fcgi?db=nucleotide&val=NM_016215.3) | NEU1\|RP11-251M1.2\|VE-STATIN\|ZNEU1 | EGF-like-domain, multiple 7 |
| EGFR | [NM_201282.1](http://www.ncbi.nlm.nih.gov/entrez/viewer.fcgi?db=nucleotide&val=NM_201282.1) | ERBB\|ERBB1\|HER1\|PIG61\|mENA | epidermal growth factor receptor |
| EGLN2 | [NM_053046.3](http://www.ncbi.nlm.nih.gov/entrez/viewer.fcgi?db=nucleotide&val=NM_053046.3) | EIT6\|HIF-PH1\|HIFPH1\|HPH-1\|HPH-3\|PHD1 | egl nine homolog 2 (C. elegans) |
| EGLN3 | [NM_022073.3](http://www.ncbi.nlm.nih.gov/entrez/viewer.fcgi?db=nucleotide&val=NM_022073.3) | HIFP4H3\|HIFPH3\|PHD3 | egl nine homolog 3 (C. elegans) |
| EIF2AK3 | [NM_004836.5](http://www.ncbi.nlm.nih.gov/entrez/viewer.fcgi?db=nucleotide&val=NM_004836.5) | PEK\|PERK\|WRS | eukaryotic translation initiation factor 2-alpha kinase 3 |
| EIF4E2 | [NM_004846.3](http://www.ncbi.nlm.nih.gov/entrez/viewer.fcgi?db=nucleotide&val=NM_004846.3) | 4E-LP\|4EHP\|EIF4EL3\|IF4e | eukaryotic translation initiation factor 4E family member 2 |
| EIF4EBP1 | [NM_004095.3](http://www.ncbi.nlm.nih.gov/entrez/viewer.fcgi?db=nucleotide&val=NM_004095.3) | 4E-BP1\|4EBP1\|BP-1\|PHAS-I | eukaryotic translation initiation factor 4E binding protein 1 |
| ELF3 | [NM_001114309.1](http://www.ncbi.nlm.nih.gov/entrez/viewer.fcgi?db=nucleotide&val=NM_001114309.1) | EPR-1\|ERT\|ESE-1\|ESX | E74-like factor 3 (ets domain transcription factor, epithelial-specific ) |
| ELK3 | [NM_005230.2](http://www.ncbi.nlm.nih.gov/entrez/viewer.fcgi?db=nucleotide&val=NM_005230.2) | ERP\|NET\|SAP2 | ELK3, ETS-domain protein (SRF accessory protein 2) |
| EMCN | [NM_016242.3](http://www.ncbi.nlm.nih.gov/entrez/viewer.fcgi?db=nucleotide&val=NM_016242.3) | EMCN2\|MUC14 | endomucin |
| EMILIN1 | [XM_006711928.1](http://www.ncbi.nlm.nih.gov/entrez/viewer.fcgi?db=nucleotide&val=XM_006711928.1) | EMI\|EMILIN\|gp115 | elastin microfibril interfacer 1 |
| EMILIN3 | [NM_052846.1](http://www.ncbi.nlm.nih.gov/entrez/viewer.fcgi?db=nucleotide&val=NM_052846.1) | C20orf130\|EMILIN5\|dJ620E11.4 | elastin microfibril interfacer 3 |
| EMP3 | [NM_001425.2](http://www.ncbi.nlm.nih.gov/entrez/viewer.fcgi?db=nucleotide&val=NM_001425.2) | YMP | epithelial membrane protein 3 |
| ENO1 | [NM_001428.2](http://www.ncbi.nlm.nih.gov/entrez/viewer.fcgi?db=nucleotide&val=NM_001428.2) | ENO1L1\|MPB1\|NNE\|PPH | enolase 1, (alpha) |
| ENO2 | [NM_001975.2](http://www.ncbi.nlm.nih.gov/entrez/viewer.fcgi?db=nucleotide&val=NM_001975.2) | NSE | enolase 2 (gamma, neuronal) |
| ENO3 | [NM_001976.4](http://www.ncbi.nlm.nih.gov/entrez/viewer.fcgi?db=nucleotide&val=NM_001976.4) | GSD13\|MSE | enolase 3 (beta, muscle) |
| ENPEP | [NM_001977.3](http://www.ncbi.nlm.nih.gov/entrez/viewer.fcgi?db=nucleotide&val=NM_001977.3) | APA\|CD249\|gp160 | glutamyl aminopeptidase (aminopeptidase A) |
| ENPP2 | [NM_001040092.2](http://www.ncbi.nlm.nih.gov/entrez/viewer.fcgi?db=nucleotide&val=NM_001040092.2) | ATX\|ATX-X\|AUTOTAXIN\|LysoPLD\|NPP2\|PD-IALPHA\|PDNP2 | ectonucleotide pyrophosphatase/phosphodiesterase 2 |
| EP300 | [NM_001429.2](http://www.ncbi.nlm.nih.gov/entrez/viewer.fcgi?db=nucleotide&val=NM_001429.2) | KAT3B\|RSTS2\|p300 | E1A binding protein p300 |
| EPAS1 | [NM_001430.3](http://www.ncbi.nlm.nih.gov/entrez/viewer.fcgi?db=nucleotide&val=NM_001430.3) | ECYT4\|HIF2A\|HLF\|MOP2\|PASD2\|bHLHe73 | endothelial PAS domain protein 1 |
| EPCAM | [NM_002354.1](http://www.ncbi.nlm.nih.gov/entrez/viewer.fcgi?db=nucleotide&val=NM_002354.1) | DIAR5\|EGP-2\|EGP314\|EGP40\|ESA\|HNPCC8\|KS1/4\|KSA\|M4S1\|MIC18\|MK-1\|TACSTD1\|TROP1 | epithelial cell adhesion molecule |
| EPHA1 | [NM_005232.3](http://www.ncbi.nlm.nih.gov/entrez/viewer.fcgi?db=nucleotide&val=NM_005232.3) | EPH\|EPHT\|EPHT1 | EPH receptor A1 |
| EPHA2 | [NM_004431.2](http://www.ncbi.nlm.nih.gov/entrez/viewer.fcgi?db=nucleotide&val=NM_004431.2) | ARCC2\|CTPA\|CTPP1\|ECK | EPH receptor A2 |
| EPHB1 | [NM_004441.3](http://www.ncbi.nlm.nih.gov/entrez/viewer.fcgi?db=nucleotide&val=NM_004441.3) | ELK\|EPHT2\|Hek6\|NET | EPH receptor B1 |
| EPHB3 | [NM_004443.3](http://www.ncbi.nlm.nih.gov/entrez/viewer.fcgi?db=nucleotide&val=NM_004443.3) | ETK2\|HEK2\|TYRO6 | EPH receptor B3 |
| EPHB4 | [NM_004444.4](http://www.ncbi.nlm.nih.gov/entrez/viewer.fcgi?db=nucleotide&val=NM_004444.4) | HTK\|MYK1\|TYRO11 | EPH receptor B4 |
| EPN3 | [NM_017957.2](http://www.ncbi.nlm.nih.gov/entrez/viewer.fcgi?db=nucleotide&val=NM_017957.2) | - | epsin 3 |
| EPS8L1 | [NM_017729.3](http://www.ncbi.nlm.nih.gov/entrez/viewer.fcgi?db=nucleotide&val=NM_017729.3) | DRC3\|EPS8R1 | EPS8-like 1 |
| ERBB2 | [NM_001005862.1](http://www.ncbi.nlm.nih.gov/entrez/viewer.fcgi?db=nucleotide&val=NM_001005862.1) | CD340\|HER-2\|HER-2/neu\|HER2\|MLN 19\|NEU\|NGL\|TKR1 | v-erb-b2 erythroblastic leukemia viral oncogene homolog 2, neuro/glioblastoma derived oncogene homolog (avian) |
| ERBB2IP | [NM_018695.2](http://www.ncbi.nlm.nih.gov/entrez/viewer.fcgi?db=nucleotide&val=NM_018695.2) | ERBIN\|LAP2 | erbb2 interacting protein |
| ERBB3 | [NM_001005915.1](http://www.ncbi.nlm.nih.gov/entrez/viewer.fcgi?db=nucleotide&val=NM_001005915.1) | ErbB-3\|HER3\|LCCS2\|MDA-BF-1\|c-erbB-3\|c-erbB3\|erbB3-S\|p180-ErbB3\|p45-sErbB3\|p85-sErbB3 | v-erb-b2 erythroblastic leukemia viral oncogene homolog 3 (avian) |
| EREG | [NM_001432.2](http://www.ncbi.nlm.nih.gov/entrez/viewer.fcgi?db=nucleotide&val=NM_001432.2) | ER | epiregulin |
| ERMP1 | [NM_024896.2](http://www.ncbi.nlm.nih.gov/entrez/viewer.fcgi?db=nucleotide&val=NM_024896.2) | FXNA\|KIAA1815\|bA207C16.3 | endoplasmic reticulum metallopeptidase 1 |
| ESRP1 | [NM_001034915.2](http://www.ncbi.nlm.nih.gov/entrez/viewer.fcgi?db=nucleotide&val=NM_001034915.2) | RBM35A\|RMB35A | epithelial splicing regulatory protein 1 |
| ETV4 | [NM_001079675.1](http://www.ncbi.nlm.nih.gov/entrez/viewer.fcgi?db=nucleotide&val=NM_001079675.1) | E1A-F\|E1AF\|PEA3\|PEAS3 | ets variant 4 |
| EVI2A | [NM_014210.3](http://www.ncbi.nlm.nih.gov/entrez/viewer.fcgi?db=nucleotide&val=NM_014210.3) | EVDA\|EVI-2A\|EVI2 | ecotropic viral integration site 2A |
| EVPL | [NM_001988.2](http://www.ncbi.nlm.nih.gov/entrez/viewer.fcgi?db=nucleotide&val=NM_001988.2) | EVPK | envoplakin |
| F11R | [NM_144503.1](http://www.ncbi.nlm.nih.gov/entrez/viewer.fcgi?db=nucleotide&val=NM_144503.1) | CD321\|JAM\|JAM1\|JAMA\|JCAM\|KAT\|PAM-1 | F11 receptor |
| F3 | [NM_001993.3](http://www.ncbi.nlm.nih.gov/entrez/viewer.fcgi?db=nucleotide&val=NM_001993.3) | CD142\|TF\|TFA | coagulation factor III (thromboplastin, tissue factor) |
| FAM174B | [NM_207446.2](http://www.ncbi.nlm.nih.gov/entrez/viewer.fcgi?db=nucleotide&val=NM_207446.2) | - | family with sequence similarity 174, member B |
| FAP | [NM_004460.2](http://www.ncbi.nlm.nih.gov/entrez/viewer.fcgi?db=nucleotide&val=NM_004460.2) | DPPIV\|FAPA | fibroblast activation protein, alpha |
| FASLG | [NM_000639.1](http://www.ncbi.nlm.nih.gov/entrez/viewer.fcgi?db=nucleotide&val=NM_000639.1) | ALPS1B\|APT1LG1\|CD178\|CD95-L\|CD95L\|FASL\|TNFSF6 | Fas ligand (TNF superfamily, member 6) |
| FBLN1 | [NM_006487.2](http://www.ncbi.nlm.nih.gov/entrez/viewer.fcgi?db=nucleotide&val=NM_006487.2) | FBLN\|FIBL1 | fibulin 1 |
| FBLN5 | [NM_006329.3](http://www.ncbi.nlm.nih.gov/entrez/viewer.fcgi?db=nucleotide&val=NM_006329.3) | ADCL2\|ARCL1A\|ARMD3\|DANCE\|EVEC\|FIBL-5\|UP50 | fibulin 5 |
| FBN1 | [NM_000138.3](http://www.ncbi.nlm.nih.gov/entrez/viewer.fcgi?db=nucleotide&val=NM_000138.3) | ACMICD\|ECTOL1\|FBN\|GPHYSD2\|MASS\|MFS1\|OCTD\|SGS\|SSKS\|WMS\|WMS2 | fibrillin 1 |
| FBN2 | [NM_001999.3](http://www.ncbi.nlm.nih.gov/entrez/viewer.fcgi?db=nucleotide&val=NM_001999.3) | CCA\|DA9 | fibrillin 2 |
| FBP1 | [NM_000507.3](http://www.ncbi.nlm.nih.gov/entrez/viewer.fcgi?db=nucleotide&val=NM_000507.3) | FBP | fructose-1,6-bisphosphatase 1 |
| FERMT2 | [NM_001135000.1](http://www.ncbi.nlm.nih.gov/entrez/viewer.fcgi?db=nucleotide&val=NM_001135000.1) | KIND2\|MIG2\|PLEKHC1\|UNC112\|UNC112B\|mig-2 | fermitin family member 2 |
| FGF18 | [NM_003862.1](http://www.ncbi.nlm.nih.gov/entrez/viewer.fcgi?db=nucleotide&val=NM_003862.1) | FGF-18\|ZFGF5 | fibroblast growth factor 18 |
| FGF2 | [NM_002006.4](http://www.ncbi.nlm.nih.gov/entrez/viewer.fcgi?db=nucleotide&val=NM_002006.4) | BFGF\|FGF-2\|FGFB\|HBGF-2 | fibroblast growth factor 2 (basic) |
| FGF9 | [NM_002010.2](http://www.ncbi.nlm.nih.gov/entrez/viewer.fcgi?db=nucleotide&val=NM_002010.2) | GAF\|HBFG-9\|SYNS3 | fibroblast growth factor 9 (glia-activating factor) |
| FGFR1 | [NM_015850.2](http://www.ncbi.nlm.nih.gov/entrez/viewer.fcgi?db=nucleotide&val=NM_015850.2) | BFGFR\|CD331\|CEK\|FGFBR\|FGFR-1\|FLG\|FLT-2\|FLT2\|HBGFR\|KAL2\|N-SAM\|OGD\|bFGF-R-1 | fibroblast growth factor receptor 1 |
| FGFR2 | [NM_000141.4](http://www.ncbi.nlm.nih.gov/entrez/viewer.fcgi?db=nucleotide&val=NM_000141.4) | BBDS\|BEK\|BFR-1\|CD332\|CEK3\|CFD1\|ECT1\|JWS\|K-SAM\|KGFR\|TK14\|TK25 | fibroblast growth factor receptor 2 |
| FGFR3 | [NM_022965.2](http://www.ncbi.nlm.nih.gov/entrez/viewer.fcgi?db=nucleotide&val=NM_022965.2) | ACH\|CD333\|CEK2\|HSFGFR3EX\|JTK4 | fibroblast growth factor receptor 3 |
| FGFR4 | [NM_002011.3](http://www.ncbi.nlm.nih.gov/entrez/viewer.fcgi?db=nucleotide&val=NM_002011.3) | CD334\|JTK2\|TKF | fibroblast growth factor receptor 4 |
| FGL2 | [NM_006682.2](http://www.ncbi.nlm.nih.gov/entrez/viewer.fcgi?db=nucleotide&val=NM_006682.2) | T49\|pT49 | fibrinogen-like 2 |
| FHL1 | [NM_001449.4](http://www.ncbi.nlm.nih.gov/entrez/viewer.fcgi?db=nucleotide&val=NM_001449.4) | FHL-1\|FHL1A\|FHL1B\|FLH1A\|KYOT\|SLIM\|SLIM-1\|SLIM1\|SLIMMER\|XMPMA | four and a half LIM domains 1 |
| FIGF | [NM_004469.2](http://www.ncbi.nlm.nih.gov/entrez/viewer.fcgi?db=nucleotide&val=NM_004469.2) | VEGF-D\|VEGFD | c-fos induced growth factor (vascular endothelial growth factor D) |
| FLI1 | [NM_001167681.2](http://www.ncbi.nlm.nih.gov/entrez/viewer.fcgi?db=nucleotide&val=NM_001167681.2) | EWSR2\|SIC-1 | Friend leukemia virus integration 1 |
| FLT1 | [NM_002019.4](http://www.ncbi.nlm.nih.gov/entrez/viewer.fcgi?db=nucleotide&val=NM_002019.4) | FLT\|FLT-1\|VEGFR-1\|VEGFR1 | fms-related tyrosine kinase 1 (vascular endothelial growth factor/vascular permeability factor receptor) |
| FLT4 | [NM_002020.1](http://www.ncbi.nlm.nih.gov/entrez/viewer.fcgi?db=nucleotide&val=NM_002020.1) | FLT41\|LMPH1A\|PCL\|VEGFR3 | fms-related tyrosine kinase 4 |
| FMOD | [NM_002023.3](http://www.ncbi.nlm.nih.gov/entrez/viewer.fcgi?db=nucleotide&val=NM_002023.3) | SLRR2E | fibromodulin |
| FN1 | [NM_212482.1](http://www.ncbi.nlm.nih.gov/entrez/viewer.fcgi?db=nucleotide&val=NM_212482.1) | CIG\|ED-B\|FINC\|FN\|FNZ\|GFND\|GFND2\|LETS\|MSF | fibronectin 1 |
| FOXC2 | [NM_005251.2](http://www.ncbi.nlm.nih.gov/entrez/viewer.fcgi?db=nucleotide&val=NM_005251.2) | FKHL14\|LD\|MFH-1\|MFH1 | forkhead box C2 (MFH-1, mesenchyme forkhead 1) |
| FOXO4 | [NM_005938.2](http://www.ncbi.nlm.nih.gov/entrez/viewer.fcgi?db=nucleotide&val=NM_005938.2) | AFX\|AFX1\|MLLT7 | forkhead box O4 |
| FRAS1 | [NM_001166133.1](http://www.ncbi.nlm.nih.gov/entrez/viewer.fcgi?db=nucleotide&val=NM_001166133.1) | - | Fraser syndrome 1 |
| FREM1 | [NM_001177704.1](http://www.ncbi.nlm.nih.gov/entrez/viewer.fcgi?db=nucleotide&val=NM_001177704.1) | BNAR\|C9orf143\|C9orf145\|C9orf154\|MOTA\|TILRR\|TRIGNO2 | FRAS1 related extracellular matrix 1 |
| FREM2 | [NM_207361.4](http://www.ncbi.nlm.nih.gov/entrez/viewer.fcgi?db=nucleotide&val=NM_207361.4) | - | FRAS1 related extracellular matrix protein 2 |
| FST | [NM_006350.2](http://www.ncbi.nlm.nih.gov/entrez/viewer.fcgi?db=nucleotide&val=NM_006350.2) | FS | follistatin |
| FSTL1 | [NM_007085.4](http://www.ncbi.nlm.nih.gov/entrez/viewer.fcgi?db=nucleotide&val=NM_007085.4) | FRP\|FSL1 | follistatin-like 1 |
| FUT3 | [NM_000149.3](http://www.ncbi.nlm.nih.gov/entrez/viewer.fcgi?db=nucleotide&val=NM_000149.3) | CD174\|FT3B\|FucT-III\|LE\|Les | fucosyltransferase 3 (galactoside 3(4)-L-fucosyltransferase, Lewis blood group) |
| FXYD6 | [NM_001164831.1](http://www.ncbi.nlm.nih.gov/entrez/viewer.fcgi?db=nucleotide&val=NM_001164831.1) | - | FXYD domain containing ion transport regulator 6 |
| GALNT7 | [NM_017423.2](http://www.ncbi.nlm.nih.gov/entrez/viewer.fcgi?db=nucleotide&val=NM_017423.2) | GALNAC-T7\|GalNAcT7 | UDP-N-acetyl-alpha-D-galactosamine:polypeptide N-acetylgalactosaminyltransferase 7 (GalNAc-T7) |
| GATA4 | [NM_002052.3](http://www.ncbi.nlm.nih.gov/entrez/viewer.fcgi?db=nucleotide&val=NM_002052.3) | ASD2\|VSD1 | GATA binding protein 4 |
| GDF15 | [NM_004864.2](http://www.ncbi.nlm.nih.gov/entrez/viewer.fcgi?db=nucleotide&val=NM_004864.2) | GDF-15\|MIC-1\|MIC1\|NAG-1\|PDF\|PLAB\|PTGFB | growth differentiation factor 15 |
| GDF5 | [NM_000557.2](http://www.ncbi.nlm.nih.gov/entrez/viewer.fcgi?db=nucleotide&val=NM_000557.2) | BMP14\|CDMP1\|LAP4\|OS5\|SYNS2 | growth differentiation factor 5 |
| GDF6 | [NM_001001557.2](http://www.ncbi.nlm.nih.gov/entrez/viewer.fcgi?db=nucleotide&val=NM_001001557.2) | BMP13\|CDMP2\|KFM\|KFS\|KFS1\|KFSL\|MCOP4\|MCOPCB6\|SCDO4\|SGM1 | growth differentiation factor 6 |
| GIMAP4 | [NM_018326.2](http://www.ncbi.nlm.nih.gov/entrez/viewer.fcgi?db=nucleotide&val=NM_018326.2) | IAN-1\|IAN1\|IMAP4 | GTPase, IMAP family member 4 |
| GIMAP6 | [NR_024115.1](http://www.ncbi.nlm.nih.gov/entrez/viewer.fcgi?db=nucleotide&val=NR_024115.1) | IAN-2\|IAN-6\|IAN2\|IAN6 | GTPase, IMAP family member 6 |
| GJA5 | [NM_005266.5](http://www.ncbi.nlm.nih.gov/entrez/viewer.fcgi?db=nucleotide&val=NM_005266.5) | ATFB11\|CX40 | gap junction protein, alpha 5, 40kDa |
| GLYR1 | [NM_032569.3](http://www.ncbi.nlm.nih.gov/entrez/viewer.fcgi?db=nucleotide&val=NM_032569.3) | BM045\|HIBDL\|N-PAC\|NP60 | glyoxylate reductase 1 homolog (Arabidopsis) |
| GPI | [NM_000175.2](http://www.ncbi.nlm.nih.gov/entrez/viewer.fcgi?db=nucleotide&val=NM_000175.2) | AMF\|GNPI\|NLK\|PGI\|PHI\|SA-36\|SA36 | glucose-6-phosphate isomerase |
| GPR124 | [NM_032777.9](http://www.ncbi.nlm.nih.gov/entrez/viewer.fcgi?db=nucleotide&val=NM_032777.9) | TEM5 | G protein-coupled receptor 124 |
| GPR56 | [NM_005682.4](http://www.ncbi.nlm.nih.gov/entrez/viewer.fcgi?db=nucleotide&val=NM_005682.4) | BFPP\|TM7LN4\|TM7XN1 | G protein-coupled receptor 56 |
| GPX1 | [NM_000581.2](http://www.ncbi.nlm.nih.gov/entrez/viewer.fcgi?db=nucleotide&val=NM_000581.2) | GPXD\|GSHPX1 | glutathione peroxidase 1 |
| GREM1 | [NM_013372.5](http://www.ncbi.nlm.nih.gov/entrez/viewer.fcgi?db=nucleotide&val=NM_013372.5) | CKTSF1B1\|DAND2\|DRM\|GREMLIN\|IHG-2 | gremlin 1 |
| GRHL2 | [NM_024915.3](http://www.ncbi.nlm.nih.gov/entrez/viewer.fcgi?db=nucleotide&val=NM_024915.3) | BOM\|DFNA28\|TFCP2L3 | grainyhead-like 2 (Drosophila) |
| GSN | [NM_000177.4](http://www.ncbi.nlm.nih.gov/entrez/viewer.fcgi?db=nucleotide&val=NM_000177.4) | ADF\|AGEL | gelsolin |
| GTF2I | [NM_033001.2](http://www.ncbi.nlm.nih.gov/entrez/viewer.fcgi?db=nucleotide&val=NM_033001.2) | BAP135\|BTKAP1\|DIWS\|GTFII-I\|IB291\|SPIN\|TFII-I\|WBS\|WBSCR6 | general transcription factor IIi |
| GZMK | [NM_002104.2](http://www.ncbi.nlm.nih.gov/entrez/viewer.fcgi?db=nucleotide&val=NM_002104.2) | TRYP2 | granzyme K (granzyme 3; tryptase II) |
| HAPLN1 | [NM_001884.3](http://www.ncbi.nlm.nih.gov/entrez/viewer.fcgi?db=nucleotide&val=NM_001884.3) | CRTL1 | hyaluronan and proteoglycan link protein 1 |
| HAS1 | [NM_001523.2](http://www.ncbi.nlm.nih.gov/entrez/viewer.fcgi?db=nucleotide&val=NM_001523.2) | HAS | hyaluronan synthase 1 |
| HDAC5 | [NM_005474.4](http://www.ncbi.nlm.nih.gov/entrez/viewer.fcgi?db=nucleotide&val=NM_005474.4) | HD5\|NY-CO-9 | histone deacetylase 5 |
| HDHD3 | [NM_031219.2](http://www.ncbi.nlm.nih.gov/entrez/viewer.fcgi?db=nucleotide&val=NM_031219.2) | 2810435D12Rik\|C9orf158 | haloacid dehalogenase-like hydrolase domain containing 3 |
| HEG1 | [NM_020733.1](http://www.ncbi.nlm.nih.gov/entrez/viewer.fcgi?db=nucleotide&val=NM_020733.1) | HEG\|MST112\|MSTP112 | HEG homolog 1 (zebrafish) |
| HGF | [NM_000601.4](http://www.ncbi.nlm.nih.gov/entrez/viewer.fcgi?db=nucleotide&val=NM_000601.4) | DFNB39\|F-TCF\|HGFB\|HPTA\|SF | hepatocyte growth factor (hepapoietin A; scatter factor) |
| HIF1A | [NM_001530.2](http://www.ncbi.nlm.nih.gov/entrez/viewer.fcgi?db=nucleotide&val=NM_001530.2) | HIF-1A\|HIF-1alpha\|HIF1\|HIF1-ALPHA\|MOP1\|PASD8\|bHLHe78 | hypoxia inducible factor 1, alpha subunit (basic helix-loop-helix transcription factor) |
| HIPK1 | [NM_152696.3](http://www.ncbi.nlm.nih.gov/entrez/viewer.fcgi?db=nucleotide&val=NM_152696.3) | Myak\|Nbak2 | homeodomain interacting protein kinase 1 |
| HIPK2 | [NM_022740.4](http://www.ncbi.nlm.nih.gov/entrez/viewer.fcgi?db=nucleotide&val=NM_022740.4) | PRO0593 | homeodomain interacting protein kinase 2 |
| HK2 | [NM_000189.4](http://www.ncbi.nlm.nih.gov/entrez/viewer.fcgi?db=nucleotide&val=NM_000189.4) | HKII\|HXK2 | hexokinase 2 |
| HK3 | [NM_002115.1](http://www.ncbi.nlm.nih.gov/entrez/viewer.fcgi?db=nucleotide&val=NM_002115.1) | HKIII\|HXK3 | hexokinase 3 (white cell) |
| HKDC1 | [NM_025130.3](http://www.ncbi.nlm.nih.gov/entrez/viewer.fcgi?db=nucleotide&val=NM_025130.3) | - | hexokinase domain containing 1 |
| HLA-DPB1 | [NM_002121.4](http://www.ncbi.nlm.nih.gov/entrez/viewer.fcgi?db=nucleotide&val=NM_002121.4) | DPB1\|HLA-DP\|HLA-DP1B\|HLA-DPB | major histocompatibility complex, class II, DP beta 1 |
| HMOX1 | [NM_002133.2](http://www.ncbi.nlm.nih.gov/entrez/viewer.fcgi?db=nucleotide&val=NM_002133.2) | HO-1\|HSP32\|bK286B10 | heme oxygenase (decycling) 1 |
| HOXA5 | [NM_019102.2](http://www.ncbi.nlm.nih.gov/entrez/viewer.fcgi?db=nucleotide&val=NM_019102.2) | HOX1\|HOX1.3\|HOX1C | homeobox A5 |
| HOXA7 | [NM_006896.3](http://www.ncbi.nlm.nih.gov/entrez/viewer.fcgi?db=nucleotide&val=NM_006896.3) | ANTP\|HOX1\|HOX1.1\|HOX1A | homeobox A7 |
| HOXB13 | [NM_006361.5](http://www.ncbi.nlm.nih.gov/entrez/viewer.fcgi?db=nucleotide&val=NM_006361.5) | PSGD | homeobox B13 |
| HOXB3 | [NM_002146.4](http://www.ncbi.nlm.nih.gov/entrez/viewer.fcgi?db=nucleotide&val=NM_002146.4) | HOX2\|HOX2G\|Hox-2.7 | homeobox B3 |
| HPSE | [NM_006665.3](http://www.ncbi.nlm.nih.gov/entrez/viewer.fcgi?db=nucleotide&val=NM_006665.3) | HPA\|HPA1\|HPR1\|HPSE1\|HSE1 | heparanase |
| HRAS | [NM_005343.2](http://www.ncbi.nlm.nih.gov/entrez/viewer.fcgi?db=nucleotide&val=NM_005343.2) | C-BAS/HAS\|C-H-RAS\|C-HA-RAS1\|CTLO\|H-RASIDX\|HAMSV\|HRAS1\|K-RAS\|N-RAS\|RASH1 | v-Ha-ras Harvey rat sarcoma viral oncogene homolog |
| HSD17B12 | [NM_016142.2](http://www.ncbi.nlm.nih.gov/entrez/viewer.fcgi?db=nucleotide&val=NM_016142.2) | KAR\|SDR12C1 | hydroxysteroid (17-beta) dehydrogenase 12 |
| HSP90B1 | [NM_003299.1](http://www.ncbi.nlm.nih.gov/entrez/viewer.fcgi?db=nucleotide&val=NM_003299.1) | ECGP\|GP96\|GRP94\|TRA1 | heat shock protein 90kDa beta (Grp94), member 1 |
| HSPB1 | [NM_001540.3](http://www.ncbi.nlm.nih.gov/entrez/viewer.fcgi?db=nucleotide&val=NM_001540.3) | CMT2F\|HMN2B\|HS.76067\|HSP27\|HSP28\|Hsp25\|SRP27 | heat shock 27kDa protein 1 |
| HSPG2 | [NM_005529.5](http://www.ncbi.nlm.nih.gov/entrez/viewer.fcgi?db=nucleotide&val=NM_005529.5) | HSPG\|PLC\|PRCAN\|SJA\|SJS\|SJS1 | heparan sulfate proteoglycan 2 |
| HUNK | [NM_014586.1](http://www.ncbi.nlm.nih.gov/entrez/viewer.fcgi?db=nucleotide&val=NM_014586.1) | - | hormonally up-regulated Neu-associated kinase |
| IBSP | [NM_004967.3](http://www.ncbi.nlm.nih.gov/entrez/viewer.fcgi?db=nucleotide&val=NM_004967.3) | BNSP\|BSP\|BSP-II\|SP-II | integrin-binding sialoprotein |
| ICAM1 | [NM_000201.2](http://www.ncbi.nlm.nih.gov/entrez/viewer.fcgi?db=nucleotide&val=NM_000201.2) | BB2\|CD54\|P3.58 | intercellular adhesion molecule 1 |
| ID1 | [NM_002165.2](http://www.ncbi.nlm.nih.gov/entrez/viewer.fcgi?db=nucleotide&val=NM_002165.2) | ID\|bHLHb24 | inhibitor of DNA binding 1, dominant negative helix-loop-helix protein |
| ID2 | [NM_002166.4](http://www.ncbi.nlm.nih.gov/entrez/viewer.fcgi?db=nucleotide&val=NM_002166.4) | GIG8\|ID2A\|ID2H\|bHLHb26 | inhibitor of DNA binding 2, dominant negative helix-loop-helix protein |
| ID4 | [NM_001546.2](http://www.ncbi.nlm.nih.gov/entrez/viewer.fcgi?db=nucleotide&val=NM_001546.2) | IDB4\|bHLHb27 | inhibitor of DNA binding 4, dominant negative helix-loop-helix protein |
| IFNG | [NM_000619.2](http://www.ncbi.nlm.nih.gov/entrez/viewer.fcgi?db=nucleotide&val=NM_000619.2) | IFG\|IFI | interferon, gamma |
| IGF1 | [NM_000618.3](http://www.ncbi.nlm.nih.gov/entrez/viewer.fcgi?db=nucleotide&val=NM_000618.3) | IGF-I\|IGF1A\|IGFI | insulin-like growth factor 1 (somatomedin C) |
| IGFBP4 | [NM_001552.2](http://www.ncbi.nlm.nih.gov/entrez/viewer.fcgi?db=nucleotide&val=NM_001552.2) | BP-4\|HT29-IGFBP\|IBP4\|IGFBP-4 | insulin-like growth factor binding protein 4 |
| IGFBP7 | [NM_001553.1](http://www.ncbi.nlm.nih.gov/entrez/viewer.fcgi?db=nucleotide&val=NM_001553.1) | AGM\|FSTL2\|IBP-7\|IGFBP-7\|IGFBP-7v\|IGFBPRP1\|MAC25\|PSF\|RAMSVPS\|TAF | insulin-like growth factor binding protein 7 |
| IL10RA | [NM_001558.2](http://www.ncbi.nlm.nih.gov/entrez/viewer.fcgi?db=nucleotide&val=NM_001558.2) | CD210\|CD210a\|CDW210A\|HIL-10R\|IL-10R1\|IL10R | interleukin 10 receptor, alpha |
| IL11 | [NM_000641.2](http://www.ncbi.nlm.nih.gov/entrez/viewer.fcgi?db=nucleotide&val=NM_000641.2) | AGIF\|IL-11 | interleukin 11 |
| IL13RA2 | [NM_000640.2](http://www.ncbi.nlm.nih.gov/entrez/viewer.fcgi?db=nucleotide&val=NM_000640.2) | CD213A2\|CT19\|IL-13R\|IL13BP | interleukin 13 receptor, alpha 2 |
| IL15 | [NM_172174.1](http://www.ncbi.nlm.nih.gov/entrez/viewer.fcgi?db=nucleotide&val=NM_172174.1) | IL-15 | interleukin 15 |
| IL18 | [NM_001562.2](http://www.ncbi.nlm.nih.gov/entrez/viewer.fcgi?db=nucleotide&val=NM_001562.2) | IGIF\|IL-18\|IL-1g\|IL1F4 | interleukin 18 (interferon-gamma-inducing factor) |
| IL1A | [NM_000575.3](http://www.ncbi.nlm.nih.gov/entrez/viewer.fcgi?db=nucleotide&val=NM_000575.3) | IL-1A\|IL1\|IL1-ALPHA\|IL1F1 | interleukin 1, alpha |
| IL1B | [NM_000576.2](http://www.ncbi.nlm.nih.gov/entrez/viewer.fcgi?db=nucleotide&val=NM_000576.2) | IL-1\|IL1-BETA\|IL1F2 | interleukin 1, beta |
| IL1RL1 | [NM_016232.4](http://www.ncbi.nlm.nih.gov/entrez/viewer.fcgi?db=nucleotide&val=NM_016232.4) | DER4\|FIT-1\|IL33R\|ST2\|ST2L\|ST2V\|T1 | interleukin 1 receptor-like 1 |
| IL1RN | [NM_000577.3](http://www.ncbi.nlm.nih.gov/entrez/viewer.fcgi?db=nucleotide&val=NM_000577.3) | DIRA\|ICIL-1RA\|IL-1RN\|IL-1ra\|IL-1ra3\|IL1F3\|IL1RA\|IRAP\|MVCD4 | interleukin 1 receptor antagonist |
| IL6 | [NM_000600.1](http://www.ncbi.nlm.nih.gov/entrez/viewer.fcgi?db=nucleotide&val=NM_000600.1) | BSF2\|HGF\|HSF\|IFNB2\|IL-6 | interleukin 6 (interferon, beta 2) |
| ILK | [NM_004517.2](http://www.ncbi.nlm.nih.gov/entrez/viewer.fcgi?db=nucleotide&val=NM_004517.2) | ILK-2\|P59 | integrin-linked kinase |
| INHBA | [NM_002192.2](http://www.ncbi.nlm.nih.gov/entrez/viewer.fcgi?db=nucleotide&val=NM_002192.2) | EDF\|FRP | inhibin, beta A |
| INHBE | [NM_031479.3](http://www.ncbi.nlm.nih.gov/entrez/viewer.fcgi?db=nucleotide&val=NM_031479.3) | - | inhibin, beta E |
| IRF6 | [NM_006147.2](http://www.ncbi.nlm.nih.gov/entrez/viewer.fcgi?db=nucleotide&val=NM_006147.2) | LPS\|OFC6\|PIT\|PPS\|VWS\|VWS1 | interferon regulatory factor 6 |
| ISL1 | [NM_002202.2](http://www.ncbi.nlm.nih.gov/entrez/viewer.fcgi?db=nucleotide&val=NM_002202.2) | ISLET1\|Isl-1 | ISL LIM homeobox 1 |
| ISLR | [NM_005545.3](http://www.ncbi.nlm.nih.gov/entrez/viewer.fcgi?db=nucleotide&val=NM_005545.3) | HsT17563 | immunoglobulin superfamily containing leucine-rich repeat |
| ITGA1 | [NM_181501.1](http://www.ncbi.nlm.nih.gov/entrez/viewer.fcgi?db=nucleotide&val=NM_181501.1) | CD49a\|VLA1 | integrin, alpha 1 |
| ITGA11 | [NM_012211.3](http://www.ncbi.nlm.nih.gov/entrez/viewer.fcgi?db=nucleotide&val=NM_012211.3) | HsT18964 | integrin, alpha 11 |
| ITGA2 | [NM_002203.2](http://www.ncbi.nlm.nih.gov/entrez/viewer.fcgi?db=nucleotide&val=NM_002203.2) | BDPLT9\|BR\|CD49B\|GPIa\|HPA-5\|VLA-2\|VLAA2 | integrin, alpha 2 (CD49B, alpha 2 subunit of VLA-2 receptor) |
| ITGA3 | [NM_002204.2](http://www.ncbi.nlm.nih.gov/entrez/viewer.fcgi?db=nucleotide&val=NM_002204.2) | CD49C\|GAP-B3\|GAPB3\|MSK18\|VCA-2\|VL3A\|VLA3a | integrin, alpha 3 (antigen CD49C, alpha 3 subunit of VLA-3 receptor) |
| ITGA5 | [NM_002205.2](http://www.ncbi.nlm.nih.gov/entrez/viewer.fcgi?db=nucleotide&val=NM_002205.2) | CD49e\|FNRA\|VLA5A | integrin, alpha 5 (fibronectin receptor, alpha polypeptide) |
| ITGA6 | [NM_000210.1](http://www.ncbi.nlm.nih.gov/entrez/viewer.fcgi?db=nucleotide&val=NM_000210.1) | CD49f\|ITGA6B\|VLA-6 | integrin, alpha 6 |
| ITGA7 | [NM_002206.1](http://www.ncbi.nlm.nih.gov/entrez/viewer.fcgi?db=nucleotide&val=NM_002206.1) | - | integrin, alpha 7 |
| ITGA8 | [NM_003638.1](http://www.ncbi.nlm.nih.gov/entrez/viewer.fcgi?db=nucleotide&val=NM_003638.1) | - | integrin, alpha 8 |
| ITGA9 | [NM_002207.2](http://www.ncbi.nlm.nih.gov/entrez/viewer.fcgi?db=nucleotide&val=NM_002207.2) | ALPHA-RLC\|ITGA4L\|RLC | integrin, alpha 9 |
| ITGAM | [NM_000632.3](http://www.ncbi.nlm.nih.gov/entrez/viewer.fcgi?db=nucleotide&val=NM_000632.3) | CD11B\|CR3A\|MAC-1\|MAC1A\|MO1A\|SLEB6 | integrin, alpha M (complement component 3 receptor 3 subunit) |
| ITGB1 | [NM_033666.2](http://www.ncbi.nlm.nih.gov/entrez/viewer.fcgi?db=nucleotide&val=NM_033666.2) | CD29\|FNRB\|GPIIA\|MDF2\|MSK12\|VLA-BETA\|VLAB | integrin, beta 1 (fibronectin receptor, beta polypeptide, antigen CD29 includes MDF2, MSK12) |
| ITGB1BP1 | [NM_004763.3](http://www.ncbi.nlm.nih.gov/entrez/viewer.fcgi?db=nucleotide&val=NM_004763.3) | ICAP-1A\|ICAP-1B\|ICAP-1alpha\|ICAP1\|ICAP1A\|ICAP1B | integrin beta 1 binding protein 1 |
| ITGB2 | [NM_000211.2](http://www.ncbi.nlm.nih.gov/entrez/viewer.fcgi?db=nucleotide&val=NM_000211.2) | CD18\|LAD\|LCAMB\|LFA-1\|MAC-1\|MF17\|MFI7 | integrin, beta 2 (complement component 3 receptor 3 and 4 subunit) |
| ITGB3 | [NM_000212.2](http://www.ncbi.nlm.nih.gov/entrez/viewer.fcgi?db=nucleotide&val=NM_000212.2) | BDPLT2\|CD61\|GP3A\|GPIIIa\|GT | integrin, beta 3 (platelet glycoprotein IIIa, antigen CD61) |
| ITGB4 | [NM_001005731.1](http://www.ncbi.nlm.nih.gov/entrez/viewer.fcgi?db=nucleotide&val=NM_001005731.1) | CD104 | integrin, beta 4 |
| ITGB6 | [NM_000888.3](http://www.ncbi.nlm.nih.gov/entrez/viewer.fcgi?db=nucleotide&val=NM_000888.3) | - | integrin, beta 6 |
| ITGB7 | [NM_000889.1](http://www.ncbi.nlm.nih.gov/entrez/viewer.fcgi?db=nucleotide&val=NM_000889.1) | - | integrin, beta 7 |
| ITGB8 | [NM_002214.2](http://www.ncbi.nlm.nih.gov/entrez/viewer.fcgi?db=nucleotide&val=NM_002214.2) | - | integrin, beta 8 |
| ITM2A | [NM_004867.4](http://www.ncbi.nlm.nih.gov/entrez/viewer.fcgi?db=nucleotide&val=NM_004867.4) | BRICD2A\|E25A | integral membrane protein 2A |
| JAG1 | [NM_000214.2](http://www.ncbi.nlm.nih.gov/entrez/viewer.fcgi?db=nucleotide&val=NM_000214.2) | AGS\|AHD\|AWS\|CD339\|HJ1\|JAGL1 | jagged 1 |
| JAM2 | [NM_001270407.1](http://www.ncbi.nlm.nih.gov/entrez/viewer.fcgi?db=nucleotide&val=NM_001270407.1) | C21orf43\|CD322\|JAM-B\|JAMB\|PRO245\|VE-JAM\|VEJAM | junctional adhesion molecule 2 |
| JAM3 | [NM_032801.3](http://www.ncbi.nlm.nih.gov/entrez/viewer.fcgi?db=nucleotide&val=NM_032801.3) | JAM-2\|JAM-3\|JAM-C\|JAMC | junctional adhesion molecule 3 |
| JUN | [NM_002228.3](http://www.ncbi.nlm.nih.gov/entrez/viewer.fcgi?db=nucleotide&val=NM_002228.3) | AP-1\|AP1\|c-Jun | jun proto-oncogene |
| KCNJ8 | [NM_004982.2](http://www.ncbi.nlm.nih.gov/entrez/viewer.fcgi?db=nucleotide&val=NM_004982.2) | KIR6.1\|uKATP-1 | potassium inwardly-rectifying channel, subfamily J, member 8 |
| KDM1A | [NM_015013.3](http://www.ncbi.nlm.nih.gov/entrez/viewer.fcgi?db=nucleotide&val=NM_015013.3) | AOF2\|BHC110\|KDM1\|LSD1 | lysine (K)-specific demethylase 1A |
| KDR | [NM_002253.2](http://www.ncbi.nlm.nih.gov/entrez/viewer.fcgi?db=nucleotide&val=NM_002253.2) | CD309\|FLK1\|VEGFR\|VEGFR2 | kinase insert domain receptor (a type III receptor tyrosine kinase) |
| KIAA1462 | [NM_020848.2](http://www.ncbi.nlm.nih.gov/entrez/viewer.fcgi?db=nucleotide&val=NM_020848.2) | JCAD | KIAA1462 |
| KISS1 | [NM_002256.3](http://www.ncbi.nlm.nih.gov/entrez/viewer.fcgi?db=nucleotide&val=NM_002256.3) | KiSS-1 | KiSS-1 metastasis-suppressor |
| KLK3 | [NM_001030049.1](http://www.ncbi.nlm.nih.gov/entrez/viewer.fcgi?db=nucleotide&val=NM_001030049.1) | APS\|KLK2A1\|PSA\|hK3 | kallikrein-related peptidase 3 |
| KRAS | [NM_004985.3](http://www.ncbi.nlm.nih.gov/entrez/viewer.fcgi?db=nucleotide&val=NM_004985.3) | C-K-RAS\|K-RAS2A\|K-RAS2B\|K-RAS4A\|K-RAS4B\|KI-RAS\|KRAS1\|KRAS2\|NS\|NS3\|RASK2 | v-Ki-ras2 Kirsten rat sarcoma viral oncogene homolog |
| KRIT1 | [NM_004912.3](http://www.ncbi.nlm.nih.gov/entrez/viewer.fcgi?db=nucleotide&val=NM_004912.3) | CAM\|CCM1 | KRIT1, ankyrin repeat containing |
| KRT1 | [NM_006121.2](http://www.ncbi.nlm.nih.gov/entrez/viewer.fcgi?db=nucleotide&val=NM_006121.2) | CK1\|EHK\|EHK1\|EPPK\|K1\|KRT1A\|NEPPK | keratin 1 |
| KRT14 | [NM_000526.4](http://www.ncbi.nlm.nih.gov/entrez/viewer.fcgi?db=nucleotide&val=NM_000526.4) | CK14\|EBS3\|EBS4\|K14\|NFJ | keratin 14 |
| KRT19 | [NM_002276.4](http://www.ncbi.nlm.nih.gov/entrez/viewer.fcgi?db=nucleotide&val=NM_002276.4) | CK19\|K19\|K1CS | keratin 19 |
| KRT7 | [NM_005556.3](http://www.ncbi.nlm.nih.gov/entrez/viewer.fcgi?db=nucleotide&val=NM_005556.3) | CK7\|K2C7\|K7\|SCL | keratin 7 |
| LAD1 | [NM_005558.3](http://www.ncbi.nlm.nih.gov/entrez/viewer.fcgi?db=nucleotide&val=NM_005558.3) | LadA | ladinin 1 |
| LAMA1 | [NM_005559.2](http://www.ncbi.nlm.nih.gov/entrez/viewer.fcgi?db=nucleotide&val=NM_005559.2) | LAMA\|S-LAM-alpha | laminin, alpha 1 |
| LAMA3 | [NM_000227.3](http://www.ncbi.nlm.nih.gov/entrez/viewer.fcgi?db=nucleotide&val=NM_000227.3) | BM600\|E170\|LAMNA\|LOCS\|lama3a | laminin, alpha 3 |
| LAMA4 | [NM_001105209.1](http://www.ncbi.nlm.nih.gov/entrez/viewer.fcgi?db=nucleotide&val=NM_001105209.1) | LAMA3\|LAMA4*-1 | laminin, alpha 4 |
| LAMA5 | [NM_005560.3](http://www.ncbi.nlm.nih.gov/entrez/viewer.fcgi?db=nucleotide&val=NM_005560.3) | - | laminin, alpha 5 |
| LAMB3 | [NM_000228.2](http://www.ncbi.nlm.nih.gov/entrez/viewer.fcgi?db=nucleotide&val=NM_000228.2) | BM600-125KDA\|LAM5\|LAMNB1 | laminin, beta 3 |
| LAMC1 | [NM_002293.3](http://www.ncbi.nlm.nih.gov/entrez/viewer.fcgi?db=nucleotide&val=NM_002293.3) | LAMB2 | laminin, gamma 1 (formerly LAMB2) |
| LAMC2 | [NM_005562.2](http://www.ncbi.nlm.nih.gov/entrez/viewer.fcgi?db=nucleotide&val=NM_005562.2) | B2T\|BM600\|CSF\|EBR2\|EBR2A\|LAMB2T\|LAMNB2 | laminin, gamma 2 |
| LDHA | [NM_001165414.1](http://www.ncbi.nlm.nih.gov/entrez/viewer.fcgi?db=nucleotide&val=NM_001165414.1) | GSD11\|LDH1\|LDHM | lactate dehydrogenase A |
| LEFTY1 | [NM_020997.2](http://www.ncbi.nlm.nih.gov/entrez/viewer.fcgi?db=nucleotide&val=NM_020997.2) | LEFTB\|LEFTYB | left-right determination factor 1 |
| LGALS1 | [NM_002305.3](http://www.ncbi.nlm.nih.gov/entrez/viewer.fcgi?db=nucleotide&val=NM_002305.3) | GAL1\|GBP | lectin, galactoside-binding, soluble, 1 |
| LHFP | [NM_005780.2](http://www.ncbi.nlm.nih.gov/entrez/viewer.fcgi?db=nucleotide&val=NM_005780.2) | - | lipoma HMGIC fusion partner |
| LIFR | [NM_002310.3](http://www.ncbi.nlm.nih.gov/entrez/viewer.fcgi?db=nucleotide&val=NM_002310.3) | CD118\|LIF-R\|SJS2\|STWS\|SWS | leukemia inhibitory factor receptor alpha |
| LLGL2 | [NM_001015002.1](http://www.ncbi.nlm.nih.gov/entrez/viewer.fcgi?db=nucleotide&val=NM_001015002.1) | HGL\|LGL2 | lethal giant larvae homolog 2 (Drosophila) |
| LOX | [NM_002317.4](http://www.ncbi.nlm.nih.gov/entrez/viewer.fcgi?db=nucleotide&val=NM_002317.4) | - | lysyl oxidase |
| LOXL2 | [NM_002318.2](http://www.ncbi.nlm.nih.gov/entrez/viewer.fcgi?db=nucleotide&val=NM_002318.2) | LOR2\|WS9-14 | lysyl oxidase-like 2 |
| LRG1 | [NM_052972.2](http://www.ncbi.nlm.nih.gov/entrez/viewer.fcgi?db=nucleotide&val=NM_052972.2) | HMFT1766\|LRG | leucine-rich alpha-2-glycoprotein 1 |
| LTBP4 | [NM_003573.2](http://www.ncbi.nlm.nih.gov/entrez/viewer.fcgi?db=nucleotide&val=NM_003573.2) | LTBP-4\|LTBP4L\|LTBP4S | latent transforming growth factor beta binding protein 4 |
| LUM | [NM_002345.3](http://www.ncbi.nlm.nih.gov/entrez/viewer.fcgi?db=nucleotide&val=NM_002345.3) | LDC\|SLRR2D | lumican |
| LY96 | [NM_015364.2](http://www.ncbi.nlm.nih.gov/entrez/viewer.fcgi?db=nucleotide&val=NM_015364.2) | ESOP-1\|MD-2\|MD2\|ly-96 | lymphocyte antigen 96 |
| MAF | [NM_005360.4](http://www.ncbi.nlm.nih.gov/entrez/viewer.fcgi?db=nucleotide&val=NM_005360.4) | CCA4\|c-MAF | v-maf musculoaponeurotic fibrosarcoma oncogene homolog (avian) |
| MAP2K1 | [NM_002755.2](http://www.ncbi.nlm.nih.gov/entrez/viewer.fcgi?db=nucleotide&val=NM_002755.2) | MAPKK1\|MEK1\|MKK1\|PRKMK1 | mitogen-activated protein kinase kinase 1 |
| MAP2K2 | [NM_030662.3](http://www.ncbi.nlm.nih.gov/entrez/viewer.fcgi?db=nucleotide&val=NM_030662.3) | MAPKK2\|MEK2\|MKK2\|PRKMK2 | mitogen-activated protein kinase kinase 2 |
| MAP2K4 | [NM_003010.2](http://www.ncbi.nlm.nih.gov/entrez/viewer.fcgi?db=nucleotide&val=NM_003010.2) | JNKK\|JNKK1\|MAPKK4\|MEK4\|MKK4\|PRKMK4\|SAPKK-1\|SAPKK1\|SEK1\|SERK1 | mitogen-activated protein kinase kinase 4 |
| MAP3K7 | [NM_145333.1](http://www.ncbi.nlm.nih.gov/entrez/viewer.fcgi?db=nucleotide&val=NM_145333.1) | MEKK7\|TAK1\|TGF1a | mitogen-activated protein kinase kinase kinase 7 |
| MAPK1 | [NM_138957.2](http://www.ncbi.nlm.nih.gov/entrez/viewer.fcgi?db=nucleotide&val=NM_138957.2) | ERK\|ERK2\|ERT1\|MAPK2\|P42MAPK\|PRKM1\|PRKM2\|p38\|p40\|p41\|p41mapk | mitogen-activated protein kinase 1 |
| MAPK3 | [NM_001040056.1](http://www.ncbi.nlm.nih.gov/entrez/viewer.fcgi?db=nucleotide&val=NM_001040056.1) | ERK-1\|ERK1\|ERT2\|HS44KDAP\|HUMKER1A\|P44ERK1\|P44MAPK\|PRKM3\|p44-ERK1\|p44-MAPK | mitogen-activated protein kinase 3 |
| MAPKAPK3 | [NM_004635.3](http://www.ncbi.nlm.nih.gov/entrez/viewer.fcgi?db=nucleotide&val=NM_004635.3) | 3PK\|MAPKAP-K3\|MAPKAP3\|MAPKAPK-3\|MK-3 | mitogen-activated protein kinase-activated protein kinase 3 |
| MCAM | [NM_006500.2](http://www.ncbi.nlm.nih.gov/entrez/viewer.fcgi?db=nucleotide&val=NM_006500.2) | CD146\|MUC18 | melanoma cell adhesion molecule |
| MED1 | [NM_004774.3](http://www.ncbi.nlm.nih.gov/entrez/viewer.fcgi?db=nucleotide&val=NM_004774.3) | CRSP1\|CRSP200\|DRIP205\|DRIP230\|PBP\|PPARBP\|PPARGBP\|RB18A\|TRAP220\|TRIP2 | mediator complex subunit 1 |
| MED23 | [NM_004830.2](http://www.ncbi.nlm.nih.gov/entrez/viewer.fcgi?db=nucleotide&val=NM_004830.2) | ARC130\|CRSP130\|CRSP133\|CRSP3\|DRIP130\|MRT18\|SUR-2\|SUR2 | mediator complex subunit 23 |
| MEG3 | [NR_002766.2](http://www.ncbi.nlm.nih.gov/entrez/viewer.fcgi?db=nucleotide&val=NR_002766.2) | FP504\|GTL2\|LINC00023\|NCRNA00023\|PRO0518\|PRO2160\|prebp1 | maternally expressed 3 (non-protein coding) |
| MEOX2 | [NM_005924.4](http://www.ncbi.nlm.nih.gov/entrez/viewer.fcgi?db=nucleotide&val=NM_005924.4) | GAX\|MOX2 | mesenchyme homeobox 2 |
| MET | [NM_001127500.1](http://www.ncbi.nlm.nih.gov/entrez/viewer.fcgi?db=nucleotide&val=NM_001127500.1) | AUTS9\|HGFR\|RCCP2\|c-Met | met proto-oncogene (hepatocyte growth factor receptor) |
| MFAP4 | [NM_002404.1](http://www.ncbi.nlm.nih.gov/entrez/viewer.fcgi?db=nucleotide&val=NM_002404.1) | - | microfibrillar-associated protein 4 |
| MGAT5 | [NM_002410.4](http://www.ncbi.nlm.nih.gov/entrez/viewer.fcgi?db=nucleotide&val=NM_002410.4) | GNT-V\|GNT-VA | mannosyl (alpha-1,6-)-glycoprotein beta-1,6-N-acetyl-glucosaminyltransferase |
| MGP | [NM_000900.2](http://www.ncbi.nlm.nih.gov/entrez/viewer.fcgi?db=nucleotide&val=NM_000900.2) | MGLAP\|NTI | matrix Gla protein |
| MISP | [NM_173481.2](http://www.ncbi.nlm.nih.gov/entrez/viewer.fcgi?db=nucleotide&val=NM_173481.2) | #N/A | #N/A |
| MMP1 | [NM_002421.2](http://www.ncbi.nlm.nih.gov/entrez/viewer.fcgi?db=nucleotide&val=NM_002421.2) | CLG\|CLGN | matrix metallopeptidase 1 (interstitial collagenase) |
| MMP10 | [NM_002425.1](http://www.ncbi.nlm.nih.gov/entrez/viewer.fcgi?db=nucleotide&val=NM_002425.1) | SL-2\|STMY2 | matrix metallopeptidase 10 (stromelysin 2) |
| MMP12 | [NM_002426.3](http://www.ncbi.nlm.nih.gov/entrez/viewer.fcgi?db=nucleotide&val=NM_002426.3) | HME\|ME\|MME\|MMP-12 | matrix metallopeptidase 12 (macrophage elastase) |
| MMP13 | [NM_002427.2](http://www.ncbi.nlm.nih.gov/entrez/viewer.fcgi?db=nucleotide&val=NM_002427.2) | CLG3\|MANDP1 | matrix metallopeptidase 13 (collagenase 3) |
| MMP14 | [NM_004995.2](http://www.ncbi.nlm.nih.gov/entrez/viewer.fcgi?db=nucleotide&val=NM_004995.2) | 1\|MMP-14\|MMP-X1\|MT-MMP\|MT-MMP 1\|MT1-MMP\|MT1MMP\|MTMMP1 | matrix metallopeptidase 14 (membrane-inserted) |
| MMP17 | [NM_016155.4](http://www.ncbi.nlm.nih.gov/entrez/viewer.fcgi?db=nucleotide&val=NM_016155.4) | MT4-MMP | matrix metallopeptidase 17 (membrane-inserted) |
| MMP2 | [NM_004530.2](http://www.ncbi.nlm.nih.gov/entrez/viewer.fcgi?db=nucleotide&val=NM_004530.2) | CLG4\|CLG4A\|MMP-II\|MONA\|TBE-1 | matrix metallopeptidase 2 (gelatinase A, 72kDa gelatinase, 72kDa type IV collagenase) |
| MMP24 | [NM_006690.3](http://www.ncbi.nlm.nih.gov/entrez/viewer.fcgi?db=nucleotide&val=NM_006690.3) | MMP25\|MT-MMP5\|MT5-MMP | matrix metallopeptidase 24 (membrane-inserted) |
| MMP3 | [NM_002422.3](http://www.ncbi.nlm.nih.gov/entrez/viewer.fcgi?db=nucleotide&val=NM_002422.3) | CHDS6\|MMP-3\|SL-1\|STMY\|STMY1\|STR1 | matrix metallopeptidase 3 (stromelysin 1, progelatinase) |
| MMP9 | [NM_004994.2](http://www.ncbi.nlm.nih.gov/entrez/viewer.fcgi?db=nucleotide&val=NM_004994.2) | CLG4B\|GELB\|MANDP2\|MMP-9 | matrix metallopeptidase 9 (gelatinase B, 92kDa gelatinase, 92kDa type IV collagenase) |
| MMRN2 | [NM_024756.2](http://www.ncbi.nlm.nih.gov/entrez/viewer.fcgi?db=nucleotide&val=NM_024756.2) | EMILIN-3\|EMILIN3\|EndoGlyx-1 | multimerin 2 |
| MPDZ | [NM_003829.4](http://www.ncbi.nlm.nih.gov/entrez/viewer.fcgi?db=nucleotide&val=NM_003829.4) | MUPP1 | multiple PDZ domain protein |
| MRC1 | [NM_002438.2](http://www.ncbi.nlm.nih.gov/entrez/viewer.fcgi?db=nucleotide&val=NM_002438.2) | CD206\|CLEC13D\|CLEC13DL\|MMR\|MRC1L1\|bA541I19.1 | mannose receptor, C type 1 |
| MS4A4A | [NM_024021.2](http://www.ncbi.nlm.nih.gov/entrez/viewer.fcgi?db=nucleotide&val=NM_024021.2) | 4SPAN1\|CD20-L1\|CD20L1\|MS4A4\|MS4A7 | membrane-spanning 4-domains, subfamily A, member 4A |
| MS4A6A | [NM_152852.2](http://www.ncbi.nlm.nih.gov/entrez/viewer.fcgi?db=nucleotide&val=NM_152852.2) | 4SPAN3\|4SPAN3.2\|CD20L3\|MS4A6\|MST090\|MSTP090 | membrane-spanning 4-domains, subfamily A, member 6A |
| MT3 | [NM_005954.2](http://www.ncbi.nlm.nih.gov/entrez/viewer.fcgi?db=nucleotide&val=NM_005954.2) | GIF\|GIFB\|GRIF\|ZnMT3 | metallothionein 3 |
| MTA1 | [NM_004689.2](http://www.ncbi.nlm.nih.gov/entrez/viewer.fcgi?db=nucleotide&val=NM_004689.2) | - | metastasis associated 1 |
| MTBP | [NM_022045.4](http://www.ncbi.nlm.nih.gov/entrez/viewer.fcgi?db=nucleotide&val=NM_022045.4) | MDM2BP | Mdm2, transformed 3T3 cell double minute 2, p53 binding protein (mouse) binding protein, 104kDa |
| MTDH | [NM_178812.3](http://www.ncbi.nlm.nih.gov/entrez/viewer.fcgi?db=nucleotide&val=NM_178812.3) | 3D3\|AEG-1\|AEG1\|LYRIC\|LYRIC/3D3 | metadherin |
| MTOR | [NM_004958.2](http://www.ncbi.nlm.nih.gov/entrez/viewer.fcgi?db=nucleotide&val=NM_004958.2) | FRAP\|FRAP1\|FRAP2\|RAFT1\|RAPT1 | mechanistic target of rapamycin (serine/threonine kinase) |
| MUC1 | [NM_001018017.1](http://www.ncbi.nlm.nih.gov/entrez/viewer.fcgi?db=nucleotide&val=NM_001018017.1) | CD227\|EMA\|H23AG\|KL-6\|MAM6\|MUC-1\|MUC-1/SEC\|MUC-1/X\|MUC1/ZD\|PEM\|PEMT\|PUM | mucin 1, cell surface associated |
| MYC | [NM_002467.3](http://www.ncbi.nlm.nih.gov/entrez/viewer.fcgi?db=nucleotide&val=NM_002467.3) | MRTL\|bHLHe39\|c-Myc | v-myc myelocytomatosis viral oncogene homolog (avian) |
| MYCL | [NM_001033081.2](http://www.ncbi.nlm.nih.gov/entrez/viewer.fcgi?db=nucleotide&val=NM_001033081.2) | #N/A | #N/A |
| MYH11 | [NM_001040113.1](http://www.ncbi.nlm.nih.gov/entrez/viewer.fcgi?db=nucleotide&val=NM_001040113.1) | AAT4\|FAA4\|SMHC\|SMMHC | myosin, heavy chain 11, smooth muscle |
| MYLK | [NM_053032.2](http://www.ncbi.nlm.nih.gov/entrez/viewer.fcgi?db=nucleotide&val=NM_053032.2) | AAT7\|KRP\|MLCK\|MLCK1\|MLCK108\|MLCK210\|MSTP083\|MYLK1\|smMLCK | myosin light chain kinase |
| MYO1D | [NM_015194.1](http://www.ncbi.nlm.nih.gov/entrez/viewer.fcgi?db=nucleotide&val=NM_015194.1) | myr4 | myosin ID |
| MYO5C | [NM_018728.2](http://www.ncbi.nlm.nih.gov/entrez/viewer.fcgi?db=nucleotide&val=NM_018728.2) | - | myosin VC |
| NAA15 | [NM_057175.3](http://www.ncbi.nlm.nih.gov/entrez/viewer.fcgi?db=nucleotide&val=NM_057175.3) | Ga19\|NARG1\|NATH\|TBDN100 | N(alpha)-acetyltransferase 15, NatA auxiliary subunit |
| NAP1L3 | [NM_004538.4](http://www.ncbi.nlm.nih.gov/entrez/viewer.fcgi?db=nucleotide&val=NM_004538.4) | MB20\|NPL3 | nucleosome assembly protein 1-like 3 |
| NCAM1 | [NM_000615.5](http://www.ncbi.nlm.nih.gov/entrez/viewer.fcgi?db=nucleotide&val=NM_000615.5) | CD56\|MSK39\|NCAM | neural cell adhesion molecule 1 |
| NCL | [NM_005381.2](http://www.ncbi.nlm.nih.gov/entrez/viewer.fcgi?db=nucleotide&val=NM_005381.2) | C23 | nucleolin |
| NDNF | [NM_024574.3](http://www.ncbi.nlm.nih.gov/entrez/viewer.fcgi?db=nucleotide&val=NM_024574.3) | C4orf31 | neuron-derived neurotrophic factor |
| NDP | [NM_000266.2](http://www.ncbi.nlm.nih.gov/entrez/viewer.fcgi?db=nucleotide&val=NM_000266.2) | EVR2\|FEVR\|ND | Norrie disease (pseudoglioma) |
| NDRG1 | [NM_006096.2](http://www.ncbi.nlm.nih.gov/entrez/viewer.fcgi?db=nucleotide&val=NM_006096.2) | CAP43\|CMT4D\|DRG-1\|DRG1\|GC4\|HMSNL\|NDR1\|NMSL\|PROXY1\|RIT42\|RTP\|TARG1\|TDD5 | N-myc downstream regulated 1 |
| NF1 | [NM_000267.2](http://www.ncbi.nlm.nih.gov/entrez/viewer.fcgi?db=nucleotide&val=NM_000267.2) | NFNS\|VRNF\|WSS | neurofibromin 1 |
| NF2 | [NM_181825.2](http://www.ncbi.nlm.nih.gov/entrez/viewer.fcgi?db=nucleotide&val=NM_181825.2) | ACN\|BANF\|SCH | neurofibromin 2 (merlin) |
| NFAT5 | [NM_173214.1](http://www.ncbi.nlm.nih.gov/entrez/viewer.fcgi?db=nucleotide&val=NM_173214.1) | NF-AT5\|NFATL1\|NFATZ\|OREBP\|TONEBP | nuclear factor of activated T-cells 5, tonicity-responsive |
| NFATC2 | [NM_012340.3](http://www.ncbi.nlm.nih.gov/entrez/viewer.fcgi?db=nucleotide&val=NM_012340.3) | NFAT1\|NFATP | nuclear factor of activated T-cells, cytoplasmic, calcineurin-dependent 2 |
| NFKB1 | [NM_003998.2](http://www.ncbi.nlm.nih.gov/entrez/viewer.fcgi?db=nucleotide&val=NM_003998.2) | EBP-1\|KBF1\|NF-kB1\|NF-kappa-B\|NF-kappaB\|NFKB-p105\|NFKB-p50\|NFkappaB\|p105\|p50 | nuclear factor of kappa light polypeptide gene enhancer in B-cells 1 |
| NID2 | [NM_007361.3](http://www.ncbi.nlm.nih.gov/entrez/viewer.fcgi?db=nucleotide&val=NM_007361.3) | NID-2 | nidogen 2 (osteonidogen) |
| NME1 | [NM_000269.2](http://www.ncbi.nlm.nih.gov/entrez/viewer.fcgi?db=nucleotide&val=NM_000269.2) | AWD\|GAAD\|NB\|NBS\|NDKA\|NDPK-A\|NDPKA\|NM23\|NM23-H1 | NME/NM23 nucleoside diphosphate kinase 1 |
| NME4 | [NM_005009.2](http://www.ncbi.nlm.nih.gov/entrez/viewer.fcgi?db=nucleotide&val=NM_005009.2) | NDPK-D\|NM23H4\|nm23-H4 | NME/NM23 nucleoside diphosphate kinase 4 |
| NODAL | [NM_018055.3](http://www.ncbi.nlm.nih.gov/entrez/viewer.fcgi?db=nucleotide&val=NM_018055.3) | HTX5 | nodal homolog (mouse) |
| NOS2 | [NM_153292.1](http://www.ncbi.nlm.nih.gov/entrez/viewer.fcgi?db=nucleotide&val=NM_153292.1) | HEP-NOS\|INOS\|NOS\|NOS2A | nitric oxide synthase 2, inducible |
| NOS3 | [NM_000603.4](http://www.ncbi.nlm.nih.gov/entrez/viewer.fcgi?db=nucleotide&val=NM_000603.4) | ECNOS\|eNOS | nitric oxide synthase 3 (endothelial cell) |
| NOTCH1 | [NM_017617.3](http://www.ncbi.nlm.nih.gov/entrez/viewer.fcgi?db=nucleotide&val=NM_017617.3) | TAN1\|hN1 | notch 1 |
| NOX5 | [NM_024505.2](http://www.ncbi.nlm.nih.gov/entrez/viewer.fcgi?db=nucleotide&val=NM_024505.2) | - | NADPH oxidase, EF-hand calcium binding domain 5 |
| NPR1 | [NM_000906.2](http://www.ncbi.nlm.nih.gov/entrez/viewer.fcgi?db=nucleotide&val=NM_000906.2) | ANPRA\|ANPa\|GUC2A\|GUCY2A\|NPRA | natriuretic peptide receptor A/guanylate cyclase A (atrionatriuretic peptide receptor A) |
| NR3C1 | [NM_001018077.1](http://www.ncbi.nlm.nih.gov/entrez/viewer.fcgi?db=nucleotide&val=NM_001018077.1) | GCCR\|GCR\|GR\|GRL | nuclear receptor subfamily 3, group C, member 1 (glucocorticoid receptor) |
| NR4A1 | [NM_173157.1](http://www.ncbi.nlm.nih.gov/entrez/viewer.fcgi?db=nucleotide&val=NM_173157.1) | GFRP1\|HMR\|N10\|NAK-1\|NGFIB\|NP10\|NUR77\|TR3 | nuclear receptor subfamily 4, group A, member 1 |
| NR4A3 | [NM_173198.1](http://www.ncbi.nlm.nih.gov/entrez/viewer.fcgi?db=nucleotide&val=NM_173198.1) | CHN\|CSMF\|MINOR\|NOR1\|TEC | nuclear receptor subfamily 4, group A, member 3 |
| NRCAM | [NM_005010.4](http://www.ncbi.nlm.nih.gov/entrez/viewer.fcgi?db=nucleotide&val=NM_005010.4) | - | neuronal cell adhesion molecule |
| NRP1 | [NM_003873.5](http://www.ncbi.nlm.nih.gov/entrez/viewer.fcgi?db=nucleotide&val=NM_003873.5) | BDCA4\|CD304\|NP1\|NRP\|VEGF165R | neuropilin 1 |
| NRP2 | [NM_003872.2](http://www.ncbi.nlm.nih.gov/entrez/viewer.fcgi?db=nucleotide&val=NM_003872.2) | NP2\|NPN2\|PRO2714\|VEGF165R2 | neuropilin 2 |
| NRXN1 | [NM_138735.2](http://www.ncbi.nlm.nih.gov/entrez/viewer.fcgi?db=nucleotide&val=NM_138735.2) | Hs.22998\|PTHSL2\|SCZD17 | neurexin 1 |
| NRXN3 | [NM_001105250.1](http://www.ncbi.nlm.nih.gov/entrez/viewer.fcgi?db=nucleotide&val=NM_001105250.1) | C14orf60 | neurexin 3 |
| NTRK1 | [NM_001012331.1](http://www.ncbi.nlm.nih.gov/entrez/viewer.fcgi?db=nucleotide&val=NM_001012331.1) | MTC\|TRK\|TRK1\|TRKA\|Trk-A\|p140-TrkA | neurotrophic tyrosine kinase, receptor, type 1 |
| OAS1 | [NM_001032409.1](http://www.ncbi.nlm.nih.gov/entrez/viewer.fcgi?db=nucleotide&val=NM_001032409.1) | IFI-4\|OIAS\|OIASI | 2'-5'-oligoadenylate synthetase 1, 40/46kDa |
| OCLN | [NM_002538.3](http://www.ncbi.nlm.nih.gov/entrez/viewer.fcgi?db=nucleotide&val=NM_002538.3) | BLCPMG | occludin |
| OGN | [NM_014057.3](http://www.ncbi.nlm.nih.gov/entrez/viewer.fcgi?db=nucleotide&val=NM_014057.3) | OG\|OIF\|SLRR3A | osteoglycin |
| OLFML2B | [NM_015441.1](http://www.ncbi.nlm.nih.gov/entrez/viewer.fcgi?db=nucleotide&val=NM_015441.1) | RP11-227F8.1 | olfactomedin-like 2B |
| OVOL2 | [NM_021220.2](http://www.ncbi.nlm.nih.gov/entrez/viewer.fcgi?db=nucleotide&val=NM_021220.2) | EUROIMAGE566589\|ZNF339 | ovo-like 2 (Drosophila) |
| P3H1 | [NM_001146289.1](http://www.ncbi.nlm.nih.gov/entrez/viewer.fcgi?db=nucleotide&val=NM_001146289.1) | #N/A | #N/A |
| P3H2 | [NM_018192.2](http://www.ncbi.nlm.nih.gov/entrez/viewer.fcgi?db=nucleotide&val=NM_018192.2) | #N/A | #N/A |
| PCOLCE | [NM_002593.3](http://www.ncbi.nlm.nih.gov/entrez/viewer.fcgi?db=nucleotide&val=NM_002593.3) | PCPE\|PCPE-1\|PCPE1 | procollagen C-endopeptidase enhancer |
| PDCD10 | [NM_145859.1](http://www.ncbi.nlm.nih.gov/entrez/viewer.fcgi?db=nucleotide&val=NM_145859.1) | CCM3\|TFAR15 | programmed cell death 10 |
| PDCL3 | [NM_024065.4](http://www.ncbi.nlm.nih.gov/entrez/viewer.fcgi?db=nucleotide&val=NM_024065.4) | HTPHLP\|PHLP2A\|PHLP3\|VIAF\|VIAF1 | phosducin-like 3 |
| PDGFA | [NM_002607.5](http://www.ncbi.nlm.nih.gov/entrez/viewer.fcgi?db=nucleotide&val=NM_002607.5) | PDGF-A\|PDGF1 | platelet-derived growth factor alpha polypeptide |
| PDGFC | [NM_016205.2](http://www.ncbi.nlm.nih.gov/entrez/viewer.fcgi?db=nucleotide&val=NM_016205.2) | FALLOTEIN\|SCDGF | platelet derived growth factor C |
| PDGFRB | [NM_002609.3](http://www.ncbi.nlm.nih.gov/entrez/viewer.fcgi?db=nucleotide&val=NM_002609.3) | CD140B\|JTK12\|PDGFR\|PDGFR-1\|PDGFR1 | platelet-derived growth factor receptor, beta polypeptide |
| PDK1 | [NM_002610.3](http://www.ncbi.nlm.nih.gov/entrez/viewer.fcgi?db=nucleotide&val=NM_002610.3) | - | pyruvate dehydrogenase kinase, isozyme 1 |
| PDPN | [NM_006474.4](http://www.ncbi.nlm.nih.gov/entrez/viewer.fcgi?db=nucleotide&val=NM_006474.4) | AGGRUS\|GP36\|GP40\|Gp38\|HT1A-1\|OTS8\|PA2.26\|T1A\|T1A-2 | podoplanin |
| PEBP4 | [NM_144962.2](http://www.ncbi.nlm.nih.gov/entrez/viewer.fcgi?db=nucleotide&val=NM_144962.2) | CORK-1\|CORK1\|GWTM1933\|PEBP-4\|PRO4408\|hPEBP4 | phosphatidylethanolamine-binding protein 4 |
| PECAM1 | [NM_000442.3](http://www.ncbi.nlm.nih.gov/entrez/viewer.fcgi?db=nucleotide&val=NM_000442.3) | CD31\|CD31/EndoCAM\|GPIIA'\|PECA1\|PECAM-1\|endoCAM | platelet/endothelial cell adhesion molecule 1 |
| PFKFB1 | [NM_002625.2](http://www.ncbi.nlm.nih.gov/entrez/viewer.fcgi?db=nucleotide&val=NM_002625.2) | F6PK\|HL2K\|PFRX | 6-phosphofructo-2-kinase/fructose-2,6-biphosphatase 1 |
| PFKFB4 | [NM_004567.2](http://www.ncbi.nlm.nih.gov/entrez/viewer.fcgi?db=nucleotide&val=NM_004567.2) | - | 6-phosphofructo-2-kinase/fructose-2,6-biphosphatase 4 |
| PGK1 | [NM_000291.2](http://www.ncbi.nlm.nih.gov/entrez/viewer.fcgi?db=nucleotide&val=NM_000291.2) | MIG10\|PGKA | phosphoglycerate kinase 1 |
| PIK3CA | [NM_006218.2](http://www.ncbi.nlm.nih.gov/entrez/viewer.fcgi?db=nucleotide&val=NM_006218.2) | CLOVE\|PI3K\|p110-alpha | phosphatidylinositol-4,5-bisphosphate 3-kinase, catalytic subunit alpha |
| PIK3CD | [NM_005026.3](http://www.ncbi.nlm.nih.gov/entrez/viewer.fcgi?db=nucleotide&val=NM_005026.3) | P110DELTA\|PI3K\|p110D | phosphatidylinositol-4,5-bisphosphate 3-kinase, catalytic subunit delta |
| PIK3CG | [NM_002649.2](http://www.ncbi.nlm.nih.gov/entrez/viewer.fcgi?db=nucleotide&val=NM_002649.2) | PI3CG\|PI3K\|PI3Kgamma\|PIK3 | phosphatidylinositol-4,5-bisphosphate 3-kinase, catalytic subunit gamma |
| PIK3R1 | [NM_181504.2](http://www.ncbi.nlm.nih.gov/entrez/viewer.fcgi?db=nucleotide&val=NM_181504.2) | GRB1\|p85\|p85-ALPHA | phosphoinositide-3-kinase, regulatory subunit 1 (alpha) |
| PIK3R2 | [NM_005027.2](http://www.ncbi.nlm.nih.gov/entrez/viewer.fcgi?db=nucleotide&val=NM_005027.2) | P85B\|p85\|p85-BETA | phosphoinositide-3-kinase, regulatory subunit 2 (beta) |
| PIK3R5 | [NM_001142633.1](http://www.ncbi.nlm.nih.gov/entrez/viewer.fcgi?db=nucleotide&val=NM_001142633.1) | F730038I15Rik\|FOAP-2\|P101-PI3K\|p101 | phosphoinositide-3-kinase, regulatory subunit 5 |
| PIK3R6 | [NM_001010855.3](http://www.ncbi.nlm.nih.gov/entrez/viewer.fcgi?db=nucleotide&val=NM_001010855.3) | C17orf38\|HsT41028\|p84\|p87(PIKAP)\|p87PIKAP | phosphoinositide-3-kinase, regulatory subunit 6 |
| PITX2 | [NM_000325.5](http://www.ncbi.nlm.nih.gov/entrez/viewer.fcgi?db=nucleotide&val=NM_000325.5) | ARP1\|Brx1\|IDG2\|IGDS\|IGDS2\|IHG2\|IRID2\|Otlx2\|PTX2\|RGS\|RIEG\|RIEG1\|RS | paired-like homeodomain 2 |
| PKM | [NM_182471.1](http://www.ncbi.nlm.nih.gov/entrez/viewer.fcgi?db=nucleotide&val=NM_182471.1) | CTHBP\|OIP3\|PK3\|PKM2\|TCB\|THBP1 | pyruvate kinase, muscle |
| PKN1 | [NM_213560.1](http://www.ncbi.nlm.nih.gov/entrez/viewer.fcgi?db=nucleotide&val=NM_213560.1) | DBK\|PAK-1\|PAK1\|PKN\|PKN-ALPHA\|PRK1\|PRKCL1 | protein kinase N1 |
| PKNOX1 | [NM_004571.3](http://www.ncbi.nlm.nih.gov/entrez/viewer.fcgi?db=nucleotide&val=NM_004571.3) | PREP1\|pkonx1c | PBX/knotted 1 homeobox 1 |
| PLA2G10 | [NM_003561.1](http://www.ncbi.nlm.nih.gov/entrez/viewer.fcgi?db=nucleotide&val=NM_003561.1) | GXPLA2\|GXSPLA2\|SPLA2 | phospholipase A2, group X |
| PLA2G2A | [NM_000300.2](http://www.ncbi.nlm.nih.gov/entrez/viewer.fcgi?db=nucleotide&val=NM_000300.2) | MOM1\|PLA2\|PLA2B\|PLA2L\|PLA2S\|PLAS1\|sPLA2 | phospholipase A2, group IIA (platelets, synovial fluid) |
| PLA2G2D | [NM_001271814.1](http://www.ncbi.nlm.nih.gov/entrez/viewer.fcgi?db=nucleotide&val=NM_001271814.1) | SPLASH\|sPLA2S | phospholipase A2, group IID |
| PLA2G3 | [NM_015715.3](http://www.ncbi.nlm.nih.gov/entrez/viewer.fcgi?db=nucleotide&val=NM_015715.3) | GIII-SPLA2\|SPLA2III | phospholipase A2, group III |
| PLAU | [NM_002658.2](http://www.ncbi.nlm.nih.gov/entrez/viewer.fcgi?db=nucleotide&val=NM_002658.2) | ATF\|BDPLT5\|QPD\|UPA\|URK\|u-PA | plasminogen activator, urokinase |
| PLAUR | [NM_001005376.1](http://www.ncbi.nlm.nih.gov/entrez/viewer.fcgi?db=nucleotide&val=NM_001005376.1) | CD87\|U-PAR\|UPAR\|URKR | plasminogen activator, urokinase receptor |
| PLCG1 | [NM_002660.2](http://www.ncbi.nlm.nih.gov/entrez/viewer.fcgi?db=nucleotide&val=NM_002660.2) | NCKAP3\|PLC-II\|PLC1\|PLC148\|PLCgamma1 | phospholipase C, gamma 1 |
| PLCG2 | [NM_002661.2](http://www.ncbi.nlm.nih.gov/entrez/viewer.fcgi?db=nucleotide&val=NM_002661.2) | FCAS3 | phospholipase C, gamma 2 (phosphatidylinositol-specific) |
| PLEKHO1 | [NM_016274.4](http://www.ncbi.nlm.nih.gov/entrez/viewer.fcgi?db=nucleotide&val=NM_016274.4) | CKIP-1\|OC120 | pleckstrin homology domain containing, family O member 1 |
| PLS1 | [NM_002670.2](http://www.ncbi.nlm.nih.gov/entrez/viewer.fcgi?db=nucleotide&val=NM_002670.2) | - | plastin 1 |
| PLXDC1 | [NM_020405.4](http://www.ncbi.nlm.nih.gov/entrez/viewer.fcgi?db=nucleotide&val=NM_020405.4) | TEM3\|TEM7 | plexin domain containing 1 |
| PLXNC1 | [NM_005761.2](http://www.ncbi.nlm.nih.gov/entrez/viewer.fcgi?db=nucleotide&val=NM_005761.2) | CD232\|PLXN-C1\|VESPR | plexin C1 |
| PLXND1 | [NM_015103.2](http://www.ncbi.nlm.nih.gov/entrez/viewer.fcgi?db=nucleotide&val=NM_015103.2) | PLEXD1 | plexin D1 |
| PMP22 | [NM_000304.2](http://www.ncbi.nlm.nih.gov/entrez/viewer.fcgi?db=nucleotide&val=NM_000304.2) | CMT1A\|CMT1E\|DSS\|GAS-3\|HMSNIA\|HNPP\|Sp110 | peripheral myelin protein 22 |
| PNPLA6 | [NM_006702.3](http://www.ncbi.nlm.nih.gov/entrez/viewer.fcgi?db=nucleotide&val=NM_006702.3) | NTE\|NTEMND\|SPG39\|iPLA2delta\|sws | patatin-like phospholipase domain containing 6 |
| POPDC3 | [NM_022361.4](http://www.ncbi.nlm.nih.gov/entrez/viewer.fcgi?db=nucleotide&val=NM_022361.4) | POP3\|RP11-99L11.2\|bA355M14.1 | popeye domain containing 3 |
| POSTN | [NM_001135935.1](http://www.ncbi.nlm.nih.gov/entrez/viewer.fcgi?db=nucleotide&val=NM_001135935.1) | OSF-2\|OSF2\|PDLPOSTN\|PN\|RP11-412K4.1\|periostin | periostin, osteoblast specific factor |
| PPFIBP2 | [NM_003621.2](http://www.ncbi.nlm.nih.gov/entrez/viewer.fcgi?db=nucleotide&val=NM_003621.2) | Cclp1 | PTPRF interacting protein, binding protein 2 (liprin beta 2) |
| PPL | [NM_002705.4](http://www.ncbi.nlm.nih.gov/entrez/viewer.fcgi?db=nucleotide&val=NM_002705.4) | - | periplakin |
| PPP1R16B | [NM_015568.2](http://www.ncbi.nlm.nih.gov/entrez/viewer.fcgi?db=nucleotide&val=NM_015568.2) | ANKRD4\|TIMAP | protein phosphatase 1, regulatory subunit 16B |
| PPP2CB | [NM_001009552.1](http://www.ncbi.nlm.nih.gov/entrez/viewer.fcgi?db=nucleotide&val=NM_001009552.1) | PP2Abeta\|PP2CB | protein phosphatase 2, catalytic subunit, beta isozyme |
| PPP2R1A | [NM_014225.3](http://www.ncbi.nlm.nih.gov/entrez/viewer.fcgi?db=nucleotide&val=NM_014225.3) | PP2A-Aalpha\|PP2AAALPHA\|PR65A | protein phosphatase 2, regulatory subunit A, alpha |
| PPP3R1 | [NM_000945.3](http://www.ncbi.nlm.nih.gov/entrez/viewer.fcgi?db=nucleotide&val=NM_000945.3) | CALNB1\|CNB\|CNB1 | protein phosphatase 3, regulatory subunit B, alpha |
| PRELP | [NM_002725.3](http://www.ncbi.nlm.nih.gov/entrez/viewer.fcgi?db=nucleotide&val=NM_002725.3) | MST161\|MSTP161\|SLRR2A | proline/arginine-rich end leucine-rich repeat protein |
| PRF1 | [NM_005041.3](http://www.ncbi.nlm.nih.gov/entrez/viewer.fcgi?db=nucleotide&val=NM_005041.3) | FLH2\|HPLH2\|P1\|PFN1\|PFP | perforin 1 (pore forming protein) |
| PRKCB | [NM_212535.1](http://www.ncbi.nlm.nih.gov/entrez/viewer.fcgi?db=nucleotide&val=NM_212535.1) | PKC-beta\|PKCB\|PRKCB1\|PRKCB2 | protein kinase C, beta |
| PRKCG | [NM_002739.3](http://www.ncbi.nlm.nih.gov/entrez/viewer.fcgi?db=nucleotide&val=NM_002739.3) | PKC-gamma\|PKCC\|PKCG\|SCA14 | protein kinase C, gamma |
| PRKCZ | [NM_002744.4](http://www.ncbi.nlm.nih.gov/entrez/viewer.fcgi?db=nucleotide&val=NM_002744.4) | PKC-ZETA\|PKC2 | protein kinase C, zeta |
| PROK2 | [NM_021935.3](http://www.ncbi.nlm.nih.gov/entrez/viewer.fcgi?db=nucleotide&val=NM_021935.3) | BV8\|KAL4\|MIT1\|PK2 | prokineticin 2 |
| PROM1 | [NM_006017.1](http://www.ncbi.nlm.nih.gov/entrez/viewer.fcgi?db=nucleotide&val=NM_006017.1) | AC133\|CD133\|CORD12\|MCDR2\|PROML1\|RP41\|STGD4 | prominin 1 |
| PRR15L | [NM_024320.2](http://www.ncbi.nlm.nih.gov/entrez/viewer.fcgi?db=nucleotide&val=NM_024320.2) | ATAD4 | proline rich 15-like |
| PRSS22 | [NM_022119.3](http://www.ncbi.nlm.nih.gov/entrez/viewer.fcgi?db=nucleotide&val=NM_022119.3) | BSSP-4\|hBSSP-4 | protease, serine, 22 |
| PRSS8 | [NM_002773.3](http://www.ncbi.nlm.nih.gov/entrez/viewer.fcgi?db=nucleotide&val=NM_002773.3) | CAP1\|PROSTASIN | protease, serine, 8 |
| PTEN | [NM_000314.4](http://www.ncbi.nlm.nih.gov/entrez/viewer.fcgi?db=nucleotide&val=NM_000314.4) | 10q23del\|BZS\|DEC\|GLM2\|MHAM\|MMAC1\|PTEN1\|TEP1 | phosphatase and tensin homolog |
| PTGDS | [NM_000954.5](http://www.ncbi.nlm.nih.gov/entrez/viewer.fcgi?db=nucleotide&val=NM_000954.5) | L-PGDS\|LPGDS\|PDS\|PGD2\|PGDS\|PGDS2 | prostaglandin D2 synthase 21kDa (brain) |
| PTGIS | [NM_000961.3](http://www.ncbi.nlm.nih.gov/entrez/viewer.fcgi?db=nucleotide&val=NM_000961.3) | CYP8\|CYP8A1\|PGIS\|PTGI | prostaglandin I2 (prostacyclin) synthase |
| PTGS2 | [NM_000963.1](http://www.ncbi.nlm.nih.gov/entrez/viewer.fcgi?db=nucleotide&val=NM_000963.1) | COX-2\|COX2\|GRIPGHS\|PGG/HS\|PGHS-2\|PHS-2\|hCox-2 | prostaglandin-endoperoxide synthase 2 (prostaglandin G/H synthase and cyclooxygenase) |
| PTK2 | [NM_153831.2](http://www.ncbi.nlm.nih.gov/entrez/viewer.fcgi?db=nucleotide&val=NM_153831.2) | FADK\|FAK\|FAK1\|FRNK\|PPP1R71\|p125FAK\|pp125FAK | PTK2 protein tyrosine kinase 2 |
| PTK2B | [NM_004103.3](http://www.ncbi.nlm.nih.gov/entrez/viewer.fcgi?db=nucleotide&val=NM_004103.3) | CADTK\|CAKB\|FADK2\|FAK2\|PKB\|PTK\|PYK2\|RAFTK | PTK2B protein tyrosine kinase 2 beta |
| PTK6 | [NM_005975.2](http://www.ncbi.nlm.nih.gov/entrez/viewer.fcgi?db=nucleotide&val=NM_005975.2) | BRK | PTK6 protein tyrosine kinase 6 |
| PTPRB | [NM_002837.3](http://www.ncbi.nlm.nih.gov/entrez/viewer.fcgi?db=nucleotide&val=NM_002837.3) | HPTP-BETA\|HPTPB\|PTPB\|R-PTP-BETA\|VEPTP | protein tyrosine phosphatase, receptor type, B |
| PTPRC | [NM_080923.2](http://www.ncbi.nlm.nih.gov/entrez/viewer.fcgi?db=nucleotide&val=NM_080923.2) | B220\|CD45\|CD45R\|GP180\|L-CA\|LCA\|LY5\|T200 | protein tyrosine phosphatase, receptor type, C |
| PTPRM | [NM_002845.3](http://www.ncbi.nlm.nih.gov/entrez/viewer.fcgi?db=nucleotide&val=NM_002845.3) | PTPRL1\|R-PTP-MU\|RPTPM\|RPTPU\|hR-PTPu | protein tyrosine phosphatase, receptor type, M |
| PTRF | [NM_012232.5](http://www.ncbi.nlm.nih.gov/entrez/viewer.fcgi?db=nucleotide&val=NM_012232.5) | CAVIN\|CAVIN1\|CGL4\|cavin-1 | polymerase I and transcript release factor |
| PTTG1 | [NM_004219.2](http://www.ncbi.nlm.nih.gov/entrez/viewer.fcgi?db=nucleotide&val=NM_004219.2) | EAP1\|HPTTG\|PTTG\|TUTR1 | pituitary tumor-transforming 1 |
| PTX3 | [NM_002852.3](http://www.ncbi.nlm.nih.gov/entrez/viewer.fcgi?db=nucleotide&val=NM_002852.3) | TNFAIP5\|TSG-14 | pentraxin 3, long |
| PXDN | [NM_012293.1](http://www.ncbi.nlm.nih.gov/entrez/viewer.fcgi?db=nucleotide&val=NM_012293.1) | D2S448\|D2S448E\|MG50\|PRG2\|PXN\|VPO | peroxidasin homolog (Drosophila) |
| PYCARD | [NM_013258.3](http://www.ncbi.nlm.nih.gov/entrez/viewer.fcgi?db=nucleotide&val=NM_013258.3) | ASC\|CARD5\|TMS\|TMS-1\|TMS1 | PYD and CARD domain containing |
| QKI | [NM_006775.2](http://www.ncbi.nlm.nih.gov/entrez/viewer.fcgi?db=nucleotide&val=NM_006775.2) | Hqk\|QK\|QK1\|QK3\|hqkI | QKI, KH domain containing, RNA binding |
| RAB25 | [NM_020387.2](http://www.ncbi.nlm.nih.gov/entrez/viewer.fcgi?db=nucleotide&val=NM_020387.2) | CATX-8\|RAB11C | RAB25, member RAS oncogene family |
| RAC1 | [NM_198829.1](http://www.ncbi.nlm.nih.gov/entrez/viewer.fcgi?db=nucleotide&val=NM_198829.1) | Rac-1\|TC-25\|p21-Rac1 | ras-related C3 botulinum toxin substrate 1 (rho family, small GTP binding protein Rac1) |
| RAC2 | [NM_002872.3](http://www.ncbi.nlm.nih.gov/entrez/viewer.fcgi?db=nucleotide&val=NM_002872.3) | EN-7\|Gx\|HSPC022 | ras-related C3 botulinum toxin substrate 2 (rho family, small GTP binding protein Rac2) |
| RAF1 | [NM_002880.3](http://www.ncbi.nlm.nih.gov/entrez/viewer.fcgi?db=nucleotide&val=NM_002880.3) | CRAF\|NS5\|Raf-1\|c-Raf | v-raf-1 murine leukemia viral oncogene homolog 1 |
| RAMP1 | [NM_005855.2](http://www.ncbi.nlm.nih.gov/entrez/viewer.fcgi?db=nucleotide&val=NM_005855.2) | - | receptor (G protein-coupled) activity modifying protein 1 |
| RAMP2 | [NM_005854.2](http://www.ncbi.nlm.nih.gov/entrez/viewer.fcgi?db=nucleotide&val=NM_005854.2) | - | receptor (G protein-coupled) activity modifying protein 2 |
| RB1 | [NM_000321.1](http://www.ncbi.nlm.nih.gov/entrez/viewer.fcgi?db=nucleotide&val=NM_000321.1) | OSRC\|RB\|p105-Rb\|pRb\|pp110 | retinoblastoma 1 |
| RBL1 | [NM_183404.1](http://www.ncbi.nlm.nih.gov/entrez/viewer.fcgi?db=nucleotide&val=NM_183404.1) | CP107\|PRB1\|p107 | retinoblastoma-like 1 (p107) |
| RBL2 | [NM_005611.3](http://www.ncbi.nlm.nih.gov/entrez/viewer.fcgi?db=nucleotide&val=NM_005611.3) | P130\|Rb2 | retinoblastoma-like 2 (p130) |
| RBM47 | [NM_019027.3](http://www.ncbi.nlm.nih.gov/entrez/viewer.fcgi?db=nucleotide&val=NM_019027.3) | NET18 | RNA binding motif protein 47 |
| RBPJ | [NM_015874.3](http://www.ncbi.nlm.nih.gov/entrez/viewer.fcgi?db=nucleotide&val=NM_015874.3) | CBF1\|IGKJRB\|IGKJRB1\|KBF2\|RBP-J\|RBPJK\|RBPSUH\|SUH\|csl | recombination signal binding protein for immunoglobulin kappa J region |
| RBX1 | [NM_014248.2](http://www.ncbi.nlm.nih.gov/entrez/viewer.fcgi?db=nucleotide&val=NM_014248.2) | BA554C12.1\|RNF75\|ROC1 | ring-box 1, E3 ubiquitin protein ligase |
| RELN | [NM_005045.2](http://www.ncbi.nlm.nih.gov/entrez/viewer.fcgi?db=nucleotide&val=NM_005045.2) | LIS2\|PRO1598\|RL | reelin |
| RGCC | [NM_014059.2](http://www.ncbi.nlm.nih.gov/entrez/viewer.fcgi?db=nucleotide&val=NM_014059.2) | C13orf15\|RGC-32\|RGC32\|bA157L14.2 | regulator of cell cycle |
| RHOA | [NM_001664.2](http://www.ncbi.nlm.nih.gov/entrez/viewer.fcgi?db=nucleotide&val=NM_001664.2) | ARH12\|ARHA\|RHO12\|RHOH12 | ras homolog family member A |
| RNH1 | [NM_203384.1](http://www.ncbi.nlm.nih.gov/entrez/viewer.fcgi?db=nucleotide&val=NM_203384.1) | RAI\|RNH | ribonuclease/angiogenin inhibitor 1 |
| ROBO4 | [NM_019055.5](http://www.ncbi.nlm.nih.gov/entrez/viewer.fcgi?db=nucleotide&val=NM_019055.5) | ECSM4\|MRB | roundabout, axon guidance receptor, homolog 4 (Drosophila) |
| ROCK1 | [NM_005406.1](http://www.ncbi.nlm.nih.gov/entrez/viewer.fcgi?db=nucleotide&val=NM_005406.1) | P160ROCK\|ROCK-I | Rho-associated, coiled-coil containing protein kinase 1 |
| ROCK2 | [NM_004850.3](http://www.ncbi.nlm.nih.gov/entrez/viewer.fcgi?db=nucleotide&val=NM_004850.3) | ROCK-II | Rho-associated, coiled-coil containing protein kinase 2 |
| RORA | [NM_134261.2](http://www.ncbi.nlm.nih.gov/entrez/viewer.fcgi?db=nucleotide&val=NM_134261.2) | NR1F1\|ROR1\|ROR2\|ROR3\|RZR-ALPHA\|RZRA | RAR-related orphan receptor A |
| RORB | [NM_006914.3](http://www.ncbi.nlm.nih.gov/entrez/viewer.fcgi?db=nucleotide&val=NM_006914.3) | NR1F2\|ROR-BETA\|RZR-BETA\|RZRB\|bA133M9.1 | RAR-related orphan receptor B |
| RPS27A | [NM_002954.5](http://www.ncbi.nlm.nih.gov/entrez/viewer.fcgi?db=nucleotide&val=NM_002954.5) | CEP80\|S27A\|UBA80\|UBC\|UBCEP1\|UBCEP80 | ribosomal protein S27a |
| RPS6KB1 | [NM_003161.2](http://www.ncbi.nlm.nih.gov/entrez/viewer.fcgi?db=nucleotide&val=NM_003161.2) | PS6K\|S6K\|S6K1\|STK14A\|p70(S6K)-alpha\|p70-S6K\|p70-alpha | ribosomal protein S6 kinase, 70kDa, polypeptide 1 |
| RPS6KB2 | [NM_003952.2](http://www.ncbi.nlm.nih.gov/entrez/viewer.fcgi?db=nucleotide&val=NM_003952.2) | KLS\|P70-beta\|P70-beta-1\|P70-beta-2\|S6K-beta2\|S6K2\|SRK\|STK14B\|p70(S6K)-beta\|p70S6Kb | ribosomal protein S6 kinase, 70kDa, polypeptide 2 |
| RRAS | [NM_006270.3](http://www.ncbi.nlm.nih.gov/entrez/viewer.fcgi?db=nucleotide&val=NM_006270.3) | - | related RAS viral (r-ras) oncogene homolog |
| RTN4 | [NM_007008.2](http://www.ncbi.nlm.nih.gov/entrez/viewer.fcgi?db=nucleotide&val=NM_007008.2) | ASY\|NI220/250\|NOGO\|NOGO-A\|NOGOC\|NSP\|NSP-CL\|Nbla00271\|Nbla10545\|Nogo-B\|Nogo-C\|RTN-X\|RTN4-A\|RTN4-B1\|RTN4-B2\|RTN4-C | reticulon 4 |
| RUNX1 | [NM_001754.4](http://www.ncbi.nlm.nih.gov/entrez/viewer.fcgi?db=nucleotide&val=NM_001754.4) | AML1\|AML1-EVI-1\|AMLCR1\|CBFA2\|EVI-1\|PEBP2aB | runt-related transcription factor 1 |
| RUNX1T1 | [NM_004349.2](http://www.ncbi.nlm.nih.gov/entrez/viewer.fcgi?db=nucleotide&val=NM_004349.2) | AML1T1\|CBFA2T1\|CDR\|ETO\|MTG8\|ZMYND2 | runt-related transcription factor 1; translocated to, 1 (cyclin D-related) |
| S100A14 | [NM_020672.1](http://www.ncbi.nlm.nih.gov/entrez/viewer.fcgi?db=nucleotide&val=NM_020672.1) | BCMP84\|S100A15 | S100 calcium binding protein A14 |
| S100A7 | [NM_002963.2](http://www.ncbi.nlm.nih.gov/entrez/viewer.fcgi?db=nucleotide&val=NM_002963.2) | PSOR1\|S100A7c | S100 calcium binding protein A7 |
| S1PR1 | [NM_001400.4](http://www.ncbi.nlm.nih.gov/entrez/viewer.fcgi?db=nucleotide&val=NM_001400.4) | CD363\|CHEDG1\|D1S3362\|ECGF1\|EDG-1\|EDG1\|S1P1 | sphingosine-1-phosphate receptor 1 |
| SACS | [NM_014363.4](http://www.ncbi.nlm.nih.gov/entrez/viewer.fcgi?db=nucleotide&val=NM_014363.4) | ARSACS\|DNAJC29 | spastic ataxia of Charlevoix-Saguenay (sacsin) |
| SAMSN1 | [NM_022136.3](http://www.ncbi.nlm.nih.gov/entrez/viewer.fcgi?db=nucleotide&val=NM_022136.3) | HACS1\|NASH1\|SASH2\|SH3D6B\|SLy2 | SAM domain, SH3 domain and nuclear localization signals 1 |
| SCG2 | [NM_003469.3](http://www.ncbi.nlm.nih.gov/entrez/viewer.fcgi?db=nucleotide&val=NM_003469.3) | CHGC\|EM66\|SN\|SgII | secretogranin II |
| SCNN1A | [NM_001038.4](http://www.ncbi.nlm.nih.gov/entrez/viewer.fcgi?db=nucleotide&val=NM_001038.4) | BESC2\|ENaCa\|ENaCalpha\|SCNEA\|SCNN1 | sodium channel, non-voltage-gated 1 alpha subunit |
| SDC4 | [NM_002999.2](http://www.ncbi.nlm.nih.gov/entrez/viewer.fcgi?db=nucleotide&val=NM_002999.2) | SYND4 | syndecan 4 |
| SELE | [NM_000450.2](http://www.ncbi.nlm.nih.gov/entrez/viewer.fcgi?db=nucleotide&val=NM_000450.2) | CD62E\|ELAM\|ELAM1\|ESEL\|LECAM2 | selectin E |
| SEMA3E | [NM_012431.1](http://www.ncbi.nlm.nih.gov/entrez/viewer.fcgi?db=nucleotide&val=NM_012431.1) | M-SEMAH\|M-SemaK\|SEMAH\|coll-5 | sema domain, immunoglobulin domain (Ig), short basic domain, secreted, (semaphorin) 3E |
| SERINC5 | [NM_001174071.1](http://www.ncbi.nlm.nih.gov/entrez/viewer.fcgi?db=nucleotide&val=NM_001174071.1) | C5orf12\|TPO1 | serine incorporator 5 |
| SERPINA1 | [NM_000295.4](http://www.ncbi.nlm.nih.gov/entrez/viewer.fcgi?db=nucleotide&val=NM_000295.4) | A1A\|A1AT\|AAT\|PI\|PI1\|PRO2275\|alpha1AT | serpin peptidase inhibitor, clade A (alpha-1 antiproteinase, antitrypsin), member 1 |
| SERPINE1 | [NM_001165413.1](http://www.ncbi.nlm.nih.gov/entrez/viewer.fcgi?db=nucleotide&val=NM_001165413.1) | PAI\|PAI-1\|PAI1\|PLANH1 | serpin peptidase inhibitor, clade E (nexin, plasminogen activator inhibitor type 1), member 1 |
| SERPINF1 | [NM_002615.4](http://www.ncbi.nlm.nih.gov/entrez/viewer.fcgi?db=nucleotide&val=NM_002615.4) | EPC-1\|OI12\|OI6\|PEDF | serpin peptidase inhibitor, clade F (alpha-2 antiplasmin, pigment epithelium derived factor), member 1 |
| SERPING1 | [NM_000062.2](http://www.ncbi.nlm.nih.gov/entrez/viewer.fcgi?db=nucleotide&val=NM_000062.2) | C1IN\|C1INH\|C1NH\|HAE1\|HAE2 | serpin peptidase inhibitor, clade G (C1 inhibitor), member 1 |
| SERPINH1 | [NM_001235.2](http://www.ncbi.nlm.nih.gov/entrez/viewer.fcgi?db=nucleotide&val=NM_001235.2) | AsTP3\|CBP1\|CBP2\|HSP47\|OI10\|PPROM\|RA-A47\|SERPINH2\|gp46 | serpin peptidase inhibitor, clade H (heat shock protein 47), member 1, (collagen binding protein 1) |
| SET | [NM_001122821.1](http://www.ncbi.nlm.nih.gov/entrez/viewer.fcgi?db=nucleotide&val=NM_001122821.1) | 2PP2A\|I2PP2A\|IGAAD\|IPP2A2\|PHAPII\|TAF-I\|TAF-IBETA | SET nuclear oncogene |
| SETD2 | [NM_014159.6](http://www.ncbi.nlm.nih.gov/entrez/viewer.fcgi?db=nucleotide&val=NM_014159.6) | HBP231\|HIF-1\|HIP-1\|HYPB\|KMT3A\|SET2\|p231HBP | SET domain containing 2 |
| SFRP1 | [NM_003012.3](http://www.ncbi.nlm.nih.gov/entrez/viewer.fcgi?db=nucleotide&val=NM_003012.3) | FRP\|FRP-1\|FRP1\|FrzA\|SARP2 | secreted frizzled-related protein 1 |
| SFRP2 | [NM_003013.2](http://www.ncbi.nlm.nih.gov/entrez/viewer.fcgi?db=nucleotide&val=NM_003013.2) | FRP-2\|SARP1\|SDF-5 | secreted frizzled-related protein 2 |
| SH2B3 | [NM_005475.2](http://www.ncbi.nlm.nih.gov/entrez/viewer.fcgi?db=nucleotide&val=NM_005475.2) | IDDM20\|LNK | SH2B adaptor protein 3 |
| SH2D3A | [NM_005490.2](http://www.ncbi.nlm.nih.gov/entrez/viewer.fcgi?db=nucleotide&val=NM_005490.2) | NSP1 | SH2 domain containing 3A |
| SH3YL1 | [NM_001159597.1](http://www.ncbi.nlm.nih.gov/entrez/viewer.fcgi?db=nucleotide&val=NM_001159597.1) | RAY | SH3 domain containing, Ysc84-like 1 (S. cerevisiae) |
| SHB | [NM_003028.2](http://www.ncbi.nlm.nih.gov/entrez/viewer.fcgi?db=nucleotide&val=NM_003028.2) | RP11-3J10.8\|bA3J10.2 | Src homology 2 domain containing adaptor protein B |
| SIRT1 | [NM_012238.4](http://www.ncbi.nlm.nih.gov/entrez/viewer.fcgi?db=nucleotide&val=NM_012238.4) | SIR2L1 | sirtuin 1 |
| SKP1 | [NM_170679.2](http://www.ncbi.nlm.nih.gov/entrez/viewer.fcgi?db=nucleotide&val=NM_170679.2) | EMC19\|OCP-II\|OCP2\|SKP1A\|TCEB1L\|p19A | S-phase kinase-associated protein 1 |
| SLC12A6 | [NM_001042494.1](http://www.ncbi.nlm.nih.gov/entrez/viewer.fcgi?db=nucleotide&val=NM_001042494.1) | ACCPN\|KCC3\|KCC3A\|KCC3B | solute carrier family 12 (potassium/chloride transporters), member 6 |
| SLC2A1 | [NM_006516.2](http://www.ncbi.nlm.nih.gov/entrez/viewer.fcgi?db=nucleotide&val=NM_006516.2) | DYT17\|DYT18\|GLUT\|GLUT1\|GLUT1DS\|PED | solute carrier family 2 (facilitated glucose transporter), member 1 |
| SLC35A3 | [NM_012243.1](http://www.ncbi.nlm.nih.gov/entrez/viewer.fcgi?db=nucleotide&val=NM_012243.1) | - | solute carrier family 35 (UDP-N-acetylglucosamine (UDP-GlcNAc) transporter), member A3 |
| SLC37A1 | [NM_018964.3](http://www.ncbi.nlm.nih.gov/entrez/viewer.fcgi?db=nucleotide&val=NM_018964.3) | G3PP | solute carrier family 37 (glycerol-3-phosphate transporter), member 1 |
| SLC44A4 | [NM_032794.1](http://www.ncbi.nlm.nih.gov/entrez/viewer.fcgi?db=nucleotide&val=NM_032794.1) | C6orf29\|CTL4\|NG22 | solute carrier family 44, member 4 |
| SLIT2 | [NM_004787.1](http://www.ncbi.nlm.nih.gov/entrez/viewer.fcgi?db=nucleotide&val=NM_004787.1) | SLIL3\|Slit-2 | slit homolog 2 (Drosophila) |
| SLPI | [NM_003064.2](http://www.ncbi.nlm.nih.gov/entrez/viewer.fcgi?db=nucleotide&val=NM_003064.2) | ALK1\|ALP\|BLPI\|HUSI\|HUSI-I\|MPI\|WAP4\|WFDC4 | secretory leukocyte peptidase inhibitor |
| SMAD1 | [NM_005900.2](http://www.ncbi.nlm.nih.gov/entrez/viewer.fcgi?db=nucleotide&val=NM_005900.2) | BSP-1\|BSP1\|JV4-1\|JV41\|MADH1\|MADR1 | SMAD family member 1 |
| SMAD2 | [NM_005901.5](http://www.ncbi.nlm.nih.gov/entrez/viewer.fcgi?db=nucleotide&val=NM_005901.5) | JV18\|JV18-1\|MADH2\|MADR2\|hMAD-2\|hSMAD2 | SMAD family member 2 |
| SMAD3 | [NM_005902.3](http://www.ncbi.nlm.nih.gov/entrez/viewer.fcgi?db=nucleotide&val=NM_005902.3) | HSPC193\|HsT17436\|JV15-2\|LDS1C\|MADH3 | SMAD family member 3 |
| SMAD4 | [NM_005359.3](http://www.ncbi.nlm.nih.gov/entrez/viewer.fcgi?db=nucleotide&val=NM_005359.3) | DPC4\|JIP\|MADH4\|MYHRS | SMAD family member 4 |
| SMAD5 | [NM_005903.5](http://www.ncbi.nlm.nih.gov/entrez/viewer.fcgi?db=nucleotide&val=NM_005903.5) | DWFC\|JV5-1\|MADH5 | SMAD family member 5 |
| SMAD9 | [NM_001127217.2](http://www.ncbi.nlm.nih.gov/entrez/viewer.fcgi?db=nucleotide&val=NM_001127217.2) | MADH6\|MADH9\|SMAD8\|SMAD8A\|SMAD8B | SMAD family member 9 |
| SMC3 | [NM_005445.3](http://www.ncbi.nlm.nih.gov/entrez/viewer.fcgi?db=nucleotide&val=NM_005445.3) | BAM\|BMH\|CDLS3\|CSPG6\|HCAP\|SMC3L1 | structural maintenance of chromosomes 3 |
| SMOC1 | [NM_001034852.1](http://www.ncbi.nlm.nih.gov/entrez/viewer.fcgi?db=nucleotide&val=NM_001034852.1) | OAS | SPARC related modular calcium binding 1 |
| SMURF1 | [NM_181349.1](http://www.ncbi.nlm.nih.gov/entrez/viewer.fcgi?db=nucleotide&val=NM_181349.1) | - | SMAD specific E3 ubiquitin protein ligase 1 |
| SMURF2 | [NM_022739.3](http://www.ncbi.nlm.nih.gov/entrez/viewer.fcgi?db=nucleotide&val=NM_022739.3) | - | SMAD specific E3 ubiquitin protein ligase 2 |
| SNAI1 | [NM_005985.2](http://www.ncbi.nlm.nih.gov/entrez/viewer.fcgi?db=nucleotide&val=NM_005985.2) | SLUGH2\|SNA\|SNAH\|SNAIL\|SNAIL1\|dJ710H13.1 | snail homolog 1 (Drosophila) |
| SNAI2 | [NM_003068.3](http://www.ncbi.nlm.nih.gov/entrez/viewer.fcgi?db=nucleotide&val=NM_003068.3) | SLUG\|SLUGH1\|SNAIL2\|WS2D | snail homolog 2 (Drosophila) |
| SNAI3 | [NM_178310.1](http://www.ncbi.nlm.nih.gov/entrez/viewer.fcgi?db=nucleotide&val=NM_178310.1) | SMUC\|SNAIL3\|ZNF293\|Zfp293 | snail homolog 3 (Drosophila) |
| SNRPF | [NM_003095.2](http://www.ncbi.nlm.nih.gov/entrez/viewer.fcgi?db=nucleotide&val=NM_003095.2) | SMF\|Sm-F\|snRNP-F | small nuclear ribonucleoprotein polypeptide F |
| SOD1 | [NM_000454.4](http://www.ncbi.nlm.nih.gov/entrez/viewer.fcgi?db=nucleotide&val=NM_000454.4) | ALS\|ALS1\|IPOA\|SOD\|hSod1\|homodimer | superoxide dismutase 1, soluble |
| SORD | [NM_003104.4](http://www.ncbi.nlm.nih.gov/entrez/viewer.fcgi?db=nucleotide&val=NM_003104.4) | SORD1 | sorbitol dehydrogenase |
| SOX17 | [NM_022454.3](http://www.ncbi.nlm.nih.gov/entrez/viewer.fcgi?db=nucleotide&val=NM_022454.3) | VUR3 | SRY (sex determining region Y)-box 17 |
| SOX2 | [NM_003106.2](http://www.ncbi.nlm.nih.gov/entrez/viewer.fcgi?db=nucleotide&val=NM_003106.2) | ANOP3\|MCOPS3 | SRY (sex determining region Y)-box 2 |
| SOX9 | [NM_000346.2](http://www.ncbi.nlm.nih.gov/entrez/viewer.fcgi?db=nucleotide&val=NM_000346.2) | CMD1\|CMPD1\|SRA1 | SRY (sex determining region Y)-box 9 |
| SP1 | [NM_003109.1](http://www.ncbi.nlm.nih.gov/entrez/viewer.fcgi?db=nucleotide&val=NM_003109.1) | - | Sp1 transcription factor |
| SPARC | [NM_003118.2](http://www.ncbi.nlm.nih.gov/entrez/viewer.fcgi?db=nucleotide&val=NM_003118.2) | ON | secreted protein, acidic, cysteine-rich (osteonectin) |
| SPARCL1 | [NM_004684.4](http://www.ncbi.nlm.nih.gov/entrez/viewer.fcgi?db=nucleotide&val=NM_004684.4) | PIG33\|SC1 | SPARC-like 1 (hevin) |
| SPDEF | [NM_012391.1](http://www.ncbi.nlm.nih.gov/entrez/viewer.fcgi?db=nucleotide&val=NM_012391.1) | PDEF\|RP11-375E1__A.3\|bA375E1.3 | SAM pointed domain containing ets transcription factor |
| SPHK2 | [NM_020126.3](http://www.ncbi.nlm.nih.gov/entrez/viewer.fcgi?db=nucleotide&val=NM_020126.3) | SK 2\|SK-2\|SPK 2\|SPK-2 | sphingosine kinase 2 |
| SPINK5 | [NM_006846.3](http://www.ncbi.nlm.nih.gov/entrez/viewer.fcgi?db=nucleotide&val=NM_006846.3) | LEKTI\|LETKI\|NETS\|NS\|VAKTI | serine peptidase inhibitor, Kazal type 5 |
| SPINT1 | [NM_001032367.1](http://www.ncbi.nlm.nih.gov/entrez/viewer.fcgi?db=nucleotide&val=NM_001032367.1) | HAI\|HAI1\|MANSC2 | serine peptidase inhibitor, Kunitz type 1 |
| SPOCK3 | [NM_001204355.1](http://www.ncbi.nlm.nih.gov/entrez/viewer.fcgi?db=nucleotide&val=NM_001204355.1) | HSAJ1454\|TES-3\|TICN3 | sparc/osteonectin, cwcv and kazal-like domains proteoglycan (testican) 3 |
| SPP1 | [NM_000582.2](http://www.ncbi.nlm.nih.gov/entrez/viewer.fcgi?db=nucleotide&val=NM_000582.2) | BNSP\|BSPI\|ETA-1\|OPN | secreted phosphoprotein 1 |
| SRC | [NM_005417.3](http://www.ncbi.nlm.nih.gov/entrez/viewer.fcgi?db=nucleotide&val=NM_005417.3) | ASV\|SRC1\|c-SRC\|p60-Src | v-src sarcoma (Schmidt-Ruppin A-2) viral oncogene homolog (avian) |
| SRF | [NM_003131.2](http://www.ncbi.nlm.nih.gov/entrez/viewer.fcgi?db=nucleotide&val=NM_003131.2) | MCM1 | serum response factor (c-fos serum response element-binding transcription factor) |
| SRGN | [NR_036430.1](http://www.ncbi.nlm.nih.gov/entrez/viewer.fcgi?db=nucleotide&val=NR_036430.1) | PPG\|PRG\|PRG1 | serglycin |
| SRPK2 | [NM_182692.1](http://www.ncbi.nlm.nih.gov/entrez/viewer.fcgi?db=nucleotide&val=NM_182692.1) | SFRSK2 | SRSF protein kinase 2 |
| SRPX2 | [NM_014467.2](http://www.ncbi.nlm.nih.gov/entrez/viewer.fcgi?db=nucleotide&val=NM_014467.2) | BPP\|CBPS\|PMGX\|RESDX\|SRPUL | sushi-repeat containing protein, X-linked 2 |
| SSTR2 | [NM_001050.2](http://www.ncbi.nlm.nih.gov/entrez/viewer.fcgi?db=nucleotide&val=NM_001050.2) | - | somatostatin receptor 2 |
| ST14 | [NM_021978.3](http://www.ncbi.nlm.nih.gov/entrez/viewer.fcgi?db=nucleotide&val=NM_021978.3) | HAI\|MT-SP1\|MTSP1\|PRSS14\|SNC19\|TADG15\|TMPRSS14 | suppression of tumorigenicity 14 (colon carcinoma) |
| STAB1 | [NM_015136.2](http://www.ncbi.nlm.nih.gov/entrez/viewer.fcgi?db=nucleotide&val=NM_015136.2) | CLEVER-1\|FEEL-1\|FELE-1\|FEX1\|STAB-1 | stabilin 1 |
| STAB2 | [NM_017564.9](http://www.ncbi.nlm.nih.gov/entrez/viewer.fcgi?db=nucleotide&val=NM_017564.9) | FEEL2\|FELE-2\|FELL2\|FEX2\|HARE | stabilin 2 |
| STAT1 | [NM_139266.1](http://www.ncbi.nlm.nih.gov/entrez/viewer.fcgi?db=nucleotide&val=NM_139266.1) | CANDF7\|ISGF-3\|STAT91 | signal transducer and activator of transcription 1, 91kDa |
| STAT3 | [NM_139276.2](http://www.ncbi.nlm.nih.gov/entrez/viewer.fcgi?db=nucleotide&val=NM_139276.2) | APRF\|HIES | signal transducer and activator of transcription 3 (acute-phase response factor) |
| SULF1 | [NM_001128204.1](http://www.ncbi.nlm.nih.gov/entrez/viewer.fcgi?db=nucleotide&val=NM_001128204.1) | HSULF-1\|SULF-1 | sulfatase 1 |
| SV2B | [NM_001167580.1](http://www.ncbi.nlm.nih.gov/entrez/viewer.fcgi?db=nucleotide&val=NM_001167580.1) | HsT19680 | synaptic vesicle glycoprotein 2B |
| SYK | [NM_003177.3](http://www.ncbi.nlm.nih.gov/entrez/viewer.fcgi?db=nucleotide&val=NM_003177.3) | p72-Syk | spleen tyrosine kinase |
| SYNE1 | [NM_015293.1](http://www.ncbi.nlm.nih.gov/entrez/viewer.fcgi?db=nucleotide&val=NM_015293.1) | 8B\|ARCA1\|C6orf98\|CPG2\|EDMD4\|MYNE1\|Nesp1\|SCAR8\|dJ45H2.2 | spectrin repeat containing, nuclear envelope 1 |
| TACSTD2 | [NM_002353.2](http://www.ncbi.nlm.nih.gov/entrez/viewer.fcgi?db=nucleotide&val=NM_002353.2) | EGP-1\|EGP1\|GA733-1\|GA7331\|GP50\|M1S1\|TROP2 | tumor-associated calcium signal transducer 2 |
| TAL1 | [NM_003189.2](http://www.ncbi.nlm.nih.gov/entrez/viewer.fcgi?db=nucleotide&val=NM_003189.2) | SCL\|TCL5\|bHLHa17\|tal-1 | T-cell acute lymphocytic leukemia 1 |
| TBX1 | [NM_080646.1](http://www.ncbi.nlm.nih.gov/entrez/viewer.fcgi?db=nucleotide&val=NM_080646.1) | CAFS\|CTHM\|DGCR\|DGS\|DORV\|TBX1C\|TGA\|VCFS | T-box 1 |
| TBX4 | [NM_018488.2](http://www.ncbi.nlm.nih.gov/entrez/viewer.fcgi?db=nucleotide&val=NM_018488.2) | SPS | T-box 4 |
| TBXA2R | [NM_001060.3](http://www.ncbi.nlm.nih.gov/entrez/viewer.fcgi?db=nucleotide&val=NM_001060.3) | BDPLT13\|TXA2-R | thromboxane A2 receptor |
| TCEB1 | [NM_001204857.1](http://www.ncbi.nlm.nih.gov/entrez/viewer.fcgi?db=nucleotide&val=NM_001204857.1) | SIII\|eloC | transcription elongation factor B (SIII), polypeptide 1 (15kDa, elongin C) |
| TCEB2 | [NM_007108.2](http://www.ncbi.nlm.nih.gov/entrez/viewer.fcgi?db=nucleotide&val=NM_007108.2) | ELOB\|SIII | transcription elongation factor B (SIII), polypeptide 2 (18kDa, elongin B) |
| TCF20 | [NM_005650.1](http://www.ncbi.nlm.nih.gov/entrez/viewer.fcgi?db=nucleotide&val=NM_005650.1) | AR1\|SPBP | transcription factor 20 (AR1) |
| TCF3 | [NM_003200.3](http://www.ncbi.nlm.nih.gov/entrez/viewer.fcgi?db=nucleotide&val=NM_003200.3) | E2A\|E47\|ITF1\|TCF-3\|VDIR\|bHLHb21 | transcription factor 3 (E2A immunoglobulin enhancer binding factors E12/E47) |
| TCF4 | [NM_003199.1](http://www.ncbi.nlm.nih.gov/entrez/viewer.fcgi?db=nucleotide&val=NM_003199.1) | E2-2\|ITF-2\|ITF2\|PTHS\|SEF-2\|SEF2\|SEF2-1\|SEF2-1A\|SEF2-1B\|TCF-4\|bHLHb19 | transcription factor 4 |
| TDGF1 | [NM_003212.2](http://www.ncbi.nlm.nih.gov/entrez/viewer.fcgi?db=nucleotide&val=NM_003212.2) | CR\|CRGF\|CRIPTO | teratocarcinoma-derived growth factor 1 |
| TEK | [NM_000459.3](http://www.ncbi.nlm.nih.gov/entrez/viewer.fcgi?db=nucleotide&val=NM_000459.3) | CD202B\|TIE-2\|TIE2\|VMCM\|VMCM1 | TEK tyrosine kinase, endothelial |
| TF | [NM_001063.2](http://www.ncbi.nlm.nih.gov/entrez/viewer.fcgi?db=nucleotide&val=NM_001063.2) | PRO1557\|PRO2086\|TFQTL1 | transferrin |
| TFDP1 | [NM_007111.4](http://www.ncbi.nlm.nih.gov/entrez/viewer.fcgi?db=nucleotide&val=NM_007111.4) | DP1\|DRTF1\|Dp-1 | transcription factor Dp-1 |
| TFPI2 | [NM_006528.3](http://www.ncbi.nlm.nih.gov/entrez/viewer.fcgi?db=nucleotide&val=NM_006528.3) | PP5\|REF1\|TFPI-2 | tissue factor pathway inhibitor 2 |
| TGFB1 | [NM_000660.3](http://www.ncbi.nlm.nih.gov/entrez/viewer.fcgi?db=nucleotide&val=NM_000660.3) | CED\|DPD1\|LAP\|TGFB\|TGFbeta | transforming growth factor, beta 1 |
| TGFB2 | [NM_003238.2](http://www.ncbi.nlm.nih.gov/entrez/viewer.fcgi?db=nucleotide&val=NM_003238.2) | TGF-beta2 | transforming growth factor, beta 2 |
| TGFBI | [NM_000358.2](http://www.ncbi.nlm.nih.gov/entrez/viewer.fcgi?db=nucleotide&val=NM_000358.2) | BIGH3\|CDB1\|CDG2\|CDGG1\|CSD\|CSD1\|CSD2\|CSD3\|EBMD\|LCD1 | transforming growth factor, beta-induced, 68kDa |
| TGFBR2 | [NM_001024847.1](http://www.ncbi.nlm.nih.gov/entrez/viewer.fcgi?db=nucleotide&val=NM_001024847.1) | AAT3\|FAA3\|LDS1B\|LDS2B\|MFS2\|RIIC\|TAAD2\|TGFR-2\|TGFbeta-RII | transforming growth factor, beta receptor II (70/80kDa) |
| THBS1 | [NM_003246.2](http://www.ncbi.nlm.nih.gov/entrez/viewer.fcgi?db=nucleotide&val=NM_003246.2) | THBS\|THBS-1\|TSP\|TSP-1\|TSP1 | thrombospondin 1 |
| THBS2 | [NM_003247.2](http://www.ncbi.nlm.nih.gov/entrez/viewer.fcgi?db=nucleotide&val=NM_003247.2) | TSP2 | thrombospondin 2 |
| THBS4 | [NM_003248.3](http://www.ncbi.nlm.nih.gov/entrez/viewer.fcgi?db=nucleotide&val=NM_003248.3) | TSP4 | thrombospondin 4 |
| THY1 | [NM_006288.2](http://www.ncbi.nlm.nih.gov/entrez/viewer.fcgi?db=nucleotide&val=NM_006288.2) | CD90 | Thy-1 cell surface antigen |
| TIE1 | [NM_005424.2](http://www.ncbi.nlm.nih.gov/entrez/viewer.fcgi?db=nucleotide&val=NM_005424.2) | JTK14\|TIE | tyrosine kinase with immunoglobulin-like and EGF-like domains 1 |
| TIMP1 | [NM_003254.2](http://www.ncbi.nlm.nih.gov/entrez/viewer.fcgi?db=nucleotide&val=NM_003254.2) | CLGI\|EPA\|EPO\|HCI\|TIMP | TIMP metallopeptidase inhibitor 1 |
| TIMP2 | [NM_003255.4](http://www.ncbi.nlm.nih.gov/entrez/viewer.fcgi?db=nucleotide&val=NM_003255.4) | CSC-21K | TIMP metallopeptidase inhibitor 2 |
| TIMP4 | [NM_003256.2](http://www.ncbi.nlm.nih.gov/entrez/viewer.fcgi?db=nucleotide&val=NM_003256.2) | - | TIMP metallopeptidase inhibitor 4 |
| TJP2 | [NM_004817.2](http://www.ncbi.nlm.nih.gov/entrez/viewer.fcgi?db=nucleotide&val=NM_004817.2) | C9DUPq21.11\|DFNA51\|DUP9q21.11\|X104\|ZO2 | tight junction protein 2 |
| TJP3 | [NM_014428.1](http://www.ncbi.nlm.nih.gov/entrez/viewer.fcgi?db=nucleotide&val=NM_014428.1) | ZO-3\|ZO3 | tight junction protein 3 |
| TLR4 | [NR_024168.1](http://www.ncbi.nlm.nih.gov/entrez/viewer.fcgi?db=nucleotide&val=NR_024168.1) | ARMD10\|CD284\|TLR-4\|TOLL | toll-like receptor 4 |
| TMC6 | [NM_001127198.1](http://www.ncbi.nlm.nih.gov/entrez/viewer.fcgi?db=nucleotide&val=NM_001127198.1) | EV1\|EVER1\|EVIN1\|LAK-4P | transmembrane channel-like 6 |
| TMEM100 | [NM_018286.2](http://www.ncbi.nlm.nih.gov/entrez/viewer.fcgi?db=nucleotide&val=NM_018286.2) | - | transmembrane protein 100 |
| TMEM30B | [NM_001017970.2](http://www.ncbi.nlm.nih.gov/entrez/viewer.fcgi?db=nucleotide&val=NM_001017970.2) | CDC50B | transmembrane protein 30B |
| TMPRSS2 | [NM_005656.3](http://www.ncbi.nlm.nih.gov/entrez/viewer.fcgi?db=nucleotide&val=NM_005656.3) | PP9284\|PRSS10 | transmembrane protease, serine 2 |
| TMPRSS4 | [NM_019894.3](http://www.ncbi.nlm.nih.gov/entrez/viewer.fcgi?db=nucleotide&val=NM_019894.3) | CAPH2\|MT-SP2\|TMPRSS3 | transmembrane protease, serine 4 |
| TMPRSS6 | [NM_153609.2](http://www.ncbi.nlm.nih.gov/entrez/viewer.fcgi?db=nucleotide&val=NM_153609.2) | IRIDA | transmembrane protease, serine 6 |
| TNC | [NM_002160.3](http://www.ncbi.nlm.nih.gov/entrez/viewer.fcgi?db=nucleotide&val=NM_002160.3) | 150-225\|GMEM\|GP\|HXB\|JI\|TN\|TN-C | tenascin C |
| TNF | [NM_000594.2](http://www.ncbi.nlm.nih.gov/entrez/viewer.fcgi?db=nucleotide&val=NM_000594.2) | DIF\|TNF-alpha\|TNFA\|TNFSF2 | tumor necrosis factor |
| TNFRSF12A | [NM_016639.1](http://www.ncbi.nlm.nih.gov/entrez/viewer.fcgi?db=nucleotide&val=NM_016639.1) | CD266\|FN14\|TWEAKR | tumor necrosis factor receptor superfamily, member 12A |
| TNFRSF1A | [NM_001065.2](http://www.ncbi.nlm.nih.gov/entrez/viewer.fcgi?db=nucleotide&val=NM_001065.2) | CD120a\|FPF\|TBP1\|TNF-R\|TNF-R-I\|TNF-R55\|TNFAR\|TNFR1\|TNFR55\|TNFR60\|p55\|p55-R\|p60 | tumor necrosis factor receptor superfamily, member 1A |
| TNFSF10 | [NM_003810.2](http://www.ncbi.nlm.nih.gov/entrez/viewer.fcgi?db=nucleotide&val=NM_003810.2) | APO2L\|Apo-2L\|CD253\|TL2\|TRAIL | tumor necrosis factor (ligand) superfamily, member 10 |
| TNFSF12 | [NM_003809.2](http://www.ncbi.nlm.nih.gov/entrez/viewer.fcgi?db=nucleotide&val=NM_003809.2) | APO3L\|DR3LG\|TWEAK | tumor necrosis factor (ligand) superfamily, member 12 |
| TNFSF13 | [NM_003808.3](http://www.ncbi.nlm.nih.gov/entrez/viewer.fcgi?db=nucleotide&val=NM_003808.3) | APRIL\|CD256\|TALL-2\|TALL2\|TRDL-1\|ZTNF2 | tumor necrosis factor (ligand) superfamily, member 13 |
| TNMD | [NM_022144.2](http://www.ncbi.nlm.nih.gov/entrez/viewer.fcgi?db=nucleotide&val=NM_022144.2) | BRICD4\|CHM1L\|TEM | tenomodulin |
| TNN | [NM_022093.1](http://www.ncbi.nlm.nih.gov/entrez/viewer.fcgi?db=nucleotide&val=NM_022093.1) | TN-W | tenascin N |
| TNS1 | [NM_022648.4](http://www.ncbi.nlm.nih.gov/entrez/viewer.fcgi?db=nucleotide&val=NM_022648.4) | MST091\|MST122\|MST127\|MSTP122\|MSTP127\|MXRA6\|TNS | tensin 1 |
| TNXB | [NM_032470.3](http://www.ncbi.nlm.nih.gov/entrez/viewer.fcgi?db=nucleotide&val=NM_032470.3) | EDS3\|HXBL\|TENX\|TN-X\|TNX\|TNXB1\|TNXB2\|TNXBS\|XB\|XBS | tenascin XB |
| TOM1L1 | [NM_005486.2](http://www.ncbi.nlm.nih.gov/entrez/viewer.fcgi?db=nucleotide&val=NM_005486.2) | OK/KNS-CL.3\|SRCASM | target of myb1 (chicken)-like 1 |
| TP53 | [NM_000546.2](http://www.ncbi.nlm.nih.gov/entrez/viewer.fcgi?db=nucleotide&val=NM_000546.2) | BCC7\|LFS1\|P53\|TRP53 | tumor protein p53 |
| TPM2 | [NM_003289.3](http://www.ncbi.nlm.nih.gov/entrez/viewer.fcgi?db=nucleotide&val=NM_003289.3) | AMCD1\|DA1\|DA2B\|NEM4\|TMSB | tropomyosin 2 (beta) |
| TPSB2 | [NM_024164.5](http://www.ncbi.nlm.nih.gov/entrez/viewer.fcgi?db=nucleotide&val=NM_024164.5) | TPS2\|tryptaseB\|tryptaseC | tryptase beta 2 (gene/pseudogene) |
| TPSD1 | [NM_012217.2](http://www.ncbi.nlm.nih.gov/entrez/viewer.fcgi?db=nucleotide&val=NM_012217.2) | MCP7-LIKE\|MCP7L1\|MMCP-7L | tryptase delta 1 |
| TSHR | [NM_001018036.2](http://www.ncbi.nlm.nih.gov/entrez/viewer.fcgi?db=nucleotide&val=NM_001018036.2) | CHNG1\|LGR3\|hTSHR-I | thyroid stimulating hormone receptor |
| TSPAN1 | [NM_005727.2](http://www.ncbi.nlm.nih.gov/entrez/viewer.fcgi?db=nucleotide&val=NM_005727.2) | NET1\|TM4C\|TM4SF | tetraspanin 1 |
| TWIST1 | [NM_000474.3](http://www.ncbi.nlm.nih.gov/entrez/viewer.fcgi?db=nucleotide&val=NM_000474.3) | ACS3\|BPES2\|BPES3\|CRS1\|SCS\|TWIST\|bHLHa38 | twist homolog 1 (Drosophila) |
| TWIST2 | [NM_057179.2](http://www.ncbi.nlm.nih.gov/entrez/viewer.fcgi?db=nucleotide&val=NM_057179.2) | DERMO1\|SETLSS\|bHLHa39 | twist homolog 2 (Drosophila) |
| TXNIP | [NM_006472.1](http://www.ncbi.nlm.nih.gov/entrez/viewer.fcgi?db=nucleotide&val=NM_006472.1) | EST01027\|HHCPA78\|THIF\|VDUP1 | thioredoxin interacting protein |
| TYMP | [NM_001953.3](http://www.ncbi.nlm.nih.gov/entrez/viewer.fcgi?db=nucleotide&val=NM_001953.3) | ECGF\|ECGF1\|MEDPS1\|MNGIE\|MTDPS1\|PDECGF\|TP\|hPD-ECGF | thymidine phosphorylase |
| UBA52 | [NM_003333.3](http://www.ncbi.nlm.nih.gov/entrez/viewer.fcgi?db=nucleotide&val=NM_003333.3) | CEP52\|HUBCEP52\|L40\|RPL40 | ubiquitin A-52 residue ribosomal protein fusion product 1 |
| UTS2 | [NM_006786.3](http://www.ncbi.nlm.nih.gov/entrez/viewer.fcgi?db=nucleotide&val=NM_006786.3) | PRO1068\|U-II\|UCN2\|UII | urotensin 2 |
| VAMP8 | [NM_003761.3](http://www.ncbi.nlm.nih.gov/entrez/viewer.fcgi?db=nucleotide&val=NM_003761.3) | EDB\|VAMP-8 | vesicle-associated membrane protein 8 (endobrevin) |
| VASH1 | [NM_014909.4](http://www.ncbi.nlm.nih.gov/entrez/viewer.fcgi?db=nucleotide&val=NM_014909.4) | KIAA1036 | vasohibin 1 |
| VAV2 | [NM_003371.3](http://www.ncbi.nlm.nih.gov/entrez/viewer.fcgi?db=nucleotide&val=NM_003371.3) | VAV-2 | vav 2 guanine nucleotide exchange factor |
| VAV3 | [NM_001079874.1](http://www.ncbi.nlm.nih.gov/entrez/viewer.fcgi?db=nucleotide&val=NM_001079874.1) | - | vav 3 guanine nucleotide exchange factor |
| VCAM1 | [NM_001078.3](http://www.ncbi.nlm.nih.gov/entrez/viewer.fcgi?db=nucleotide&val=NM_001078.3) | CD106\|INCAM-100 | vascular cell adhesion molecule 1 |
| VCAN | [NM_004385.3](http://www.ncbi.nlm.nih.gov/entrez/viewer.fcgi?db=nucleotide&val=NM_004385.3) | CSPG2\|ERVR\|GHAP\|PG-M\|WGN\|WGN1 | versican |
| VEGFA | [NM_001025366.1](http://www.ncbi.nlm.nih.gov/entrez/viewer.fcgi?db=nucleotide&val=NM_001025366.1) | MVCD1\|VEGF\|VPF | vascular endothelial growth factor A |
| VEGFB | [NM_003377.3](http://www.ncbi.nlm.nih.gov/entrez/viewer.fcgi?db=nucleotide&val=NM_003377.3) | VEGFL\|VRF | vascular endothelial growth factor B |
| VEGFC | [NM_005429.2](http://www.ncbi.nlm.nih.gov/entrez/viewer.fcgi?db=nucleotide&val=NM_005429.2) | Flt4-L\|VRP | vascular endothelial growth factor C |
| VEZF1 | [NM_007146.2](http://www.ncbi.nlm.nih.gov/entrez/viewer.fcgi?db=nucleotide&val=NM_007146.2) | DB1\|ZNF161 | vascular endothelial zinc finger 1 |
| VHL | [NM_000551.2](http://www.ncbi.nlm.nih.gov/entrez/viewer.fcgi?db=nucleotide&val=NM_000551.2) | HRCA1\|RCA1\|VHL1\|pVHL | von Hippel-Lindau tumor suppressor, E3 ubiquitin protein ligase |
| VIM | [NM_003380.2](http://www.ncbi.nlm.nih.gov/entrez/viewer.fcgi?db=nucleotide&val=NM_003380.2) | - | vimentin |
| VIT | [NM_053276.3](http://www.ncbi.nlm.nih.gov/entrez/viewer.fcgi?db=nucleotide&val=NM_053276.3) | - | vitrin |
| VPS13A | [NM_033305.2](http://www.ncbi.nlm.nih.gov/entrez/viewer.fcgi?db=nucleotide&val=NM_033305.2) | CHAC\|CHOREIN | vacuolar protein sorting 13 homolog A (S. cerevisiae) |
| VSIG4 | [NM_001100431.1](http://www.ncbi.nlm.nih.gov/entrez/viewer.fcgi?db=nucleotide&val=NM_001100431.1) | CRIg\|Z39IG | V-set and immunoglobulin domain containing 4 |
| VWA1 | [NM_199121.2](http://www.ncbi.nlm.nih.gov/entrez/viewer.fcgi?db=nucleotide&val=NM_199121.2) | WARP | von Willebrand factor A domain containing 1 |
| VWA2 | [NM_001272046.1](http://www.ncbi.nlm.nih.gov/entrez/viewer.fcgi?db=nucleotide&val=NM_001272046.1) | AMACO\|CCSP-2\|NET42 | von Willebrand factor A domain containing 2 |
| WARS | [NM_004184.3](http://www.ncbi.nlm.nih.gov/entrez/viewer.fcgi?db=nucleotide&val=NM_004184.3) | GAMMA-2\|IFI53\|IFP53 | tryptophanyl-tRNA synthetase |
| WIPF1 | [NM_001077269.1](http://www.ncbi.nlm.nih.gov/entrez/viewer.fcgi?db=nucleotide&val=NM_001077269.1) | PRPL-2\|WASPIP\|WIP | WAS/WASL interacting protein family, member 1 |
| WNT5A | [NM_003392.3](http://www.ncbi.nlm.nih.gov/entrez/viewer.fcgi?db=nucleotide&val=NM_003392.3) | hWNT5A | wingless-type MMTV integration site family, member 5A |
| WNT5B | [NM_032642.2](http://www.ncbi.nlm.nih.gov/entrez/viewer.fcgi?db=nucleotide&val=NM_032642.2) | - | wingless-type MMTV integration site family, member 5B |
| WWTR1 | [NM_001168278.1](http://www.ncbi.nlm.nih.gov/entrez/viewer.fcgi?db=nucleotide&val=NM_001168278.1) | TAZ | WW domain containing transcription regulator 1 |
| ZC3H12A | [NM_025079.2](http://www.ncbi.nlm.nih.gov/entrez/viewer.fcgi?db=nucleotide&val=NM_025079.2) | MCPIP\|MCPIP1\|RP3-423B22.1\|dJ423B22.1 | zinc finger CCCH-type containing 12A |
| ZCCHC24 | [XM_005269604.1](http://www.ncbi.nlm.nih.gov/entrez/viewer.fcgi?db=nucleotide&val=XM_005269604.1) | C10orf56 | zinc finger, CCHC domain containing 24 |
| ZEB1 | [NM_001128128.1](http://www.ncbi.nlm.nih.gov/entrez/viewer.fcgi?db=nucleotide&val=NM_001128128.1) | AREB6\|BZP\|DELTAEF1\|FECD6\|NIL2A\|PPCD3\|TCF8\|ZFHEP\|ZFHX1A | zinc finger E-box binding homeobox 1 |
| ZEB2 | [NM_014795.3](http://www.ncbi.nlm.nih.gov/entrez/viewer.fcgi?db=nucleotide&val=NM_014795.3) | HSPC082\|SIP-1\|SIP1\|SMADIP1\|ZFHX1B | zinc finger E-box binding homeobox 2 |
| ZFPM2 | [NM_012082.3](http://www.ncbi.nlm.nih.gov/entrez/viewer.fcgi?db=nucleotide&val=NM_012082.3) | DIH3\|FOG2\|ZNF89B\|hFOG-2 | zinc finger protein, multitype 2 |
| ZFYVE16 | [NM_001105251.2](http://www.ncbi.nlm.nih.gov/entrez/viewer.fcgi?db=nucleotide&val=NM_001105251.2) | ENDOFIN\|PPP1R69 | zinc finger, FYVE domain containing 16 |
| ZFYVE9 | [NM_004799.2](http://www.ncbi.nlm.nih.gov/entrez/viewer.fcgi?db=nucleotide&val=NM_004799.2) | MADHIP\|NSP\|SARA\|SMADIP | zinc finger, FYVE domain containing 9 |
