# Supplementary material for: Cancer‐associated stroma reveals prognostic biomarkers and novel insights into the tumour microenvironment of colorectal cancer and colorectal liver metastases
Source: Cancer Med. 2021 Dec 7;11(2):492–506. doi: 10.1002/cam4.4452 (PMC8729056; doi:10.1002/cam4.4452)
Supplement: Supplementary file 3 — Table S2‐S7 [file CAM4-11-492-s001.docx]

**Supplementary Tables**

| Supplementary Table 2 – Baseline CRLM Characteristics | | |
| --- | --- | --- |
|  |  | **Overall** |
|  | **n** | 110 |
| Sex = Male/Female (%) |  | 68/42 (61.8/38.2) |
| Age at liver resection (median [IQR]) |  | 64.25 [56.78, 71.77] |
| Year of liver resection (%) | 1998-2002 | 7 (6.4) |
|  | 2003-2007 | 44 (40.0) |
|  | 2008-2012 | 48 (43.6) |
|  | 2013-2017 | 11 (10.0) |
| Follow up in months (median [IQR]) |  | 44.91 [24.85, 80.75] |
| Timing of metastasis = Metachronous (%) |  | 54 (49.1) |
| Primary CRC lymph node metastases = Yes (%) |  | 68 (61.8) |
| Margin status primary CRC resection (%) | R0 | 74 (59.7) |
|  | R1 | 1 (0.8) |
|  | NA | 49 (39.5) |
| Order of resection (%) | Combined | 14 (12.7) |
|  | Liver first | 3 (2.7) |
|  | Primary first | 89 (80.9) |
|  | NA | 4 (3.6) |
| Two-stage resection (%) |  | 3 (2.7) |
| Anatomical side of CRC = right sided/left sided (%) |  | 34/76 (30.9/69.1) |
| MSI status (%) | MSI-L | 75 (68.2) |
|  | MSI-H | 3 (2.7) |
|  | NA | 32 (29.1) |
| BRAF mutation status (%) | negative | 72 (65.5) |
|  | positive | 6 (5.5) |
|  | NA | 32 (29.1) |
| KRAS mutation status (%) | Wild type | 15 (13.6) |
|  | Mutated | 12 (10.9) |
|  | NA | 83 (75.5) |
| Elevated CEA prior to liver resection (%) | Not elevated | 25 (22.7) |
|  | Elevated | 83 (75.5) |
|  | NA | 2 (1.8) |
| CRLM anatomical distribution (%) | Unilateral | 85 (77.3) |
|  | Central | 3 (2.7) |
|  | Bilateral | 22 (20.0) |
| Diameter of largest CRLM (mm) (median [IQR]) |  | 30.00 [20.00, 50.00] |
| Number of CRLM (%) | 1 | 56 (50.9) |
|  | 2-3 | 38 (34.5) |
|  | >3 | 16 (14.5) |
| CRLM tumour differentiation (%) | Well | 8 (7.3) |
|  | Moderate | 78 (70.9) |
|  | Poor | 6 (5.5) |
|  | NA | 18 (16.4) |
| Chemotherapy prior to liver resection = Yes (%) |  | 82 (74.5) |
| Type of liver resection (%) | Anatomical | 57 (51.8) |
|  | Non-Anatomical | 49 (44.5) |
|  | Combined | 4 (3.6) |
| Liver resection complexity (%) | Low | 67 (60.9) |
|  | Intermediate | 37 (33.6) |
|  | High | 6 (5.5) |
| Estimated liver resection blood loss (ml) (median [IQR]) |  | 150.00 [50.00, 300.00] |
| Post liver resection complications (%) | None | 66 (60.0) |
|  | Minor | 26 (23.6) |
|  | Major | 17 (15.5) |
|  | Mortality | 1 (0.9) |
| Liver resection margin status = R0/R1 (%) |  | 99/11 (90.0/10.0) |
| Adjuvant chemotherapy (%) | Yes | 74 (67.3) |
|  | No | 35 (31.8) |
|  | NA | 1 (0.9) |

**Supplementary Table 3a** - Raw biomarker expression data for primary CRC and CRLM. All comparisons calculated on basis of complete cases, with ^ using Mann-Whitney U and $ comparing case primary tumours to matched first occurrence CRLM.

|  |  | **Primaries** | |  |  | **CRLM** |  |  |
| --- | --- | --- | --- | --- | --- | --- | --- | --- |
|  |  | **Control** | **Case** | **p ^** | **Effect size (95% CI)** |  | **p $** | **Effect size (95% CI)** |
|  | **n** | 110 | 110 |  |  | 124 |  |  |
| **CALD1 expression, tumour (%)** |  |  |  | **0.007** | **0.184 (0.05 - 0.3)** |  | 0.395 | 0.088 (0.006 - 0.27) |
| Absent |  | 70 (63.6) | 50 (45.5) |  |  | 49 (39.5) |  |  |
| Low |  | 29 (26.4) | 50 (45.5) |  |  | 61 (49.2) |  |  |
| Moderate |  | 7 (6.4) | 8 (7.3) |  |  | 5 (4.0) |  |  |
| NA |  | 4 (3.6) | 2 (1.8) |  |  | 9 (7.3) |  |  |
| **CALD1 expression, stroma (%)** |  |  |  | 0.767 | 0.02 (0.003 - 0.16) |  | **0.044** | **0.188 (0.02 - 0.37)** |
| Absent |  | 1 (0.9) | 0 (0.0) |  |  | 4 (3.2) |  |  |
| Low |  | 42 (38.2) | 41 (37.3) |  |  | 27 (21.8) |  |  |
| Moderate |  | 41 (37.3) | 45 (40.9) |  |  | 62 (50.0) |  |  |
| High |  | 22 (20.0) | 22 (20.0) |  |  | 27 (21.8) |  |  |
| NA |  | 4 (3.6) | 2 (1.8) |  |  | 4 (3.2) |  |  |
| **IGFBP7 expression, tumour (%)** |  |  |  | 0.096 | 0.115 (0.008 - 0.26) |  | 0.941 | 0.027 (0.002 - 0.22) |
| Absent |  | 28 (25.5) | 17 (15.5) |  |  | 18 (14.5) |  |  |
| Low |  | 53 (48.2) | 56 (50.9) |  |  | 70 (56.5) |  |  |
| Moderate |  | 23 (20.9) | 29 (26.4) |  |  | 24 (19.4) |  |  |
| High |  | 2 (1.8) | 2 (1.8) |  |  | 3 (2.4) |  |  |
| NA |  | 4 (3.6) | 6 (5.5) |  |  | 9 (7.3) | 0.695 |  |
| **IGFBP7 expression, stroma (%)** |  |  |  | 0.759 | 0.021 (0.002 - 0.16) |  |  | 0.041 (0.004 - 0.24) |
| Absent |  | 47 (42.7) | 45 (40.9) |  |  | 52 (41.9) |  |  |
| Low |  | 58 (52.7) | 56 (50.9) |  |  | 62 (50.0) |  |  |
| High |  | 1 (0.9) | 3 (2.7) |  |  | 4 (3.2) |  |  |
| NA |  | 4 (3.6) | 6 (5.5) |  |  | 6 (4.8) |  |  |
| **POSTN expression, tumour (%)** |  |  |  | **0.002** | **0.216 (0.08 - 0.34)** |  | **<0.001** | **0.364 (0.18 - 0.53)** |
| Absent |  | 18 (16.4) | 10 (9.1) |  |  | 26 (21.0) |  |  |
| Low |  | 55 (50.0) | 42 (38.2) |  |  | 64 (51.6) |  |  |
| Moderate |  | 26 (23.6) | 37 (33.6) |  |  | 20 (16.1) |  |  |
| High |  | 5 (4.5) | 14 (12.7) |  |  | 6 (4.8) |  |  |
| NA |  | 6 (5.5) | 7 (6.4) |  |  | 8 (6.5) |  |  |
| **POSTN expression, stroma (%)** |  |  |  | **0.014** | **0.17 (0.03 - 0.3)** |  | **<0.001** | **0.435 (0.26 - 0.59)** |
| Absent |  | 25 (22.7) | 11 (10.0) |  |  | 39 (31.5) |  |  |
| Low |  | 43 (39.1) | 44 (40.0) |  |  | 53 (42.7) |  |  |
| Moderate |  | 23 (20.9) | 32 (29.1) |  |  | 17 (13.7) |  |  |
| High |  | 13 (11.8) | 18 (16.4) |  |  | 8 (6.5) |  |  |
| NA |  | 6 (5.5) | 5 (4.5) |  |  | 7 (5.6) |  |  |
| **FAP expression, tumour (%)** |  |  |  | **0.003** | **0.211 (0.08 - 0.35)** |  | 0.271 | 0.117 (0.007 - 0.31) |
| Absent |  | 9 (8.2) | 6 (5.5) |  |  | 7 (5.6) |  |  |
| Low |  | 42 (38.2) | 25 (22.7) |  |  | 40 (32.3) |  |  |
| Moderate |  | 40 (36.4) | 43 (39.1) |  |  | 43 (34.7) |  |  |
| High |  | 13 (11.8) | 27 (24.5) |  |  | 25 (20.2) |  |  |
| NA |  | 6 (5.5) | 9 (8.2) |  |  | 9 (7.3) |  |  |
| **FAP expression, stroma (%)** |  |  |  | 0.346 | 0.066 (0.004 - 0.2) |  | **0.016** | **0.222 (0.04 - 0.41)** |
| Absent |  | 29 (26.4) | 27 (24.5) |  |  | 44 (35.5) |  |  |
| Low |  | 57 (51.8) | 47 (42.7) |  |  | 61 (49.2) |  |  |
| Moderate |  | 18 (16.4) | 26 (23.6) |  |  | 10 (8.1) |  |  |
| NA |  | 6 (5.5) | 10 (9.1) |  |  | 9 (7.3) |  |  |
| **TGF-b expression, tumour (%)** |  |  |  | 0.708 | 0.026 (0.003 - 0.17) |  | 0.067 | 0.175 (0.02 - 0.36) |
| Absent |  | 15 (13.6) | 14 (12.7) |  |  | 22 (17.7) |  |  |
| Low |  | 59 (53.6) | 67 (60.9) |  |  | 73 (58.9) |  |  |
| Moderate |  | 28 (25.5) | 23 (20.9) |  |  | 17 (13.7) |  |  |
| High |  | 1 (0.9) | 2 (1.8) |  |  | 2 (1.6) |  |  |
| NA |  | 7 (6.4) | 4 (3.6) |  |  | 10 (8.1) |  |  |
| **TGF-b expression, stroma (%)** |  |  |  | 0.951 | 0.004 (0.002 - 0.15) |  | **0.012** | **0.22 (0.04 - 0.4)** |
| Absent |  | 36 (32.7) | 40 (36.4) |  |  | 60 (48.4) |  |  |
| Low |  | 62 (56.4) | 56 (50.9) |  |  | 54 (43.5) |  |  |
| Moderate |  | 10 (9.1) | 14 (12.7) |  |  | 4 (3.2) |  |  |
| NA |  | 2 (1.8) | 0 (0.0) |  |  | 6 (4.8) | **0.02** | **0.209 (0.03 - 0.38)** |
| **pSMAD2 expression, tumour (%)** |  |  |  | 0.326 | 0.067 (0.003 - 0.2) |  |  |  |
| Absent |  | 9 (8.2) | 8 (7.3) |  |  | 15 (12.1) |  |  |
| Low |  | 40 (36.4) | 32 (29.1) |  |  | 41 (33.1) |  |  |
| Moderate |  | 39 (35.5) | 45 (40.9) |  |  | 37 (29.8) |  |  |
| High |  | 20 (18.2) | 22 (20.0) |  |  | 24 (19.4) |  |  |
| NA |  | 2 (1.8) | 3 (2.7) |  |  | 7 (5.6) |  |  |
| **pSMAD2 expression, stroma (%)** |  |  |  | 0.901 | 0.008 (0.002 - 0.15) |  | 0.305 | 0.101 (0 - 0.28) |
| Absent |  | 92 (83.6) | 91 (82.7) |  |  | 93 (75.0) |  |  |
| Low |  | 18 (16.4) | 17 (15.5) |  |  | 27 (21.8) |  |  |
| NA |  | 0 (0.0) | 2 (1.8) |  |  | 4 (3.2) |  |  |

**Supplementary Table 3b** – Output from the ‘Evaluate Cutpoints’ application for Binary transformation of primary CRC biomarkers. Note: pSMAD2 stroma only scored 0 and 1 on the original scale and therefore was binary from outset. se, standard error; sp, pooled sample standard deviation; PPV, positive predictive value; NPV, negative predictive value; DLR, diagnostic likelihood ratio

| Biomarker | Method | ‘Positive’ score (0 - 3) | se | sp | PPV | NPV | DLR.Positive | DLR.Negative |
| --- | --- | --- | --- | --- | --- | --- | --- | --- |
| CALD1 tumour | Youden | 2 – 3 | 0.537 | 0.66 | 0.617 | 0.583 | 1.581 | 0.701 |
| CALD1 stroma | Youden | 3 | 0.62 | 0.406 | 0.515 | 0.512 | 1.044 | 0.936 |
| IGFBP7 tumour | Youden | 1 - 3 | 0.837 | 0.264 | 0.527 | 0.622 | 1.137 | 0.619 |
| IGFBP7 stroma | Youden | 2 – 3 | 0.029 | 0.991 | 0.75 | 0.51 | 3.058 | 0.98 |
| POSTN tumour | Youden | 3 | 0.495 | 0.702 | 0.622 | 0.584 | 1.661 | 0.719 |
| POSTN stroma | Youden | 1 – 3 | 0.895 | 0.24 | 0.543 | 0.694 | 1.179 | 0.436 |
| FAP tumour | Youden | 3 | 0.693 | 0.49 | 0.569 | 0.622 | 1.36 | 0.626 |
| FAP stroma | Youden | 2 – 3 | 0.26 | 0.827 | 0.591 | 0.538 | 1.502 | 0.895 |
| TGF-b tumour | Youden | 1 – 3 | 0.868 | 0.146 | 0.511 | 0.517 | 1.016 | 0.907 |
| TGF-b stroma | Youden | 2 – 3 | 0.127 | 0.907 | 0.583 | 0.505 | 1.375 | 0.962 |
| pSMAD2 tumour | Youden | 3 | 0.626 | 0.454 | 0.532 | 0.551 | 1.146 | 0.824 |

**Supplementary Table 3c** – Output from the ‘Evaluate Cutpoints’ application for Binary transformation of CRLM biomarkers. Note: pSMAD2 stroma only scored 0 and 1 on the original scale and therefore was binary from outset. HR, Hazard ratio; 95% CI, 95% confidence interval

| Biomarker | ‘Positive’ score (0 - 3) | HR | 95% CI | P value |
| --- | --- | --- | --- | --- |
| CALD1 tumour | 2 – 3 | 0.786 | 0.178 - 3.48 | 0.751 |
| CALD1 stroma | 1 – 3 | 1.76 | 0.241 - 12.9 | 0.583 |
| IGFBP7 tumour | 1 – 3 | 1.64 | 0.465 - 5.79 | 0.438 |
| IGFBP7 stroma | 2 | 5.64 | 1.75 - 18.2 | 0.00109 |
| POSTN tumour | 1 – 3 | 1.05 | 0.413 - 2.6 | 0.914 |
| POSTN stroma | 3 | 2.07 | 0.732 – 5.82 | 0.161 |
| FAP tumour | 3 | 1.2 | 0.546 – 2.65 | 0.646 |
| FAP stroma | 2 – 3 | 1.88 | 0.711 - 4.98 | 0.196 |
| TGF-b tumour | 2 – 3 | 1.4 | 0.67 – 2.9 | 0.371 |
| TGF-b stroma | 2 – 3 | 2.33 | 0.55 – 9.86 | 0.237 |
| pSMAD2 tumour | 3 | 0.578 | 0.249 - 1.34 | 0.194 |

|  |  | **OR (univariable)** | **P** | **OR (multivariable)** | **P** |
| --- | --- | --- | --- | --- | --- |
| **Sex** | Female | 0.96 (0.56-1.66) | 0.890 |  |  |
| **Age at resection of primary CRC** |  | 1.01 (0.98-1.03) | 0.659 |  |  |
| **Year of resection** | 1998-2002 | 1.00 (reference) | 0.094 |  |  |
|  | 2003-2007 | 0.67 (0.24-1.87) |  |  |  |
|  | 2008-2012 | 0.42 (0.15-1.13) |  |  |  |
| **Anatomical side of CRC** | Left sided | 1.09 (0.61-1.92) |  |  |  |
| **Histological subtype** | Adenocarcinoma | 1.00 (reference) | 0.447 |  |  |
|  | Mucinous adenocarcinoma | 1.34 (0.34-5.17) |  |  |  |
|  | Other | *NA* |  |  |  |
| **Neoadjuvant treatment** | yes | 2.04 (0.36-11.47) |  |  |  |
| **Pathological stage** | I | 1.00 (reference) | 0.978 |  |  |
|  | IIa | 0.90 (0.31-2.62) |  |  |  |
|  | IIb | 0.77 (0.19-3.20) |  |  |  |
|  | IIc | 1.80 (0.14-23.72) |  |  |  |
|  | IIIa | 0.60 (0.15-2.38) |  |  |  |
|  | IIIb | 0.90 (0.33-2.45) |  |  |  |
|  | IIIc | 1.00 (0.33-3.03) |  |  |  |
| **Histological grade** | Low | 1.00 (reference) | 0.141 |  |  |
|  | Mod | 2.17 (0.98-4.82) |  |  |  |
|  | High | 0.97 (0.43-2.18) |  |  |  |
| **Pattern of growth** | Circumscribed | 1.00 (reference) | 0.275 |  |  |
|  | Infiltrative | 1.78 (0.28-11.31) |  |  |  |
|  | Irregular | *NA* |  |  |  |
|  | Pushing | 1.20 (0.19-7.72) |  |  |  |
| **Peritumoural lymphocytic response** | Present | 0.63 (0.34-1.17) | 0.141 |  |  |
|  | Present | 1.71 (0.91-3.21) | 0.093 |  |  |
| **Extramural venous permeation** | Present | 1.71 (0.83-3.53) | 0.147 |  |  |
| **Discontinuous extramural tumour nodules** | Present | 1.44 (0.67-3.09) | 0.342 |  |  |
| **Margin status** | R1 | 0.19 (0.02-1.70) | 0.137 |  |  |
| **MSI status** | MSI-H | 0.34 (0.10-1.10) | 0.071 |  |  |
| **BRAF V600E mutation** | Positive | 0.44 (0.18-1.10) | 0.078 |  |  |
| **CALD1 tumour** | Positive | 1.15 (0.40-3.32) | 0.789 |  |  |
| **CALD1 stroma** | Positive | 0.98 (0.50-1.90) | 0.942 |  |  |
| **IGFBP7 tumour** | Positive | 1.79 (0.92-3.51) | 0.087 |  |  |
| **IGFBP7 stroma** | Positive | 3.06 (0.31-30.23) | 0.338 |  |  |
| **POSTN tumour** | Positive | **3.06 (1.06-8.87)** | **0.039** |  |  |
| **POSTN stroma** | Positive | **2.54 (1.17-5.54)** | **0.019** | **2.49 (1.13-5.48)** | **0.023** |
| **FAP tumour** | Positive | **2.47 (1.19-5.12)** | **0.015** | **2.42 (1.15-5.07)** | **0.020** |
| **FAP stroma** | Positive | 1.56 (0.79-3.10) | 0.201 |  |  |
| **TGF-b tumour** | Positive | 1.08 (0.49-2.38) | 0.842 |  |  |
| **TGF-b stroma** | Positive | 1.39 (0.59-3.29) | 0.451 |  |  |
| **pSMAD2 tumour** | Positive | 1.12 (0.57-2.21) | 0.739 |  |  |
| **pSMAD2 stroma** | Positive | 0.94 (0.45-1.95) | 0.868 |  |  |

**Supplementary Table 4 -** Univariable and multivariable mixed effects model for developing CRLM after primary CRC resection. NA denotes estimate of 0.00 and confidence interval → inf. due to low event number. Values in parentheses are 95% confidence intervals (CI). Pooled AUC (95% CI) after 5-fold cross-validation repeated 5 times = 0.58 (0.50-0.65). OR, odds ratio.

|  |  | **Overall Survival** | | | |  | **Disease Free Survival** | | | |
| --- | --- | --- | --- | --- | --- | --- | --- | --- | --- | --- |
|  |  | **HR (univariable)** | **P** | **HR (multivariable)** | **P** |  | **HR (univariable)** | **P** | **HR (multivariable)** | **P** |
| **Sex** | Female | 0.63 (0.33-1.20) | 0.157 |  |  |  | **0.53 (0.32-0.89)** | **0.016** |  |  |
| **Timing of metastasis** | Metachronous | 0.65 (0.36-1.19) | 0.161 |  |  |  | 0.76 (0.48-1.21) | 0.254 |  |  |
| **Age at liver resection** |  | 1.02 (0.99-1.05) | 0.132 |  |  |  | 1.00 (0.98-1.02) | 0.977 |  |  |
| **Anatomtical side of CRC** | Left sided | 0.81 (0.43-1.51) | 0.500 |  |  |  | 1.55 (0.92-2.62) | 0.100 |  |  |
| **Histological subtype** | Adenocarcinoma | 1.00 (reference) | 0.438 |  |  |  | 1.00 (reference) | 0.242 |  |  |
|  | Mucinous | 0.71 (0.10-5.17) |  |  |  |  | 1.31 (0.41-4.17) |  |  |  |
|  | Other | *NA* |  |  |  |  | *NA* |  |  |  |
| **MSI status** | MSI-H | 2.14 (0.51-8.97) | 0.298 |  |  |  | 0.41 (0.06-3.01) | 0.383 |  |  |
| **BRAF V600E mutation positive** |  | 2.39 (0.85-6.70) | 0.098 | **2.99 (1.05-8.56)** | **0.041** |  | 1.01 (0.37-2.79) | 0.981 |  |  |
| **Elevated CEA** | Elevated | 1.63 (0.78-3.41) | 0.192 |  |  |  | 1.70 (0.94-3.06) | 0.078 | **1.91 (1.01-3.65)** | **0.047** |
| **Chemotherapy prior to liver resection** | Yes | 1.30 (0.64-2.64) | 0.462 |  |  |  | 1.56 (0.89-2.71) | 0.119 |  |  |
| **Adjuvant chemotherapy** | Yes | 1.78 (0.87-3.62) | 0.114 |  |  |  | **2.00 (1.15-3.45)** | **0.015** |  |  |
| **Diameter of largest CRLM (mm)** |  | 1.01 (1.00-1.02) | 0.299 |  |  |  | 1.01 (1.00-1.01) | 0.082 | **1.01 (1.00-1.02)** | **0.044** |
| **Number of CRLM** | 1 | 1.00 (reference) | 0.571 |  |  |  | **1.00 (reference)** | **0.001** | **1.00 (reference)** | **<0.001** |
|  | 2-3 | 1.03 (0.52-2.02) |  |  |  |  | **1.30 (0.77-2.18)** |  | **1.72 (0.95-3.12)** |  |
|  | >3 | 1.53 (0.70-3.34) |  |  |  |  | **3.63 (1.93-6.84)** |  | **5.75 (2.82-11.70)** |  |
| **Liver resection margin status** | R1 | **2.18 (1.01-4.70)** | **0.047** | **2.38 (1.09-5.19)** | **0.029** |  | **2.45 (1.25-4.81)** | **0.009** |  |  |
| **Estimated liver resection blood loss (ml)** |  | 1.00 (1.00-1.00) | 0.421 |  |  |  | 1.00 (1.00-1.00) | 0.221 |  |  |
| **Post liver resection complications** | None | 1.00 (reference) | 0.673 |  |  |  | 1.00 (reference) | 0.220 | **1.00 (reference)** | **0.021** |
|  | Minor | 1.08 (0.53-2.19) |  |  |  |  | 1.55 (0.91-2.65) |  | **1.82 (1.02-3.23)** |  |
|  | Major | 1.49 (0.64-3.43) |  |  |  |  | 1.46 (0.77-2.79) |  | **2.74 (1.27-5.92)** |  |
| **CRLM tumour differentiation** | Well | 1.00 (reference) | 0.692 |  |  |  | 1.00 (reference) | 0.775 |  |  |
|  | Moderate | 1.23 (0.38-4.03) |  |  |  |  | 1.22 (0.49-3.06) |  |  |  |
|  | Poor | 2.80 (0.56-14.05) |  |  |  |  | 1.56 (0.46-5.28) |  |  |  |
| **Liver resection complexity** | Low | 1.00 (reference) | 0.495 |  |  |  | 1.00 (reference) | 0.357 |  |  |
|  | Intermediate | 1.36 (0.73-2.53) |  |  |  |  | 1.01 (0.61-1.67) |  |  |  |
|  | High | 1.76 (0.53-5.88) |  |  |  |  | 1.97 (0.83-4.68) |  |  |  |
| **Primary CRC lymph node metastases** | Yes | 1.45 (0.77-2.74) | 0.252 |  |  |  | **1.60 (0.99-2.60)** | **0.056** | **1.86 (1.09-3.20)** | **0.023** |
| **CALD1 tumour** | Positive | 0.73 (0.18-3.06) | 0.670 |  |  |  | 1.00 (0.36-2.76) | 0.994 |  |  |
| **CALD1 stroma** | Positive | 1.75 (0.24-12.70) | 0.582 |  |  |  | 3.85 (0.53-27.81 | 0.181 |  |  |
| **IGFBP7 tumour** | Positive | 1.78 (0.55-5.76) | 0.335 |  |  |  | 1.03 (0.45-2.35) | 0.950 |  |  |
| **IGFBP7 stroma** | Positive | **4.24 (1.49-12.04)** | **0.007** | **4.84 (1.69-13.90)** | **0.003** |  | **4.40 (1.57-12.32** | **0.005** | **4.20 (1.39-12.7)** | **0.011** |
| **POSTN tumour** | Positive | 1.26 (0.56-2.84) | 0.572 |  |  |  | 1.04 (0.57-1.91) | 0.891 |  |  |
| **POSTN stroma** | Positive | 2.09 (0.74-5.85) | 0.162 |  |  |  | **3.62 (1.17-11.20** | **0.026** | **3.57 (1.23-10.38)** | **0.019** |
| **FAP tumour** | Positive | 1.11 (0.53-2.31) | 0.789 |  |  |  | 0.91 (0.49-1.72) | 0.781 |  |  |
| **FAP stroma** | Positive | 1.66 (0.70-3.94) | 0.253 |  |  |  | 1.19 (0.55-2.60) | 0.662 |  |  |
| **TGF-b tumour** | Positive | 1.44 (0.69-3.01) | 0.330 |  |  |  | 0.90 (0.46-1.79) | 0.772 |  |  |
| **TGF-b stroma** | Positive | 2.44 (0.57-10.35) | 0.226 |  |  |  | 0.97 (0.24-3.95) | 0.961 |  |  |
| **pSMAD2 tumour** | Positive | 0.66 (0.30-1.49) | 0.320 |  |  |  | 0.92 (0.49-1.71) | 0.784 |  |  |
| **pSMAD2 stroma** | Positive | 0.73 (0.32-1.63) | 0.438 |  |  |  | 1.29 (0.74-2.23) | 0.372 |  |  |

**Supplementary Table 5 -** Univariable and multivariable Cox proportional hazards models of overall survival and disease-free survival following CRLM resection. Number in models = 109. NA denotes estimate of 0.00 and confidence interval → inf. due to low event number. Values in parentheses are 95% confidence intervals (CI). Pooled C-statistic (95% CI) after 1000 bootstrapped simulations = 0.572 (0.571-0.572) and 0.685 (0.684-0.687) for overall and disease-free survival models, respectively. HR, hazard ratio.

**Supplementary Table 6 –** Full list of DGE significant after Bonferroni correction for case primary CRC stroma versus control primary CRC stroma, CRLM stroma versus paired primary stroma and CRLM tumour versus paired primary tumour. std.error, standard error; FDR, false discovery rate.

| **Comparison** | **Gene** | **Accession Number** | **estimate** | **std.error** | **statistic** | **p.value** | **FDR** | **bonferroni** |
| --- | --- | --- | --- | --- | --- | --- | --- | --- |
| **Case primary CRC stroma *versus* control primary CRC stroma** | RELN | NM_022136.3 | -1.9861503 | 0.31527877 | -6.2996639 | 1.34E-09 | 1.03E-06 | 1.03E-06 |
|  | MYH11 | NM_000269.2 | -1.9342969 | 0.3735733 | -5.1778241 | 8.80E-07 | 0.00033862 | 0.00067724 |
|  | ZKSCAN5 | NM_014569.3 | -1.2777301 | 0.26034006 | -4.9079274 | 4.30E-06 | 0.00110259 | 0.00330777 |
|  | SPARCL1 | NM_002353.2 | -1.2660293 | 0.2672014 | -4.7381088 | 8.73E-06 | 0.001547 | 0.00672425 |
|  | AHNAK | NM_005100.3 | -1.0205159 | 0.21812399 | -4.6786046 | 1.23E-05 | 0.001547 | 0.00947047 |
|  | FXYD6 | NM_000581.2 | -1.1216718 | 0.24146621 | -4.6452538 | 1.31E-05 | 0.001547 | 0.01004929 |
|  | ABI3BP | NM_015429.3 | -1.5703555 | 0.34059963 | -4.6105614 | 1.52E-05 | 0.001547 | 0.01169753 |
|  | HSPG2 | NM_001562.2 | -0.7878529 | 0.16994696 | -4.6358752 | 1.61E-05 | 0.001547 | 0.01237597 |
|  | THBS4 | NM_022144.2 | -2.254283 | 0.4945787 | -4.5579864 | 1.97E-05 | 0.00168177 | 0.01517938 |
|  | PTPRB | NM_014248.2 | -1.1905397 | 0.26235689 | -4.5378633 | 2.18E-05 | 0.00168177 | 0.01681766 |
|  | PLA2G2D | NM_014225.3 | -1.62384 | 0.36471603 | -4.4523406 | 3.42E-05 | 0.002395 | 0.02634498 |
|  | MYLK | NM_005009.2 | -1.0597928 | 0.23986778 | -4.4182374 | 4.05E-05 | 0.00259995 | 0.03119939 |
|  | PRF1 | NM_012232.5 | -1.0881417 | 0.24926036 | -4.3654825 | 5.15E-05 | 0.00282807 | 0.03965782 |
|  | FBLN5 | NM_006682.2 | -1.2522618 | 0.28711648 | -4.3615116 | 5.19E-05 | 0.00282807 | 0.03994772 |
|  | ITM2A | NM_002276.4 | -1.4954578 | 0.34421957 | -4.344488 | 5.51E-05 | 0.00282807 | 0.04242099 |
|  | CCL5 | NM_001123041.2 | -1.5533792 | 0.36014633 | -4.3131891 | 6.08E-05 | 0.00292814 | 0.04685028 |
|  |  |  |  |  |  |  |  |  |
| **CRLM stroma *versus* paired primary CRC stroma** | AAMP | NM_001087.3 | 4.81353017 | 0.3005681 | 16.0147742 | 0 | 0 | 0 |
|  | ABI3BP | NM_015429.3 | 4.79600516 | 0.56465216 | 8.49373378 | 0 | 0 | 0 |
|  | ACHE | NM_000665.3 | 3.49183949 | 0.4909283 | 7.11272813 | 5.54E-12 | 7.13E-12 | 4.27E-09 |
|  | ACTG2 | NM_001615.3 | 4.21560372 | 0.67803669 | 6.21736817 | 2.24E-09 | 2.65E-09 | 1.73E-06 |
|  | ACVR1 | NM_001105.2 | 5.09163051 | 0.32841977 | 15.5034225 | 0 | 0 | 0 |
|  | ACVR1C | NM_145259.2 | 3.51163112 | 0.60128057 | 5.84025376 | 1.93E-08 | 2.21E-08 | 1.48E-05 |
|  | ACVRL1 | NM_000020.1 | 4.73520668 | 0.38533783 | 12.2884553 | 0 | 0 | 0 |
|  | ADAM15 | NM_207195.1 | 5.19494576 | 0.28088401 | 18.4949856 | 0 | 0 | 0 |
|  | ADAM17 | NM_003183.4 | 5.20305575 | 0.34888467 | 14.9133975 | 0 | 0 | 0 |
|  | ADAM28 | NM_014265.4 | 4.27280686 | 0.62007377 | 6.89080411 | 1.82E-11 | 2.29E-11 | 1.40E-08 |
|  | ADAM8 | NM_001109.4 | 4.46004511 | 0.5729514 | 7.78433404 | 2.29E-14 | 3.20E-14 | 1.76E-11 |
|  | ADAM9 | NM_001005845.1 | 5.37200805 | 0.3495174 | 15.3697872 | 0 | 0 | 0 |
|  | ADAMTS1 | NM_006988.3 | 5.78177147 | 0.40461886 | 14.2894265 | 0 | 0 | 0 |
|  | ADAMTS12 | NM_030955.2 | 5.15012342 | 0.44860807 | 11.4802292 | 0 | 0 | 0 |
|  | ADD1 | NM_001119.4 | 5.27790525 | 0.34003416 | 15.5216914 | 0 | 0 | 0 |
|  | ADM2 | NM_001253845.1 | 3.76704194 | 0.47826361 | 7.8764971 | 1.34E-14 | 1.89E-14 | 1.03E-11 |
|  | ADRA2B | NM_000682.4 | 2.74070091 | 0.57703751 | 4.74960612 | 7.99E-06 | 8.60E-06 | 0.00615104 |
|  | AEBP1 | NM_001129.3 | 7.84976161 | 0.42445119 | 18.493909 | 0 | 0 | 0 |
|  | AGGF1 | NM_018046.3 | 3.58965097 | 0.38675196 | 9.28153264 | 0 | 0 | 0 |
|  | AGK | NM_018238.3 | 4.17767425 | 0.41864974 | 9.97892466 | 0 | 0 | 0 |
|  | AGR2 | NM_006408.3 | 3.91349173 | 0.65067887 | 6.01447492 | 6.48E-09 | 7.50E-09 | 4.99E-06 |
| *(cont. from previous page)*  **CRLM stroma *versus* paired primary CRC stroma** | AGRN | NM_198576.2 | 5.8959012 | 0.29585659 | 19.9282404 | 0 | 0 | 0 |
|  | AGT | NM_000029.3 | 5.27082148 | 0.69207939 | 7.61592032 | 8.59E-14 | 1.17E-13 | 6.62E-11 |
|  | AHNAK | NM_001620.2 | 6.3910698 | 0.36447464 | 17.5350192 | 0 | 0 | 0 |
|  | AKAP12 | NM_005100.3 | 4.16451017 | 0.41010552 | 10.1547283 | 0 | 0 | 0 |
|  | PALM2 | NM_001004065.4 | 5.61512316 | 0.38998187 | 14.3984211 | 0 | 0 | 0 |
|  | AKT1 | NM_005163.2 | 6.31341968 | 0.28046252 | 22.5107431 | 0 | 0 | 0 |
|  | AKT2 | NM_001626.2 | 5.18546072 | 0.36388538 | 14.2502585 | 0 | 0 | 0 |
|  | AKT3 | NM_005465.4 | 4.78198653 | 0.50960535 | 9.38370542 | 0 | 0 | 0 |
|  | ALB | NM_000477.5 | 8.65448877 | 1.04293121 | 8.2982355 | 2.22E-16 | 3.27E-16 | 1.71E-13 |
|  | ALDOA | NM_184041.2 | 7.17986757 | 0.35324281 | 20.3255875 | 0 | 0 | 0 |
|  | ALOX5 | NM_000698.2 | 6.01352342 | 0.47408526 | 12.6844767 | 0 | 0 | 0 |
|  | AMH | NM_000479.3 | 3.83466247 | 0.72720592 | 5.27314528 | 4.92E-07 | 5.41E-07 | 0.00037899 |
|  | AMMECR1L | NM_001199140.1 | 4.93763764 | 0.38292776 | 12.8944363 | 0 | 0 | 0 |
|  | ANG | NM_001145.4 | 5.08929193 | 0.5668228 | 8.97862952 | 0 | 0 | 0 |
|  | ANGPT1 | NM_001146.3 | 4.3071906 | 0.57180941 | 7.53256336 | 1.86E-13 | 2.51E-13 | 1.43E-10 |
|  | ANGPT2 | NM_001147.2 | 4.93140183 | 0.48925535 | 10.0794029 | 0 | 0 | 0 |
|  | ANGPTL2 | NM_012098.2 | 4.7299508 | 0.41669896 | 11.3510022 | 0 | 0 | 0 |
|  | ANGPTL4 | NR_104213.1 | 4.09669781 | 0.78543733 | 5.21581755 | 7.27E-07 | 7.94E-07 | 0.00055975 |
|  | ANPEP | NM_001150.1 | 5.47420649 | 0.6000147 | 9.12345401 | 0 | 0 | 0 |
|  | ANXA2P2 | NR_003573.1 | 7.69038593 | 0.30091277 | 25.556861 | 0 | 0 | 0 |
|  | AP1M2 | NM_005498.4 | 3.00608802 | 0.51477285 | 5.83963983 | 2.04E-08 | 2.33E-08 | 1.57E-05 |
|  | APC | NM_000038.3 | 4.27437547 | 0.35194979 | 12.1448444 | 0 | 0 | 0 |
|  | APOD | NM_001647.3 | 3.04794047 | 0.66121771 | 4.6095869 | 1.58E-05 | 1.69E-05 | 0.01217174 |
|  | APOE | NM_000041.2 | 8.45533368 | 0.71030042 | 11.9038838 | 0 | 0 | 0 |
|  | AQP1 | NM_198098.1 | 4.33613223 | 0.53331974 | 8.13045519 | 2.00E-15 | 2.89E-15 | 1.54E-12 |
|  | ARAP2 | NM_015230.2 | 3.6692738 | 0.46811903 | 7.83833506 | 2.07E-14 | 2.89E-14 | 1.59E-11 |
|  | AREG | NM_001657.2 | 3.92985018 | 0.81712525 | 4.80936084 | 5.89E-06 | 6.36E-06 | 0.00453897 |
|  | ARHGDIB | NM_001175.4 | 7.22440675 | 0.59407154 | 12.1608363 | 0 | 0 | 0 |
|  | ASPN | NM_017680.3 | 6.07941138 | 0.64125135 | 9.48054361 | 0 | 0 | 0 |
|  | ATPIF1 | NM_178190.2 | 4.6962656 | 0.35491066 | 13.2322472 | 0 | 0 | 0 |
|  | B3GNT3 | NM_014256.3 | 5.13789801 | 0.49022201 | 10.4807575 | 0 | 0 | 0 |
|  | BAD | NM_004322.3 | 4.32484341 | 0.35593965 | 12.1504965 | 0 | 0 | 0 |
|  | BAG2 | NM_004282.3 | 4.21717953 | 0.45060252 | 9.35897894 | 0 | 0 | 0 |
|  | BAI1 | NM_001702.1 | 4.18469882 | 0.5842911 | 7.16201025 | 2.72E-12 | 3.54E-12 | 2.09E-09 |
|  | BAI3 | NM_001704.1 | 3.96431146 | 0.55189639 | 7.18307192 | 2.34E-12 | 3.06E-12 | 1.80E-09 |
|  | BCAS1 | NM_003657.2 | 3.01194133 | 0.65505898 | 4.59796966 | 1.70E-05 | 1.81E-05 | 0.01308065 |
|  | BICC1 | NM_001080512.1 | 3.20762334 | 0.49856792 | 6.43367379 | 4.58E-10 | 5.48E-10 | 3.52E-07 |
|  | BMP4 | NM_001202.3 | 4.08742631 | 0.45874227 | 8.91007135 | 0 | 0 | 0 |
|  | BMP5 | NM_021073.2 | 4.05925918 | 0.66323099 | 6.12043052 | 3.73E-09 | 4.37E-09 | 2.87E-06 |
|  | BMPR1A | NM_004329.2 | 5.5019575 | 0.34893824 | 15.7677117 | 0 | 0 | 0 |
|  | BMPR1B | NM_001203.1 | 3.51304777 | 0.54008954 | 6.50456551 | 3.11E-10 | 3.75E-10 | 2.39E-07 |
| *(cont. from previous page)*  **CRLM stroma *versus* paired primary CRC stroma** | BMPR2 | NM_001204.5 | 5.1032304 | 0.31222006 | 16.3449793 | 0 | 0 | 0 |
|  | BNC2 | NM_017637.5 | 3.28912943 | 0.61439791 | 5.353419 | 3.30E-07 | 3.63E-07 | 0.00025426 |
|  | BRMS1 | NM_015399.3 | 4.10193423 | 0.39781038 | 10.31128 | 0 | 0 | 0 |
|  | BTG1 | NM_001731.2 | 6.43142748 | 0.3872108 | 16.6096283 | 0 | 0 | 0 |
|  | C1S | NM_001734.2 | 5.62288452 | 0.44305547 | 12.6911524 | 0 | 0 | 0 |
|  | C3 | NM_000064.2 | 5.03255484 | 0.67770284 | 7.42590194 | 3.63E-13 | 4.83E-13 | 2.80E-10 |
|  | C3AR1 | NM_004054.2 | 5.10068796 | 0.43715873 | 11.6678169 | 0 | 0 | 0 |
|  | CADM1 | NM_014333.3 | 4.62509209 | 0.53572397 | 8.63334914 | 3.33E-16 | 4.88E-16 | 2.56E-13 |
|  | CALCRL | NM_005795.3 | 5.73782241 | 0.46050041 | 12.4599725 | 0 | 0 | 0 |
|  | CALD1 | NM_004342.6 | 7.07456721 | 0.38622662 | 18.3171402 | 0 | 0 | 0 |
|  | CAMK2A | NM_171825.1 | 3.88903928 | 0.54466401 | 7.14025378 | 3.53E-12 | 4.56E-12 | 2.72E-09 |
|  | CAMK2D | NM_172127.1 | 5.34189823 | 0.31233038 | 17.103358 | 0 | 0 | 0 |
|  | cyclic-AMP | NM_004345.3 | 3.81260984 | 0.6311188 | 6.04103353 | 5.19E-09 | 6.05E-09 | 3.99E-06 |
|  | CASP8 | NM_001228.4 | 4.83122706 | 0.3615189 | 13.3636916 | 0 | 0 | 0 |
|  | CAV1 | NM_001753.3 | 3.86071558 | 0.46846298 | 8.24123952 | 6.66E-16 | 9.73E-16 | 5.13E-13 |
|  | CC2D1B | NM_032449.2 | 3.75784583 | 0.4598015 | 8.17275689 | 1.11E-15 | 1.62E-15 | 8.55E-13 |
|  | CCBE1 | NM_133459.3 | 4.7880621 | 0.3245522 | 14.7528261 | 0 | 0 | 0 |
|  | CCDC80 | NM_199511.1 | 7.76803724 | 0.46169985 | 16.8248642 | 0 | 0 | 0 |
|  | CCL11 | NM_002986.2 | 4.1723332 | 0.6349616 | 6.57100089 | 2.16E-10 | 2.61E-10 | 1.66E-07 |
|  | CCL21 | NM_002989.2 | 5.88734975 | 0.69495015 | 8.47161449 | 5.55E-16 | 8.13E-16 | 4.27E-13 |
|  | CCL5 | NM_002985.2 | 5.71236105 | 0.6098408 | 9.36697091 | 0 | 0 | 0 |
|  | CCL8 | NM_005623.2 | 3.46675701 | 0.62464381 | 5.54997418 | 1.17E-07 | 1.31E-07 | 9.04E-05 |
|  | CCR2 | NM_001123041.2 | 4.58442001 | 0.57813152 | 7.92971812 | 9.66E-15 | 1.37E-14 | 7.44E-12 |
|  | CCR3 | NM_001837.2 | 3.30639471 | 0.59070998 | 5.5973233 | 8.44E-08 | 9.48E-08 | 6.50E-05 |
|  | CD163 | NM_004244.4 | 6.01215304 | 0.47373308 | 12.6910137 | 0 | 0 | 0 |
|  | CD24 | NM_013230.2 | 6.05948574 | 0.59158596 | 10.2427815 | 0 | 0 | 0 |
|  | CD2AP | NM_012120.2 | 5.01162297 | 0.32488753 | 15.4257168 | 0 | 0 | 0 |
|  | CD34 | NM_001773.2 | 4.69148439 | 0.42822051 | 10.9557676 | 0 | 0 | 0 |
|  | CD36 | NM_000072.3 | 4.42448602 | 0.42543345 | 10.3999485 | 0 | 0 | 0 |
|  | CD44 | NM_001001392.1 | 6.9125837 | 0.33990589 | 20.3367575 | 0 | 0 | 0 |
|  | CD46 | NM_172350.1 | 5.75623859 | 0.41988025 | 13.7092386 | 0 | 0 | 0 |
|  | CD82 | NM_002231.3 | 4.7136905 | 0.37794137 | 12.4720151 | 0 | 0 | 0 |
|  | CDC42 | NM_001039802.1 | 5.90218161 | 0.3232983 | 18.256148 | 0 | 0 | 0 |
|  | CDH1 | NM_004360.2 | 5.07110376 | 0.54378472 | 9.32557229 | 0 | 0 | 0 |
|  | CDH11 | NM_001797.2 | 6.02721252 | 0.42187506 | 14.2867241 | 0 | 0 | 0 |
|  | CDH13 | NM_001220488.1 | 4.3689966 | 0.49967303 | 8.74371099 | 0 | 0 | 0 |
|  | CDH2 | NM_001792.3 | 5.70375579 | 0.560689 | 10.1727622 | 0 | 0 | 0 |
|  | CDKN1A | NM_000389.2 | 5.14291538 | 0.40615465 | 12.662456 | 0 | 0 | 0 |
|  | CDKN2A | NM_000077.3 | 3.06786142 | 0.63656069 | 4.81943273 | 5.63E-06 | 6.08E-06 | 0.00433703 |
|  | CDS1 | NM_001263.3 | 3.78632832 | 0.43661816 | 8.67194413 | 0 | 0 | 0 |
|  | CEACAM1 | NM_001712.3 | 3.75367866 | 0.4945053 | 7.59077534 | 1.07E-13 | 1.46E-13 | 8.28E-11 |
| *(cont. from previous page)*  **CRLM stroma *versus* paired primary CRC stroma** | CEACAM5 | NM_004363.2 | 5.99840584 | 0.63326669 | 9.47216388 | 0 | 0 | 0 |
|  | CEACAM6 | NM_002483.4 | 5.76311125 | 0.73245337 | 7.86822955 | 5.83E-14 | 8.04E-14 | 4.49E-11 |
|  | CEP170 | NM_001042404.1 | 5.63930745 | 0.39691739 | 14.2077612 | 0 | 0 | 0 |
|  | CFP | NM_002621.2 | 3.86746073 | 0.63896478 | 6.05269784 | 5.68E-09 | 6.62E-09 | 4.37E-06 |
|  | CGN | NM_020770.2 | 3.56722461 | 0.3937023 | 9.06071566 | 0 | 0 | 0 |
|  | CHAD | NM_001267.2 | 3.50218399 | 0.51776576 | 6.76403172 | 4.70E-11 | 5.86E-11 | 3.62E-08 |
|  | CHD4 | NM_001273.2 | 5.23354977 | 0.36390657 | 14.3815755 | 0 | 0 | 0 |
|  | CHI3L1 | NM_001276.2 | 4.58121194 | 0.54133472 | 8.46280828 | 0 | 0 | 0 |
|  | CHORDC1 | XM_005254140.1 | 4.66285061 | 0.28973494 | 16.0935046 | 0 | 0 | 0 |
|  | CHRDL1 | NM_001143981.1 | 3.10310743 | 0.69993541 | 4.43341969 | 3.67E-05 | 3.90E-05 | 0.02827198 |
|  | CHRNA7 | NR_046324.1 | 4.36202577 | 0.49134078 | 8.87780119 | 0 | 0 | 0 |
|  | CKMT1A | NM_001015001.1 | 4.77561977 | 0.5287639 | 9.03166755 | 0 | 0 | 0 |
|  | CLDN1 | NM_021101.3 | 4.80338751 | 0.47932103 | 10.0212325 | 0 | 0 | 0 |
|  | CLDN3 | NM_001306.3 | 4.11928177 | 0.48766752 | 8.44690613 | 1.11E-16 | 1.64E-16 | 8.55E-14 |
|  | CLDN4 | NM_001305.3 | 5.1503813 | 0.48882243 | 10.5363031 | 0 | 0 | 0 |
|  | CLDN7 | NM_001307.3 | 5.02191775 | 0.46905187 | 10.7065296 | 0 | 0 | 0 |
|  | CLEC2B | NM_005127.2 | 6.33573306 | 0.40291534 | 15.7247254 | 0 | 0 | 0 |
|  | CLEC3B | NM_003278.2 | 3.89371919 | 0.46562058 | 8.36242939 | 2.22E-16 | 3.27E-16 | 1.71E-13 |
|  | CLIC4 | NM_013943.2 | 6.09922624 | 0.42180496 | 14.4598257 | 0 | 0 | 0 |
|  | CLU | NM_203339.2 | 4.76438277 | 0.54769529 | 8.69896607 | 0 | 0 | 0 |
|  | CNN1 | NM_001299.4 | 3.25521197 | 0.5253321 | 6.19648403 | 2.00E-09 | 2.36E-09 | 1.54E-06 |
|  | CNOT10 | NM_001256741.1 | 3.89572351 | 0.44152026 | 8.82343094 | 0 | 0 | 0 |
|  | CNOT4 | NM_001190848.1 | 4.5905728 | 0.34706535 | 13.2268253 | 0 | 0 | 0 |
|  | COG7 | NM_153603.3 | 4.43665747 | 0.39211805 | 11.3145965 | 0 | 0 | 0 |
|  | COL18A1 | NM_030582.3 | 6.63053998 | 0.38444011 | 17.2472639 | 0 | 0 | 0 |
|  | COL1A1 | NM_000088.3 | 9.78641411 | 0.40782748 | 23.9964558 | 0 | 0 | 0 |
|  | COL1A2 | NM_000089.3 | 8.96238527 | 0.40447189 | 22.1582402 | 0 | 0 | 0 |
|  | COL3A1 | NM_000090.3 | 9.71585019 | 0.43328679 | 22.4236015 | 0 | 0 | 0 |
|  | COL4A1 | NM_001845.4 | 9.34477777 | 0.38820853 | 24.0715417 | 0 | 0 | 0 |
|  | COL4A2 | NM_001846.2 | 8.79859537 | 0.37981221 | 23.1656467 | 0 | 0 | 0 |
|  | COL5A1 | NM_000093.3 | 7.94131696 | 0.38888881 | 20.4205336 | 0 | 0 | 0 |
|  | COL5A2 | NM_000393.3 | 7.01176916 | 0.3438188 | 20.3937919 | 0 | 0 | 0 |
|  | COL6A1 | NM_001848.2 | 5.24489456 | 0.47608735 | 11.0166643 | 0 | 0 | 0 |
|  | COL6A2 | NM_001849.2 | 6.38614124 | 0.39699897 | 16.08604 | 0 | 0 | 0 |
|  | COL6A3 | NM_004369.3 | 7.17101234 | 0.38217417 | 18.7637287 | 0 | 0 | 0 |
|  | COL7A1 | NM_000094.2 | 4.92827805 | 0.3948096 | 12.4826702 | 0 | 0 | 0 |
|  | COMP | NM_000095.2 | 5.44640509 | 0.63215074 | 8.61567458 | 0 | 0 | 0 |
|  | CREBBP | NM_001079846.1 | 4.86849926 | 0.31961107 | 15.2325741 | 0 | 0 | 0 |
|  | CRISPLD2 | NM_031476.3 | 5.79642379 | 0.44000451 | 13.1735554 | 0 | 0 | 0 |
|  | CSF2RB | NM_000395.2 | 4.00814631 | 0.59294987 | 6.75967144 | 6.56E-11 | 8.13E-11 | 5.05E-08 |
|  | CSPG4 | NM_001897.4 | 4.86940758 | 0.45301408 | 10.7489101 | 0 | 0 | 0 |
| *(cont. from previous page)*  **CRLM stroma *versus* paired primary CRC stroma** | CST7 | NM_003650.3 | 3.67425521 | 0.60442412 | 6.07893551 | 5.17E-09 | 6.04E-09 | 3.98E-06 |
|  | CTNNB1 | NM_001098210.1 | 6.82929012 | 0.39230267 | 17.4082172 | 0 | 0 | 0 |
|  | CTNND1 | NM_001331.2 | 5.54722839 | 0.31764334 | 17.4637014 | 0 | 0 | 0 |
|  | CTSG | NM_001911.2 | 3.34838543 | 0.5830371 | 5.74300575 | 3.49E-08 | 3.96E-08 | 2.68E-05 |
|  | CTSH | NM_148979.2 | 5.71992181 | 0.42381205 | 13.4963644 | 0 | 0 | 0 |
|  | CTSK | NM_000396.2 | 5.31643082 | 0.45070353 | 11.7958491 | 0 | 0 | 0 |
|  | CTSL1 | NM_001912.4 | 5.90190978 | 0.54246495 | 10.8797993 | 0 | 0 | 0 |
|  | CUL1 | NM_003592.2 | 4.67324742 | 0.36066317 | 12.9573737 | 0 | 0 | 0 |
|  | CX3CL1 | NM_002996.3 | 3.5339291 | 0.62683396 | 5.63774352 | 7.33E-08 | 8.24E-08 | 5.64E-05 |
|  | CXADR | NM_001338.3 | 5.08852222 | 0.48469191 | 10.4984673 | 0 | 0 | 0 |
|  | CXCL10 | NM_001565.1 | 4.22631158 | 0.56945514 | 7.42167601 | 3.77E-13 | 5.01E-13 | 2.90E-10 |
|  | CXCL11 | NM_005409.3 | 3.89529889 | 0.58281609 | 6.68358161 | 8.91E-11 | 1.10E-10 | 6.86E-08 |
|  | CXCL12 | NM_199168.3 | 5.00312618 | 0.51367325 | 9.73990013 | 0 | 0 | 0 |
|  | CXCL17 | NM_198477.1 | 3.69718482 | 0.61341668 | 6.02719971 | 6.08E-09 | 7.06E-09 | 4.68E-06 |
|  | IL8 | NM_000584.2 | 6.5141645 | 0.55659289 | 11.7036431 | 0 | 0 | 0 |
|  | IL8RB | NM_001168298.1 | 4.87582863 | 0.61794668 | 7.89037117 | 1.09E-14 | 1.54E-14 | 8.38E-12 |
|  | CXCR3 | NM_001504.1 | 3.81026294 | 0.47327867 | 8.05078105 | 4.22E-15 | 6.04E-15 | 3.25E-12 |
|  | CXCR4 | NM_003467.2 | 7.67421031 | 0.53248059 | 14.4121879 | 0 | 0 | 0 |
|  | CYB561 | NM_001915.3 | 5.0361883 | 0.37507891 | 13.42701 | 0 | 0 | 0 |
|  | CYBB | NM_000397.3 | 6.01899393 | 0.46879948 | 12.839165 | 0 | 0 | 0 |
|  | CYP1B1 | NM_000104.3 | 6.634121 | 0.56873476 | 11.6647012 | 0 | 0 | 0 |
|  | DAG1 | NM_001165928.2 | 4.0756997 | 0.36403418 | 11.1959258 | 0 | 0 | 0 |
|  | DCN | NM_001920.3 | 7.05904493 | 0.44121473 | 15.9991144 | 0 | 0 | 0 |
|  | DDR2 | NM_006182.2 | 4.67856921 | 0.44933961 | 10.4121005 | 0 | 0 | 0 |
|  | DDX50 | NM_024045.1 | 4.82803636 | 0.40169227 | 12.0192414 | 0 | 0 | 0 |
|  | DENND5A | NM_015213.2 | 4.818967 | 0.34846472 | 13.8291388 | 0 | 0 | 0 |
|  | DENR | NM_003677.3 | 4.69037791 | 0.39214355 | 11.9608697 | 0 | 0 | 0 |
|  | DHX16 | NM_001164239.1 | 4.19134393 | 0.31576513 | 13.2736124 | 0 | 0 | 0 |
|  | DICER1 | NM_177438.2 | 5.03595018 | 0.39678254 | 12.6919652 | 0 | 0 | 0 |
|  | DLC1 | NM_006094.3 | 4.68685989 | 0.46769924 | 10.0210979 | 0 | 0 | 0 |
|  | DLG1 | NM_001098424.1 | 4.79072414 | 0.33205098 | 14.4276767 | 0 | 0 | 0 |
|  | DLL4 | NM_019074.2 | 5.02882831 | 0.40483554 | 12.4219043 | 0 | 0 | 0 |
|  | DNAJC14 | NM_032364.5 | 3.98257776 | 0.39486772 | 10.0858529 | 0 | 0 | 0 |
|  | DPT | NM_001937.3 | 4.34698994 | 0.5927226 | 7.33393661 | 7.68E-13 | 1.01E-12 | 5.91E-10 |
|  | DPYSL3 | NM_001387.2 | 6.17720679 | 0.4250877 | 14.5316056 | 0 | 0 | 0 |
|  | DSC2 | NM_024422.3 | 5.23619857 | 0.36359643 | 14.4011277 | 0 | 0 | 0 |
|  | ECM1 | NM_004425.3 | 4.91528205 | 0.32231854 | 15.2497652 | 0 | 0 | 0 |
|  | ECM2 | NM_001393.2 | 4.74600152 | 0.49189814 | 9.64834205 | 0 | 0 | 0 |
|  | ECSCR | NM_001077693.3 | 3.73482191 | 0.45995555 | 8.11996278 | 2.33E-15 | 3.36E-15 | 1.80E-12 |
|  | EDC3 | NM_001142443.1 | 4.11343648 | 0.35978475 | 11.4330484 | 0 | 0 | 0 |
|  | EDN1 | NM_001955.2 | 4.64054224 | 0.50068575 | 9.2683729 | 0 | 0 | 0 |
| *(cont. from previous page)*  **CRLM stroma *versus* paired primary CRC stroma** | EGF | NM_001963.4 | 3.18281114 | 0.67993032 | 4.68108429 | 1.13E-05 | 1.21E-05 | 0.00869295 |
|  | EGFL7 | NM_016215.3 | 5.08068787 | 0.42036255 | 12.0864426 | 0 | 0 | 0 |
|  | EGFR | NM_201282.1 | 4.67264199 | 0.39923144 | 11.7040931 | 0 | 0 | 0 |
|  | EGLN2 | NM_053046.3 | 5.24171151 | 0.32507827 | 16.1244599 | 0 | 0 | 0 |
|  | EGLN3 | NM_022073.3 | 4.94798396 | 0.54346946 | 9.10443791 | 0 | 0 | 0 |
|  | EIF2AK3 | NM_004836.5 | 4.44669176 | 0.36120446 | 12.3107333 | 0 | 0 | 0 |
|  | EIF2B4 | NM_172195.3 | 4.64852114 | 0.39418351 | 11.7927845 | 0 | 0 | 0 |
|  | EIF4E2 | NM_004846.3 | 5.44846536 | 0.28037007 | 19.4331207 | 0 | 0 | 0 |
|  | EIF4EBP1 | NM_004095.3 | 4.42325849 | 0.41133368 | 10.7534558 | 0 | 0 | 0 |
|  | ELF3 | NM_001114309.1 | 5.66056028 | 0.47111677 | 12.0151959 | 0 | 0 | 0 |
|  | ELK3 | NM_005230.2 | 5.01841439 | 0.38375109 | 13.0772642 | 0 | 0 | 0 |
|  | EMCN | NM_016242.3 | 4.03735461 | 0.51840056 | 7.78809853 | 3.08E-14 | 4.26E-14 | 2.37E-11 |
|  | EMILIN1 | XM_006711928.1 | 6.45345717 | 0.30070395 | 21.4611655 | 0 | 0 | 0 |
|  | EMP3 | NM_001425.2 | 5.51852209 | 0.37667442 | 14.6506422 | 0 | 0 | 0 |
|  | ENO1 | NM_001428.2 | 6.1054863 | 0.35241259 | 17.3248246 | 0 | 0 | 0 |
|  | ENO2 | NM_001975.2 | 4.81271638 | 0.38904962 | 12.3704435 | 0 | 0 | 0 |
|  | ENO3 | NM_001976.4 | 3.36847883 | 0.50480312 | 6.67285654 | 1.33E-10 | 1.62E-10 | 1.02E-07 |
|  | ENPEP | NM_001977.3 | 3.56636132 | 0.51474071 | 6.92846173 | 1.46E-11 | 1.85E-11 | 1.12E-08 |
|  | ENPP2 | NM_001040092.2 | 4.62475409 | 0.40855985 | 11.319649 | 0 | 0 | 0 |
|  | EP300 | NM_001429.2 | 4.82364766 | 0.3540601 | 13.6238104 | 0 | 0 | 0 |
|  | EPAS1 | NM_001430.3 | 5.64938918 | 0.30351453 | 18.6132412 | 0 | 0 | 0 |
|  | EPCAM | NM_002354.1 | 5.01617287 | 0.50607364 | 9.91194265 | 0 | 0 | 0 |
|  | EPHA1 | NM_005232.3 | 3.31664905 | 0.45687347 | 7.25944774 | 1.34E-12 | 1.76E-12 | 1.03E-09 |
|  | EPHA2 | NM_004431.2 | 4.61998037 | 0.41604122 | 11.1046217 | 0 | 0 | 0 |
|  | EPHB3 | NM_004443.3 | 4.14809702 | 0.63017308 | 6.58247257 | 1.80E-10 | 2.19E-10 | 1.38E-07 |
|  | EPHB4 | NM_004444.4 | 4.95620418 | 0.37083992 | 13.3648075 | 0 | 0 | 0 |
|  | EPN3 | NM_017957.2 | 4.62636996 | 0.477827 | 9.68210253 | 0 | 0 | 0 |
|  | EPS8L1 | NM_017729.3 | 3.45266028 | 0.37933338 | 9.10191527 | 0 | 0 | 0 |
|  | ERBB2 | NM_001005862.1 | 4.70664569 | 0.32490027 | 14.486432 | 0 | 0 | 0 |
|  | ERBB2IP | NM_018695.2 | 5.86458736 | 0.38040363 | 15.4167493 | 0 | 0 | 0 |
|  | ERBB3 | NM_001005915.1 | 4.56536372 | 0.4789405 | 9.53221475 | 0 | 0 | 0 |
|  | ERCC3 | NM_000122.1 | 4.16375286 | 0.35334894 | 11.7836856 | 0 | 0 | 0 |
|  | ERMP1 | NM_024896.2 | 4.29787805 | 0.3866438 | 11.1158594 | 0 | 0 | 0 |
|  | ESRP1 | NM_001034915.2 | 4.25400942 | 0.5003149 | 8.50266381 | 1.11E-16 | 1.64E-16 | 8.55E-14 |
|  | ETV4 | NM_001079675.1 | 4.25009139 | 0.48566417 | 8.75109112 | 0 | 0 | 0 |
|  | EVI2A | NM_014210.3 | 4.03782907 | 0.61067536 | 6.61207143 | 1.25E-10 | 1.54E-10 | 9.65E-08 |
|  | EVPL | NM_001988.2 | 4.43487883 | 0.47536569 | 9.3294045 | 0 | 0 | 0 |
|  | F11R | NM_144503.1 | 5.01452652 | 0.35414862 | 14.1593848 | 0 | 0 | 0 |
|  | F3 | NM_001993.3 | 4.00412462 | 0.41543371 | 9.63842018 | 0 | 0 | 0 |
|  | FAM174B | NM_207446.2 | 4.35026416 | 0.52913143 | 8.22151906 | 7.77E-16 | 1.13E-15 | 5.98E-13 |
|  | FAP | NM_004460.2 | 5.75494557 | 0.46255778 | 12.4415713 | 0 | 0 | 0 |
| *(cont. from previous page)*  **CRLM stroma *versus* paired primary CRC stroma** | FBLN1 | NM_006487.2 | 3.8548755 | 0.51866724 | 7.43227104 | 3.79E-13 | 5.02E-13 | 2.92E-10 |
|  | FBLN5 | NM_006329.3 | 5.49496877 | 0.47438899 | 11.5832552 | 0 | 0 | 0 |
|  | FBN1 | NM_000138.3 | 6.04302989 | 0.43483204 | 13.8973887 | 0 | 0 | 0 |
|  | FBN2 | NM_001999.3 | 3.0288102 | 0.59359196 | 5.1025122 | 1.35E-06 | 1.47E-06 | 0.00104088 |
|  | FBP1 | NM_000507.3 | 5.47682357 | 0.49678551 | 11.0245237 | 0 | 0 | 0 |
|  | FCF1 | NM_015962.4 | 6.53144571 | 0.35487997 | 18.4046618 | 0 | 0 | 0 |
|  | FERMT2 | NM_001135000.1 | 5.27868114 | 0.43005087 | 12.2745507 | 0 | 0 | 0 |
|  | FGF2 | NM_002006.4 | 3.35474573 | 0.57129475 | 5.87218027 | 1.68E-08 | 1.92E-08 | 1.29E-05 |
|  | FGF9 | NM_002010.2 | 2.98596449 | 0.57266157 | 5.21418701 | 7.30E-07 | 7.97E-07 | 0.00056235 |
|  | FGFR1 | NM_015850.2 | 4.46632343 | 0.46643301 | 9.5754874 | 0 | 0 | 0 |
|  | FGFR2 | NM_000141.4 | 4.44745916 | 0.46668322 | 9.52993168 | 0 | 0 | 0 |
|  | FGFR3 | NM_022965.2 | 4.7922545 | 0.57133414 | 8.38783153 | 1.11E-16 | 1.64E-16 | 8.55E-14 |
|  | FGFR4 | NM_002011.3 | 3.98378424 | 0.52941905 | 7.52482228 | 2.05E-13 | 2.76E-13 | 1.57E-10 |
|  | FGL2 | NM_006682.2 | 4.91468408 | 0.49508782 | 9.92689352 | 0 | 0 | 0 |
|  | FHL1 | NM_001449.4 | 5.00154217 | 0.42565255 | 11.7502931 | 0 | 0 | 0 |
|  | FIGF | NM_004469.2 | 3.29820733 | 0.58240689 | 5.66306374 | 5.89E-08 | 6.66E-08 | 4.53E-05 |
|  | FLI1 | NM_001167681.2 | 4.61465072 | 0.50547851 | 9.12927176 | 0 | 0 | 0 |
|  | FLT1 | NM_002019.4 | 5.29356108 | 0.48872336 | 10.8314059 | 0 | 0 | 0 |
|  | FLT4 | NM_002020.1 | 4.09191349 | 0.45905757 | 8.91372619 | 0 | 0 | 0 |
|  | FMOD | NM_002023.3 | 6.63417641 | 0.45328227 | 14.6358612 | 0 | 0 | 0 |
|  | FN1 | NM_212482.1 | 9.25360623 | 0.5814316 | 15.9152105 | 0 | 0 | 0 |
|  | FOXC2 | NM_005251.2 | 4.33204756 | 0.5262918 | 8.23126562 | 1.33E-15 | 1.94E-15 | 1.03E-12 |
|  | FOXO4 | NM_005938.2 | 4.5441062 | 0.30788994 | 14.7588655 | 0 | 0 | 0 |
|  | FRAS1 | NM_001166133.1 | 3.49730869 | 0.40510676 | 8.63305432 | 0 | 0 | 0 |
|  | FREM1 | NM_001177704.1 | 3.6869456 | 0.5697785 | 6.47084016 | 3.96E-10 | 4.76E-10 | 3.05E-07 |
|  | FREM2 | NM_207361.4 | 3.98673522 | 0.60522781 | 6.58716459 | 1.90E-10 | 2.31E-10 | 1.46E-07 |
|  | FST | NM_006350.2 | 3.26195374 | 0.62261398 | 5.23912706 | 6.56E-07 | 7.20E-07 | 0.00050531 |
|  | FSTL1 | NM_007085.4 | 7.22230196 | 0.35703909 | 20.2283227 | 0 | 0 | 0 |
|  | FUT3 | NM_000149.3 | 3.97732004 | 0.61424813 | 6.47510322 | 3.53E-10 | 4.25E-10 | 2.72E-07 |
|  | FXYD6 | NM_001164831.1 | 4.85684353 | 0.39739341 | 12.2217516 | 0 | 0 | 0 |
|  | GALNT7 | NM_017423.2 | 3.36342935 | 0.44903168 | 7.49040548 | 2.32E-13 | 3.11E-13 | 1.78E-10 |
|  | GATA4 | NM_002052.3 | 4.4517934 | 0.6264598 | 7.10627152 | 5.78E-12 | 7.40E-12 | 4.45E-09 |
|  | GDF15 | NM_004864.2 | 4.56543665 | 0.60603357 | 7.53330648 | 1.72E-13 | 2.33E-13 | 1.33E-10 |
|  | GDF5 | NM_000557.2 | 4.05321326 | 0.68533693 | 5.91419067 | 1.26E-08 | 1.45E-08 | 9.72E-06 |
|  | GDF6 | NM_001001557.2 | 3.7772759 | 0.62749127 | 6.01964693 | 6.39E-09 | 7.41E-09 | 4.92E-06 |
|  | GIMAP4 | NM_018326.2 | 5.65994171 | 0.44152355 | 12.8191163 | 0 | 0 | 0 |
|  | GIMAP6 | NR_024115.1 | 3.78090568 | 0.49199685 | 7.68481683 | 5.94E-14 | 8.17E-14 | 4.57E-11 |
|  | GJA5 | NM_005266.5 | 4.35576189 | 0.46123922 | 9.44360689 | 0 | 0 | 0 |
|  | GPATCH3 | NM_022078.2 | 3.92265122 | 0.39795649 | 9.85698528 | 0 | 0 | 0 |
|  | GPI | NM_000175.2 | 5.91517959 | 0.36276162 | 16.3059688 | 0 | 0 | 0 |
|  | GPR124 | NM_032777.9 | 5.45964323 | 0.44001511 | 12.407854 | 0 | 0 | 0 |
| *(cont. from previous page)*  **CRLM stroma *versus* paired primary CRC stroma** | GPR56 | NM_005682.4 | 5.38595178 | 0.41208382 | 13.0700393 | 0 | 0 | 0 |
|  | GPX1 | NM_000581.2 | 6.11939918 | 0.32462342 | 18.8507631 | 0 | 0 | 0 |
|  | GREM1 | NM_013372.5 | 6.01650586 | 0.5181993 | 11.6104092 | 0 | 0 | 0 |
|  | GRHL2 | NM_024915.3 | 3.89439463 | 0.5079672 | 7.66662619 | 5.86E-14 | 8.07E-14 | 4.51E-11 |
|  | GSN | NM_000177.4 | 6.60097702 | 0.38485641 | 17.151792 | 0 | 0 | 0 |
|  | GTF2I | NM_033001.2 | 5.85015096 | 0.29572966 | 19.7820907 | 0 | 0 | 0 |
|  | GZMK | NM_002104.2 | 4.43681759 | 0.67602038 | 6.56314181 | 1.91E-10 | 2.32E-10 | 1.47E-07 |
|  | HAS1 | NM_001523.2 | 4.50462795 | 0.59915537 | 7.51829688 | 3.07E-13 | 4.10E-13 | 2.36E-10 |
|  | HDAC3 | NM_003883.2 | 4.17535019 | 0.41611566 | 10.0341097 | 0 | 0 | 0 |
|  | HDAC5 | NM_005474.4 | 5.18501197 | 0.35487241 | 14.6109187 | 0 | 0 | 0 |
|  | HDHD3 | NM_031219.2 | 4.20737108 | 0.39065792 | 10.7699624 | 0 | 0 | 0 |
|  | HEG1 | NM_020733.1 | 5.96055724 | 0.40946369 | 14.5569861 | 0 | 0 | 0 |
|  | HGF | NM_000601.4 | 5.12551003 | 0.51831014 | 9.88888636 | 0 | 0 | 0 |
|  | HIF1A | NM_001530.2 | 6.93913678 | 0.35192696 | 19.7175482 | 0 | 0 | 0 |
|  | HIPK1 | NM_152696.3 | 4.80706904 | 0.31050301 | 15.4815537 | 0 | 0 | 0 |
|  | HIPK2 | NM_022740.4 | 4.91083343 | 0.3696144 | 13.2863693 | 0 | 0 | 0 |
|  | HK2 | NM_000189.4 | 5.33065241 | 0.40226637 | 13.2515487 | 0 | 0 | 0 |
|  | HK3 | NM_002115.1 | 4.03388235 | 0.53832364 | 7.49341488 | 2.81E-13 | 3.75E-13 | 2.16E-10 |
|  | HKDC1 | NM_025130.3 | 4.48430928 | 0.44035994 | 10.1832816 | 0 | 0 | 0 |
|  | HLA-DPB1 | NM_002121.4 | 7.14579692 | 0.51387117 | 13.9058141 | 0 | 0 | 0 |
|  | HMOX1 | NM_002133.2 | 5.36224549 | 0.39380634 | 13.616453 | 0 | 0 | 0 |
|  | HOXA5 | NM_019102.2 | 3.95609737 | 0.45022823 | 8.78687107 | 0 | 0 | 0 |
|  | HOXA7 | NM_006896.3 | 4.19758616 | 0.43996128 | 9.5408081 | 0 | 0 | 0 |
|  | HOXB13 | NM_006361.5 | 3.40752596 | 0.59275992 | 5.74857683 | 3.66E-08 | 4.16E-08 | 2.82E-05 |
|  | HOXB3 | NM_002146.4 | 5.53600421 | 0.35837566 | 15.4474896 | 0 | 0 | 0 |
|  | HPSE | NM_006665.3 | 4.34440144 | 0.43182285 | 10.0606103 | 0 | 0 | 0 |
|  | HRAS | NM_005343.2 | 3.49325058 | 0.44546922 | 7.8417327 | 1.55E-14 | 2.18E-14 | 1.20E-11 |
|  | HSD17B12 | NM_016142.2 | 5.51443404 | 0.42171661 | 13.0761604 | 0 | 0 | 0 |
|  | HSP90B1 | NM_003299.1 | 6.77498907 | 0.35566612 | 19.0487333 | 0 | 0 | 0 |
|  | HSPB1 | NM_001540.3 | 5.45064265 | 0.38187733 | 14.2732816 | 0 | 0 | 0 |
|  | HSPG2 | NM_005529.5 | 7.09372676 | 0.28309919 | 25.0573898 | 0 | 0 | 0 |
|  | HUNK | NM_014586.1 | 3.33528477 | 0.61717418 | 5.40412237 | 2.56E-07 | 2.83E-07 | 0.00019684 |
|  | IBSP | NM_004967.3 | 3.21530625 | 0.50664016 | 6.34633123 | 1.71E-09 | 2.03E-09 | 1.31E-06 |
|  | ICAM1 | NM_000201.2 | 5.64964519 | 0.40735855 | 13.8689741 | 0 | 0 | 0 |
|  | ID1 | NM_002165.2 | 5.20750157 | 0.55682918 | 9.35206308 | 0 | 0 | 0 |
|  | ID2 | NM_002166.4 | 4.77982411 | 0.40484642 | 11.8065119 | 0 | 0 | 0 |
|  | ID4 | NM_001546.2 | 6.58068348 | 0.4506708 | 14.6019743 | 0 | 0 | 0 |
|  | IFNG | NM_000619.2 | 4.13407431 | 0.57875909 | 7.14299672 | 3.04E-12 | 3.95E-12 | 2.34E-09 |
|  | IGF1 | NM_000618.3 | 3.72326888 | 0.5369785 | 6.93373925 | 1.48E-11 | 1.88E-11 | 1.14E-08 |
|  | IGFBP4 | NM_001552.2 | 7.35812548 | 0.3643541 | 20.1949846 | 0 | 0 | 0 |
|  | IGFBP7 | NM_001553.1 | 9.24714068 | 0.41934892 | 22.0511851 | 0 | 0 | 0 |
| *(cont. from previous page)*  **CRLM stroma *versus* paired primary CRC stroma** | IL10RA | NM_001558.2 | 5.69241486 | 0.42461067 | 13.4061983 | 0 | 0 | 0 |
|  | IL11 | NM_000641.2 | 3.73760788 | 0.6756689 | 5.53171518 | 1.24E-07 | 1.38E-07 | 9.54E-05 |
|  | IL13RA2 | NM_000640.2 | 2.77105369 | 0.57416492 | 4.82623305 | 5.24E-06 | 5.67E-06 | 0.00403163 |
|  | IL15 | NM_172174.1 | 4.22509111 | 0.53875636 | 7.84230393 | 1.45E-14 | 2.04E-14 | 1.12E-11 |
|  | IL18 | NM_001562.2 | 4.65707801 | 0.4217663 | 11.0418447 | 0 | 0 | 0 |
|  | IL1A | NM_000575.3 | 3.71563856 | 0.56025556 | 6.63204233 | 1.18E-10 | 1.44E-10 | 9.05E-08 |
|  | IL1B | NM_000576.2 | 3.79394511 | 0.58848711 | 6.44694683 | 4.34E-10 | 5.21E-10 | 3.34E-07 |
|  | IL1RL1 | NM_016232.4 | 3.29735265 | 0.54484618 | 6.05189646 | 4.96E-09 | 5.81E-09 | 3.82E-06 |
|  | IL1RN | NM_000577.3 | 4.21185509 | 0.45329067 | 9.29173119 | 0 | 0 | 0 |
|  | IL6 | NM_000600.1 | 4.40082913 | 0.70817827 | 6.21429561 | 2.96E-09 | 3.48E-09 | 2.28E-06 |
|  | ILK | NM_004517.2 | 5.12011862 | 0.32726846 | 15.6450107 | 0 | 0 | 0 |
|  | INHBA | NM_002192.2 | 6.83688012 | 0.37738699 | 18.1163643 | 0 | 0 | 0 |
|  | INHBE | NM_031479.3 | 4.04531441 | 0.64539015 | 6.26801386 | 1.81E-09 | 2.14E-09 | 1.39E-06 |
|  | IRF6 | NM_006147.2 | 4.76407802 | 0.46282171 | 10.2935492 | 0 | 0 | 0 |
|  | ISL1 | NM_002202.2 | 3.56123501 | 0.5577086 | 6.38547621 | 7.17E-10 | 8.57E-10 | 5.52E-07 |
|  | ISLR | NM_005545.3 | 6.3533773 | 0.43125013 | 14.7324646 | 0 | 0 | 0 |
|  | ITGA11 | NM_012211.3 | 5.92667106 | 0.51393853 | 11.5318675 | 0 | 0 | 0 |
|  | ITGA2 | NM_002203.2 | 5.28918204 | 0.4335355 | 12.2001129 | 0 | 0 | 0 |
|  | ITGA3 | NM_002204.2 | 5.08384549 | 0.28262837 | 17.9877398 | 0 | 0 | 0 |
|  | ITGA5 | NM_002205.2 | 5.40755191 | 0.55947529 | 9.66539907 | 0 | 0 | 0 |
|  | ITGA6 | NM_000210.1 | 4.78117213 | 0.4627413 | 10.3322789 | 0 | 0 | 0 |
|  | ITGA7 | NM_002206.1 | 3.41720361 | 0.4869187 | 7.0180168 | 8.32E-12 | 1.06E-11 | 6.40E-09 |
|  | ITGA8 | NM_003638.1 | 3.45558605 | 0.60056322 | 5.75390886 | 3.25E-08 | 3.70E-08 | 2.50E-05 |
|  | ITGA9 | NM_002207.2 | 5.14807227 | 0.51756654 | 9.9466869 | 0 | 0 | 0 |
|  | ITGAM | NM_000632.3 | 4.75543386 | 0.56121922 | 8.47339813 | 0 | 0 | 0 |
|  | ITGB1 | NM_033666.2 | 7.48802427 | 0.31911651 | 23.4648605 | 0 | 0 | 0 |
|  | ITGB1BP1 | NM_004763.3 | 3.60550098 | 0.48062527 | 7.5016884 | 2.26E-13 | 3.03E-13 | 1.74E-10 |
|  | ITGB2 | NM_001127491.1 | 5.72984403 | 0.45968954 | 12.4645952 | 0 | 0 | 0 |
|  | ITGB3 | NM_000212.2 | 3.25303701 | 0.43576321 | 7.46514839 | 3.08E-13 | 4.10E-13 | 2.37E-10 |
|  | ITGB4 | NM_001005731.1 | 5.31901561 | 0.42316955 | 12.5694668 | 0 | 0 | 0 |
|  | ITGB7 | NM_000889.1 | 3.44129606 | 0.7097412 | 4.84866326 | 4.79E-06 | 5.20E-06 | 0.00369101 |
|  | ITGB8 | NM_002214.2 | 4.34528905 | 0.48535761 | 8.95275767 | 0 | 0 | 0 |
|  | ITM2A | NM_004867.4 | 3.62732618 | 0.57409075 | 6.31838469 | 1.06E-09 | 1.27E-09 | 8.19E-07 |
|  | JAG1 | NM_000214.2 | 5.8889155 | 0.38961672 | 15.1146376 | 0 | 0 | 0 |
|  | JAM2 | NM_001270407.1 | 3.77362788 | 0.49457607 | 7.63002517 | 7.83E-14 | 1.07E-13 | 6.03E-11 |
|  | JAM3 | NM_032801.3 | 5.33225403 | 0.43131705 | 12.3627248 | 0 | 0 | 0 |
|  | JUN | NM_002228.3 | 5.50474345 | 0.46101835 | 11.9403999 | 0 | 0 | 0 |
|  | KCNJ8 | NM_004982.2 | 4.50515335 | 0.5165553 | 8.72153162 | 0 | 0 | 0 |
|  | KDR | NM_002253.2 | 4.16793609 | 0.51549863 | 8.08525153 | 3.22E-15 | 4.63E-15 | 2.48E-12 |
|  | KIAA1462 | NM_020848.2 | 4.0098647 | 0.56064533 | 7.15223065 | 2.92E-12 | 3.79E-12 | 2.25E-09 |
|  | KISS1 | NM_002256.3 | 3.42092979 | 0.55172921 | 6.20037825 | 2.43E-09 | 2.86E-09 | 1.87E-06 |
| *(cont. from previous page)*  **CRLM stroma *versus* paired primary CRC stroma** | KLK3 | NM_001030049.1 | 3.3990628 | 0.68466071 | 4.96459451 | 3.01E-06 | 3.27E-06 | 0.002317 |
|  | KRAS | NM_004985.3 | 5.20240198 | 0.43348672 | 12.0012949 | 0 | 0 | 0 |
|  | KRIT1 | NM_004912.3 | 3.66954748 | 0.35647595 | 10.2939552 | 0 | 0 | 0 |
|  | KRT1 | NM_006121.2 | 2.99821497 | 0.68048428 | 4.40600181 | 4.38E-05 | 4.64E-05 | 0.03370137 |
|  | KRT19 | NM_002276.4 | 5.46230527 | 0.46522262 | 11.7412719 | 0 | 0 | 0 |
|  | KRT7 | NM_005556.3 | 5.43175661 | 0.46530146 | 11.6736291 | 0 | 0 | 0 |
|  | LAD1 | NM_005558.3 | 4.80138345 | 0.44147276 | 10.8758317 | 0 | 0 | 0 |
|  | LAMA3 | NM_000227.3 | 3.60858189 | 0.45375513 | 7.95270772 | 7.99E-15 | 1.13E-14 | 6.16E-12 |
|  | LAMA4 | NM_001105209.1 | 5.79112482 | 0.31584221 | 18.3355 | 0 | 0 | 0 |
|  | LAMA5 | NM_005560.3 | 5.45970936 | 0.40835131 | 13.3701281 | 0 | 0 | 0 |
|  | LAMB3 | NM_000228.2 | 4.61535324 | 0.45292751 | 10.1900484 | 0 | 0 | 0 |
|  | LAMC1 | NM_002293.3 | 5.75758502 | 0.2604905 | 22.1028599 | 0 | 0 | 0 |
|  | LAMC2 | NM_005562.2 | 5.41803934 | 0.42714554 | 12.6842935 | 0 | 0 | 0 |
|  | LDHA | NM_001165414.1 | 7.12216252 | 0.41326042 | 17.2340786 | 0 | 0 | 0 |
|  | LGALS1 | NM_002305.3 | 7.46709284 | 0.49048156 | 15.2240032 | 0 | 0 | 0 |
|  | LHFP | NM_005780.2 | 5.413822 | 0.44118099 | 12.2712042 | 0 | 0 | 0 |
|  | LIFR | NM_002310.3 | 3.71322775 | 0.53234439 | 6.97523601 | 1.20E-11 | 1.53E-11 | 9.27E-09 |
|  | LLGL2 | NM_001015002.1 | 4.70196672 | 0.45327689 | 10.3732769 | 0 | 0 | 0 |
|  | LOX | NM_002317.4 | 5.23747046 | 0.57205708 | 9.15550331 | 0 | 0 | 0 |
|  | LOXL2 | NM_002318.2 | 4.66784183 | 0.5211946 | 8.95604407 | 0 | 0 | 0 |
|  | LRG1 | NM_052972.2 | 5.05436828 | 0.56612813 | 8.92795816 | 0 | 0 | 0 |
|  | LTBP4 | NM_003573.2 | 5.42865995 | 0.37417038 | 14.5085242 | 0 | 0 | 0 |
|  | LUM | NM_002345.3 | 7.8872821 | 0.41894236 | 18.8266522 | 0 | 0 | 0 |
|  | LY96 | NM_015364.2 | 4.75587863 | 0.61213817 | 7.76928943 | 2.92E-14 | 4.06E-14 | 2.25E-11 |
|  | MAF | NM_005360.4 | 5.17778477 | 0.38469274 | 13.4595334 | 0 | 0 | 0 |
|  | MAP2K1 | NM_002755.2 | 4.81179239 | 0.34391824 | 13.9910939 | 0 | 0 | 0 |
|  | MAP2K2 | NM_030662.3 | 5.90575323 | 0.30968671 | 19.0700896 | 0 | 0 | 0 |
|  | MAP2K4 | NM_003010.2 | 4.51237553 | 0.3810897 | 11.8407178 | 0 | 0 | 0 |
|  | MAP3K7 | NM_145333.1 | 4.90906167 | 0.37159449 | 13.2108031 | 0 | 0 | 0 |
|  | MAPK1 | NM_138957.2 | 4.59936375 | 0.33734287 | 13.6340922 | 0 | 0 | 0 |
|  | MAPK3 | NM_001040056.1 | 5.08867483 | 0.35258841 | 14.4323371 | 0 | 0 | 0 |
|  | MAPKAPK3 | NM_004635.3 | 3.71830973 | 0.37349438 | 9.95546379 | 0 | 0 | 0 |
|  | MCAM | NM_006500.2 | 4.72016288 | 0.42819303 | 11.0234462 | 0 | 0 | 0 |
|  | MED1 | NM_004774.3 | 4.31181907 | 0.39149643 | 11.0136868 | 0 | 0 | 0 |
|  | MED23 | NM_004830.2 | 3.8136072 | 0.37963777 | 10.0453842 | 0 | 0 | 0 |
|  | Niban-like protein 1 | NR_002766.2 | 5.47328296 | 0.34405073 | 15.9083602 | 0 | 0 | 0 |
|  | MEOX2 | NM_005924.4 | 2.96695241 | 0.66478401 | 4.46303219 | 3.25E-05 | 3.46E-05 | 0.02503469 |
|  | L-methionine | NM_001127500.1 | 3.8106507 | 0.47472813 | 8.02701693 | 4.55E-15 | 6.49E-15 | 3.50E-12 |
|  | MFAP4 | NM_002404.1 | 5.38168019 | 0.5321391 | 10.1132959 | 0 | 0 | 0 |
|  | MGAT5 | NM_002410.4 | 4.63880338 | 0.40948887 | 11.3282772 | 0 | 0 | 0 |
|  | MGP | NM_000900.2 | 7.76152363 | 0.54501085 | 14.2410442 | 0 | 0 | 0 |
| *(cont. from previous page)*  **CRLM stroma *versus* paired primary CRC stroma** | MMP12 | NM_002426.3 | 5.22255652 | 0.87830262 | 5.94619258 | 1.00E-08 | 1.15E-08 | 7.70E-06 |
|  | MMP13 | NM_002427.2 | 3.3112789 | 0.59378095 | 5.57660009 | 9.26E-08 | 1.04E-07 | 7.13E-05 |
|  | MMP14 | NM_004995.2 | 6.11578245 | 0.37270868 | 16.4090152 | 0 | 0 | 0 |
|  | MMP2 | NM_004530.2 | 5.08450541 | 0.46912217 | 10.8383397 | 0 | 0 | 0 |
|  | MMP9 | NM_004994.2 | 5.22909555 | 0.56536192 | 9.24911163 | 0 | 0 | 0 |
|  | MMRN2 | NM_024756.2 | 4.72246833 | 0.46042103 | 10.2568476 | 0 | 0 | 0 |
|  | MPDZ | NM_003829.4 | 4.76295252 | 0.48197801 | 9.88209501 | 0 | 0 | 0 |
|  | MRC1 | NM_002438.2 | 5.23157374 | 0.58519633 | 8.93986089 | 0 | 0 | 0 |
|  | MRPS5 | NM_031902.3 | 4.66564655 | 0.41660069 | 11.199325 | 0 | 0 | 0 |
|  | MS4A4A | NM_024021.2 | 5.67926441 | 0.45115858 | 12.5881777 | 0 | 0 | 0 |
|  | MS4A6A | NM_152852.2 | 4.350096 | 0.55971483 | 7.77198639 | 2.55E-14 | 3.56E-14 | 1.97E-11 |
|  | MT3 | NM_005954.2 | 3.75455623 | 0.55366702 | 6.78125315 | 4.17E-11 | 5.22E-11 | 3.21E-08 |
|  | MTA1 | NM_004689.2 | 5.03039677 | 0.33641387 | 14.9530004 | 0 | 0 | 0 |
|  | MTBP | NM_022045.4 | 5.20397095 | 0.3359054 | 15.4923705 | 0 | 0 | 0 |
|  | MTDH | NM_178812.3 | 5.05001134 | 0.35338272 | 14.2904875 | 0 | 0 | 0 |
|  | MTMR14 | NM_022485.3 | 4.76879775 | 0.35349905 | 13.4902705 | 0 | 0 | 0 |
|  | Serine/threonine-protein kinase mTOR | NM_004958.2 | 4.64676648 | 0.35472497 | 13.0996316 | 0 | 0 | 0 |
|  | MYC | NM_002467.3 | 5.55862188 | 0.49064234 | 11.3292747 | 0 | 0 | 0 |
|  | MYCL1 | NM_001033081.2 | 3.90228499 | 0.44727499 | 8.72457687 | 0 | 0 | 0 |
|  | MYH11 | NM_001040113.1 | 4.89528369 | 0.62245623 | 7.86446258 | 1.41E-14 | 1.98E-14 | 1.09E-11 |
|  | MYLK | NM_053032.2 | 6.3732094 | 0.39479906 | 16.1429192 | 0 | 0 | 0 |
|  | MYO1D | NM_015194.1 | 5.29667897 | 0.43100613 | 12.2891037 | 0 | 0 | 0 |
|  | MYO5C | NM_018728.2 | 4.48016797 | 0.42957761 | 10.42924 | 0 | 0 | 0 |
|  | NAA15 | NM_057175.3 | 4.60285294 | 0.35738216 | 12.8793584 | 0 | 0 | 0 |
|  | NAP1L3 | NM_004538.4 | 3.26374106 | 0.58675584 | 5.56234951 | 1.03E-07 | 1.15E-07 | 7.90E-05 |
|  | NCAM1 | NM_000615.5 | 3.91214056 | 0.58942527 | 6.63721217 | 1.13E-10 | 1.39E-10 | 8.72E-08 |
|  | NCL | NM_005381.2 | 5.23164148 | 0.38932857 | 13.4375997 | 0 | 0 | 0 |
|  | a nucleoside diphosphate | NM_000266.2 | 2.91979177 | 0.63411124 | 4.60454191 | 1.55E-05 | 1.66E-05 | 0.01194671 |
|  | NDRG1 | NM_006096.2 | 5.50770975 | 0.51196796 | 10.7579188 | 0 | 0 | 0 |
|  | NF1 | NM_000267.2 | 4.64639831 | 0.32254925 | 14.4052368 | 0 | 0 | 0 |
|  | NF2 | NM_181825.2 | 4.09230621 | 0.37083355 | 11.0354261 | 0 | 0 | 0 |
|  | NFAT5 | NM_173214.1 | 4.14453741 | 0.39595295 | 10.4672472 | 0 | 0 | 0 |
|  | NFATC2 | NM_012340.3 | 5.29577692 | 0.44998032 | 11.7689079 | 0 | 0 | 0 |
|  | NFKB1 | NM_003998.2 | 4.10316866 | 0.42812169 | 9.58411773 | 0 | 0 | 0 |
|  | NID2 | NM_007361.3 | 4.48113755 | 0.42748324 | 10.482604 | 0 | 0 | 0 |
|  | NME1 | NM_000269.2 | 4.37263235 | 0.43173354 | 10.1280813 | 0 | 0 | 0 |
|  | NME4 | NM_005009.2 | 4.60739389 | 0.38420243 | 11.9920997 | 0 | 0 | 0 |
|  | NODAL | NM_018055.3 | 4.20956903 | 0.6028567 | 6.98270264 | 9.14E-12 | 1.17E-11 | 7.04E-09 |
|  | NOL7 | NM_016167.3 | 4.46347354 | 0.40941135 | 10.9021734 | 0 | 0 | 0 |
|  | NOS3 | NM_000603.4 | 4.03298523 | 0.37512596 | 10.751016 | 0 | 0 | 0 |
| *(cont. from previous page)*  **CRLM stroma *versus* paired primary CRC stroma** | NOTCH1 | NM_017617.3 | 5.3377616 | 0.35860288 | 14.884882 | 0 | 0 | 0 |
|  | NOX5 | NM_024505.2 | 3.28179556 | 0.58443343 | 5.61534538 | 7.33E-08 | 8.24E-08 | 5.64E-05 |
|  | NPR1 | NM_000906.2 | 4.07465487 | 0.62815611 | 6.48669145 | 4.04E-10 | 4.86E-10 | 3.11E-07 |
|  | NR3C1 | NM_001018077.1 | 5.75835961 | 0.43904558 | 13.1156305 | 0 | 0 | 0 |
|  | NR4A1 | NM_173157.1 | 6.61285389 | 0.65457044 | 10.1025855 | 0 | 0 | 0 |
|  | NR4A3 | NM_173198.1 | 5.15132644 | 0.49120428 | 10.4871367 | 0 | 0 | 0 |
|  | NRCAM | NM_005010.4 | 3.4636306 | 0.65837389 | 5.26088693 | 6.87E-07 | 7.52E-07 | 0.00052933 |
|  | NRP1 | NM_003873.5 | 7.09445143 | 0.3820673 | 18.5685912 | 0 | 0 | 0 |
|  | NRP2 | NM_003872.2 | 5.52187251 | 0.50820524 | 10.8654379 | 0 | 0 | 0 |
|  | NRXN1 | NM_138735.2 | 3.42651241 | 0.63348432 | 5.40899326 | 2.49E-07 | 2.76E-07 | 0.00019196 |
|  | NRXN3 | NM_001105250.1 | 3.9856986 | 0.58492153 | 6.81407401 | 3.31E-11 | 4.16E-11 | 2.55E-08 |
|  | NTRK1 | NM_001012331.1 | 4.1308514 | 0.5440229 | 7.59315727 | 1.33E-13 | 1.80E-13 | 1.02E-10 |
|  | NUBP1 | NM_001278506.1 | 2.48477894 | 0.39121722 | 6.35140479 | 9.29E-10 | 1.11E-09 | 7.15E-07 |
|  | OAS1 | NM_001032409.1 | 4.98385875 | 0.4167159 | 11.9598479 | 0 | 0 | 0 |
|  | OCLN | NM_002538.3 | 3.11915082 | 0.55279223 | 5.64253742 | 6.46E-08 | 7.30E-08 | 4.98E-05 |
|  | OGN | NM_014057.3 | 4.20341499 | 0.61608762 | 6.82275512 | 3.65E-11 | 4.58E-11 | 2.81E-08 |
|  | OLFML2B | NM_015441.1 | 4.62262497 | 0.45818365 | 10.0890222 | 0 | 0 | 0 |
|  | OVOL2 | NM_021220.2 | 4.36832114 | 0.56267946 | 7.76342748 | 2.53E-14 | 3.53E-14 | 1.95E-11 |
|  | P3H1 | NM_001146289.1 | 4.69208175 | 0.36295622 | 12.9274042 | 0 | 0 | 0 |
|  | PCOLCE | NM_002593.3 | 5.65023033 | 0.39948735 | 14.1437027 | 0 | 0 | 0 |
|  | PDCD10 | NM_145859.1 | 5.19439933 | 0.32512325 | 15.9767083 | 0 | 0 | 0 |
|  | PDCL3 | NM_024065.4 | 4.88277146 | 0.31439184 | 15.5308466 | 0 | 0 | 0 |
|  | PDGFA | NM_002607.5 | 4.9909057 | 0.37456693 | 13.3244696 | 0 | 0 | 0 |
|  | PDGFC | NM_016205.2 | 4.84655068 | 0.49401131 | 9.81060675 | 0 | 0 | 0 |
|  | PDGFRB | NM_002609.3 | 6.06899973 | 0.42072838 | 14.424983 | 0 | 0 | 0 |
|  | PDK1 | NM_002610.3 | 4.34373298 | 0.56931571 | 7.62974379 | 6.92E-14 | 9.49E-14 | 5.33E-11 |
|  | PDPN | NM_006474.4 | 4.81790426 | 0.56591147 | 8.51353004 | 0 | 0 | 0 |
|  | PEBP4 | NM_144962.2 | 4.73413571 | 0.30633167 | 15.4542811 | 0 | 0 | 0 |
|  | PECAM1 | NM_000442.3 | 6.12745664 | 0.50188827 | 12.2088062 | 0 | 0 | 0 |
|  | PFKFB1 | NM_002625.2 | 3.60314944 | 0.59704117 | 6.03501005 | 5.96E-09 | 6.93E-09 | 4.59E-06 |
|  | PFKFB4 | NM_004567.2 | 2.92106994 | 0.61669293 | 4.73666845 | 8.95E-06 | 9.63E-06 | 0.00689187 |
|  | PGK1 | NM_000291.2 | 7.26108715 | 0.32963226 | 22.0278412 | 0 | 0 | 0 |
|  | PIK3CA | NM_006218.2 | 4.19470442 | 0.34764613 | 12.0660178 | 0 | 0 | 0 |
|  | PIK3CD | NM_005026.3 | 4.7286134 | 0.45077369 | 10.4899943 | 0 | 0 | 0 |
|  | PIK3CG | NM_002649.2 | 5.19702391 | 0.54236686 | 9.58211918 | 0 | 0 | 0 |
|  | PIK3R1 | NM_181504.2 | 4.8272616 | 0.37995981 | 12.7046638 | 0 | 0 | 0 |
|  | PIK3R2 | NM_005027.2 | 4.69143213 | 0.28819152 | 16.2788692 | 0 | 0 | 0 |
|  | PIK3R5 | NM_001142633.1 | 4.8480785 | 0.52482148 | 9.2375764 | 0 | 0 | 0 |
|  | PIK3R6 | NM_001010855.3 | 3.77474919 | 0.60554693 | 6.23361956 | 1.71E-09 | 2.03E-09 | 1.32E-06 |
|  | PITX2 | NM_000325.5 | 4.31591838 | 0.56181662 | 7.68207669 | 8.39E-14 | 1.15E-13 | 6.46E-11 |
|  | PKM2 | NM_182471.1 | 5.9699919 | 0.37497306 | 15.9211223 | 0 | 0 | 0 |
| *(cont. from previous page)*  **CRLM stroma *versus* paired primary CRC stroma** | PKN1 | NM_213560.1 | 5.25706395 | 0.4140548 | 12.6965417 | 0 | 0 | 0 |
|  | PKNOX1 | NM_004571.3 | 4.38635787 | 0.43714973 | 10.0339942 | 0 | 0 | 0 |
|  | PLA2G10 | NM_003561.1 | 2.49024595 | 0.55344409 | 4.49954386 | 2.66E-05 | 2.84E-05 | 0.0204984 |
|  | PLA2G2A | NM_000300.2 | 4.01321004 | 0.70618676 | 5.68293015 | 5.34E-08 | 6.05E-08 | 4.12E-05 |
|  | PLA2G2D | NM_001271814.1 | 3.87855929 | 0.62294658 | 6.22615074 | 1.89E-09 | 2.24E-09 | 1.46E-06 |
|  | PLA2G3 | NM_015715.3 | 3.18347438 | 0.65847423 | 4.83462261 | 5.24E-06 | 5.67E-06 | 0.00403537 |
|  | PLAU | NM_002658.2 | 5.2895582 | 0.41603459 | 12.7142269 | 0 | 0 | 0 |
|  | PLAUR | NM_001005376.1 | 5.1846465 | 0.4148218 | 12.498491 | 0 | 0 | 0 |
|  | PLCG1 | NM_002660.2 | 5.08474605 | 0.43863056 | 11.5923206 | 0 | 0 | 0 |
|  | PLCG2 | NM_002661.2 | 4.65763914 | 0.66927812 | 6.95919827 | 1.22E-11 | 1.55E-11 | 9.41E-09 |
|  | PLEKHO1 | NM_016274.4 | 6.20363228 | 0.40653761 | 15.2596763 | 0 | 0 | 0 |
|  | PLS1 | NM_002670.2 | 3.81796182 | 0.51988088 | 7.34391657 | 6.89E-13 | 9.12E-13 | 5.31E-10 |
|  | PLXDC1 | NM_020405.4 | 4.63981355 | 0.45874326 | 10.1141837 | 0 | 0 | 0 |
|  | PLXNC1 | NM_005761.2 | 5.19639428 | 0.38096568 | 13.6400587 | 0 | 0 | 0 |
|  | PLXND1 | NM_015103.2 | 5.30178679 | 0.43961066 | 12.0601872 | 0 | 0 | 0 |
|  | PMP22 | NM_000304.2 | 5.92005037 | 0.44974549 | 13.1631123 | 0 | 0 | 0 |
|  | PNPLA6 | NM_006702.3 | 4.68660916 | 0.34492661 | 13.5872649 | 0 | 0 | 0 |
|  | POSTN | NM_001135935.1 | 8.08925376 | 0.51070153 | 15.8394938 | 0 | 0 | 0 |
|  | PPFIBP2 | NM_003621.2 | 3.95145087 | 0.46355249 | 8.52427921 | 0 | 0 | 0 |
|  | PPP1R16B | NM_015568.2 | 4.58679462 | 0.63264321 | 7.25020764 | 1.70E-12 | 2.23E-12 | 1.31E-09 |
|  | PPP2CB | NM_001009552.1 | 5.88293134 | 0.39054171 | 15.063516 | 0 | 0 | 0 |
|  | PPP2R1A | NM_014225.3 | 6.06703684 | 0.32673945 | 18.5684244 | 0 | 0 | 0 |
|  | PRELP | NM_002725.3 | 5.75397291 | 0.55083 | 10.4460051 | 0 | 0 | 0 |
|  | PRF1 | NM_005041.3 | 4.5355833 | 0.44115668 | 10.2811167 | 0 | 0 | 0 |
|  | PRKCB | NM_212535.1 | 3.70642185 | 0.51852651 | 7.14798911 | 3.44E-12 | 4.46E-12 | 2.65E-09 |
|  | PRKCZ | NM_002744.4 | 3.33668775 | 0.43856304 | 7.60822839 | 9.38E-14 | 1.28E-13 | 7.22E-11 |
|  | PROK2 | NM_021935.3 | 3.87560495 | 0.6436308 | 6.02147212 | 6.43E-09 | 7.44E-09 | 4.95E-06 |
|  | PROM1 | NM_006017.1 | 4.32424106 | 0.65778018 | 6.57399115 | 1.69E-10 | 2.06E-10 | 1.30E-07 |
|  | PRPF38A | NM_032864.3 | 4.95042259 | 0.4112071 | 12.0387577 | 0 | 0 | 0 |
|  | PRSS22 | NM_022119.3 | 4.3340582 | 0.44833129 | 9.66708831 | 0 | 0 | 0 |
|  | PRSS8 | NM_002773.3 | 4.42017315 | 0.47175513 | 9.36963449 | 0 | 0 | 0 |
|  | PTEN | NM_000314.4 | 6.20657927 | 0.3549963 | 17.4835044 | 0 | 0 | 0 |
|  | PTGDS | NM_000954.5 | 5.70679633 | 0.58216293 | 9.80274779 | 0 | 0 | 0 |
|  | PTGIS | NM_000961.3 | 6.5104084 | 0.52051228 | 12.5076943 | 0 | 0 | 0 |
|  | PTGS2 | NM_000963.1 | 4.7241771 | 0.49767 | 9.4925896 | 0 | 0 | 0 |
|  | PTK2 | NM_153831.2 | 4.39998999 | 0.37335478 | 11.7850105 | 0 | 0 | 0 |
|  | PTK2B | NM_004103.3 | 4.88816448 | 0.44559068 | 10.9700779 | 0 | 0 | 0 |
|  | PTK6 | NM_005975.2 | 4.09649161 | 0.4801356 | 8.53194722 | 1.11E-16 | 1.64E-16 | 8.55E-14 |
|  | PTPRB | NM_002837.3 | 4.39556818 | 0.44213028 | 9.94179395 | 0 | 0 | 0 |
|  | PTPRC | NM_080923.2 | 5.12876635 | 0.51804551 | 9.90022357 | 0 | 0 | 0 |
|  | PTPRM | NM_002845.3 | 4.78072211 | 0.46684872 | 10.2404097 | 0 | 0 | 0 |
| *(cont. from previous page)*  **CRLM stroma *versus* paired primary CRC stroma** | PTRF | NM_012232.5 | 5.20637767 | 0.38480504 | 13.5299102 | 0 | 0 | 0 |
|  | PTTG1 | NM_004219.2 | 5.05573683 | 0.4789209 | 10.5565175 | 0 | 0 | 0 |
|  | PTX3 | NM_002852.3 | 4.13318809 | 0.48555751 | 8.5122525 | 1.11E-16 | 1.64E-16 | 8.55E-14 |
|  | PXDN | NM_012293.1 | 6.068366 | 0.44088102 | 13.7641805 | 0 | 0 | 0 |
|  | PYCARD | NM_013258.3 | 4.69421439 | 0.37362512 | 12.5639691 | 0 | 0 | 0 |
|  | QKI | NM_006775.2 | 6.12127646 | 0.40426396 | 15.1417812 | 0 | 0 | 0 |
|  | RAB25 | NM_020387.2 | 4.24102973 | 0.41813483 | 10.1427324 | 0 | 0 | 0 |
|  | RAC1 | NM_198829.1 | 6.26577439 | 0.3591189 | 17.447632 | 0 | 0 | 0 |
|  | RAC2 | NM_002872.3 | 5.43201285 | 0.50927138 | 10.6662441 | 0 | 0 | 0 |
|  | RAF1 | NM_002880.3 | 4.72100578 | 0.36398372 | 12.9703763 | 0 | 0 | 0 |
|  | RAMP1 | NM_005855.2 | 5.13471386 | 0.48504173 | 10.586128 | 0 | 0 | 0 |
|  | RAMP2 | NM_005854.2 | 4.35313878 | 0.49324476 | 8.82551458 | 0 | 0 | 0 |
|  | RB1 | NM_000321.1 | 5.20464012 | 0.42572129 | 12.2254635 | 0 | 0 | 0 |
|  | RBL1 | NM_183404.1 | 2.57994011 | 0.49458855 | 5.21633615 | 6.78E-07 | 7.42E-07 | 0.00052185 |
|  | RBL2 | NM_005611.3 | 5.39943189 | 0.34248832 | 15.7653023 | 0 | 0 | 0 |
|  | RBM47 | NM_019027.3 | 3.66593567 | 0.47791247 | 7.67072619 | 5.46E-14 | 7.55E-14 | 4.21E-11 |
|  | RBPJ | NM_015874.3 | 5.77072526 | 0.39391752 | 14.6495776 | 0 | 0 | 0 |
|  | RBX1 | NM_014248.2 | 5.98244192 | 0.31015879 | 19.28832 | 0 | 0 | 0 |
|  | RHOA | NM_001664.2 | 6.82351224 | 0.3210552 | 21.2533928 | 0 | 0 | 0 |
|  | RNH1 | NM_203384.1 | 5.64636762 | 0.31825193 | 17.7418174 | 0 | 0 | 0 |
|  | ROBO4 | NM_019055.5 | 3.68341185 | 0.55095781 | 6.68546993 | 8.60E-11 | 1.06E-10 | 6.62E-08 |
|  | ROCK1 | NM_005406.1 | 5.62074417 | 0.34407025 | 16.3360364 | 0 | 0 | 0 |
|  | ROCK2 | NM_004850.3 | 5.42476191 | 0.35090642 | 15.4592837 | 0 | 0 | 0 |
|  | RORA | NM_134261.2 | 5.28429458 | 0.40942647 | 12.9065779 | 0 | 0 | 0 |
|  | RPS27A | NM_002954.5 | 8.20518002 | 0.38838647 | 21.1263281 | 0 | 0 | 0 |
|  | RPS6KB1 | NM_003161.2 | 4.5346874 | 0.35168244 | 12.8942675 | 0 | 0 | 0 |
|  | RPS6KB2 | NM_003952.2 | 3.94441293 | 0.35449133 | 11.1269659 | 0 | 0 | 0 |
|  | RRAS | NM_006270.3 | 4.89193449 | 0.33549638 | 14.5811843 | 0 | 0 | 0 |
|  | RTN4 | NM_007008.2 | 6.31612205 | 0.34491236 | 18.312252 | 0 | 0 | 0 |
|  | RUNX1 | NM_001754.4 | 5.01114776 | 0.33703391 | 14.8683786 | 0 | 0 | 0 |
|  | RUNX1T1 | NM_004349.2 | 4.35192358 | 0.48184026 | 9.0318804 | 0 | 0 | 0 |
|  | S100A11P | NM_020672.1 | 4.13381132 | 0.51018401 | 8.10258892 | 2.11E-15 | 3.04E-15 | 1.62E-12 |
|  | S100A7 | NM_002963.2 | 4.24949998 | 0.5959796 | 7.13027757 | 4.95E-12 | 6.39E-12 | 3.81E-09 |
|  | S1PR1 | NM_001400.4 | 4.12475388 | 0.46685481 | 8.83519639 | 0 | 0 | 0 |
|  | SACS | NM_014363.4 | 3.92992265 | 0.44443982 | 8.84241794 | 0 | 0 | 0 |
|  | SAMSN1 | NM_022136.3 | 4.98097842 | 0.53292403 | 9.3465074 | 0 | 0 | 0 |
|  | SAP130 | NM_024545.3 | 4.03533768 | 0.38259122 | 10.547387 | 0 | 0 | 0 |
|  | SCG2 | NM_003469.3 | 3.47034775 | 0.59793083 | 5.80392848 | 2.98E-08 | 3.40E-08 | 2.30E-05 |
|  | SCNN1A | NM_001038.4 | 4.37135461 | 0.47809428 | 9.14328989 | 0 | 0 | 0 |
|  | SDC4 | NM_002999.2 | 6.22694937 | 0.43884322 | 14.1894623 | 0 | 0 | 0 |
|  | SERINC5 | NM_001174071.1 | 4.54379464 | 0.3717408 | 12.2230184 | 0 | 0 | 0 |
| *(cont. from previous page)*  **CRLM stroma *versus* paired primary CRC stroma** | SERPINA1 | NM_000295.4 | 9.36949938 | 0.67939699 | 13.790905 | 0 | 0 | 0 |
|  | SERPINE1 | NM_001165413.1 | 8.26585065 | 0.53473515 | 15.4578404 | 0 | 0 | 0 |
|  | SERPINF1 | NM_002615.4 | 6.28707609 | 0.48403536 | 12.9888776 | 0 | 0 | 0 |
|  | SERPING1 | NM_000062.2 | 6.65441172 | 0.47715865 | 13.9459102 | 0 | 0 | 0 |
|  | SERPINH1 | NM_001235.2 | 7.12093746 | 0.3284548 | 21.680114 | 0 | 0 | 0 |
|  | SET | NM_001122821.1 | 5.68160575 | 0.3804692 | 14.9331557 | 0 | 0 | 0 |
|  | SETD2 | NM_014159.6 | 5.14197491 | 0.35713873 | 14.3976963 | 0 | 0 | 0 |
|  | SF3A3 | NM_006802.2 | 5.37040898 | 0.3034499 | 17.6978439 | 0 | 0 | 0 |
|  | SFRP1 | NM_003012.3 | 5.36208199 | 0.5300739 | 10.1157254 | 0 | 0 | 0 |
|  | SH2B3 | NM_005475.2 | 5.14968938 | 0.43041508 | 11.9644725 | 0 | 0 | 0 |
|  | SH2D3A | NM_005490.2 | 4.45333569 | 0.44662928 | 9.97098923 | 0 | 0 | 0 |
|  | SH3YL1 | NM_001159597.1 | 3.21276889 | 0.40318537 | 7.96846588 | 6.55E-15 | 9.31E-15 | 5.04E-12 |
|  | SHB | NM_003028.2 | 4.33283613 | 0.43294746 | 10.0077644 | 0 | 0 | 0 |
|  | SIRT1 | NM_012238.4 | 4.02852136 | 0.38428597 | 10.4831341 | 0 | 0 | 0 |
|  | SKP1 | NM_170679.2 | 7.03060468 | 0.31823426 | 22.0925448 | 0 | 0 | 0 |
|  | SLC12A6 | NM_001042494.1 | 4.04792666 | 0.38259605 | 10.5801582 | 0 | 0 | 0 |
|  | SLC2A1 | NM_006516.2 | 4.28536962 | 0.47646998 | 8.99399706 | 0 | 0 | 0 |
|  | SLC35A3 | NM_012243.1 | 3.81886343 | 0.40735831 | 9.37470367 | 0 | 0 | 0 |
|  | SLC37A1 | NM_018964.3 | 4.0087033 | 0.49314683 | 8.12882308 | 2.00E-15 | 2.89E-15 | 1.54E-12 |
|  | SLIT2 | NM_004787.1 | 4.30072519 | 0.62067975 | 6.92905673 | 1.61E-11 | 2.04E-11 | 1.24E-08 |
|  | SLPI | NM_003064.2 | 3.90873051 | 0.4851641 | 8.05651224 | 3.77E-15 | 5.41E-15 | 2.91E-12 |
|  | SMAD1 | NM_005900.2 | 4.50557793 | 0.34770913 | 12.9578936 | 0 | 0 | 0 |
|  | SMAD2 | NM_005901.5 | 4.85308304 | 0.3734969 | 12.9936368 | 0 | 0 | 0 |
|  | SMAD3 | NM_005902.3 | 5.2390671 | 0.28039943 | 18.6843004 | 0 | 0 | 0 |
|  | SMAD4 | NM_005359.3 | 5.5316468 | 0.36503227 | 15.153857 | 0 | 0 | 0 |
|  | SMAD5 | NM_005903.5 | 4.74130512 | 0.3972423 | 11.9355496 | 0 | 0 | 0 |
|  | SMAD9 | NM_001127217.2 | 3.42643704 | 0.50377218 | 6.80156065 | 4.18E-11 | 5.22E-11 | 3.22E-08 |
|  | SMC3 | NM_005445.3 | 4.66039985 | 0.37747385 | 12.3462852 | 0 | 0 | 0 |
|  | SMOC1 | NM_001034852.1 | 3.9731502 | 0.57790205 | 6.87512738 | 2.15E-11 | 2.71E-11 | 1.66E-08 |
|  | SMURF2 | NM_022739.3 | 4.98629515 | 0.35744251 | 13.949922 | 0 | 0 | 0 |
|  | SNAI1 | NM_005985.2 | 5.31667611 | 0.36682705 | 14.493686 | 0 | 0 | 0 |
|  | SNAI2 | NM_003068.3 | 4.28406488 | 0.47366578 | 9.04448892 | 0 | 0 | 0 |
|  | SNAI3 | NM_178310.1 | 4.19551891 | 0.50461532 | 8.31429152 | 3.33E-16 | 4.88E-16 | 2.56E-13 |
|  | SNRPF | NM_003095.2 | 4.86375625 | 0.4321455 | 11.2549043 | 0 | 0 | 0 |
|  | SOD1 | NM_000454.4 | 4.59528579 | 0.43279252 | 10.617757 | 0 | 0 | 0 |
|  | SORD | NM_003104.4 | 5.15423619 | 0.59862897 | 8.61006806 | 1.11E-16 | 1.64E-16 | 8.55E-14 |
|  | SOX17 | NM_022454.3 | 4.26328308 | 0.52194081 | 8.16813521 | 1.78E-15 | 2.58E-15 | 1.37E-12 |
|  | SOX2 | NM_003106.2 | 2.84865129 | 0.64016599 | 4.44986351 | 3.37E-05 | 3.58E-05 | 0.02592918 |
|  | SOX9 | NM_000346.2 | 4.95880733 | 0.5170674 | 9.59025329 | 0 | 0 | 0 |
|  | SP1 | NM_003109.1 | 4.98305825 | 0.34585846 | 14.4077962 | 0 | 0 | 0 |
|  | SPARC | NM_003118.2 | 9.48384255 | 0.40374536 | 23.4896632 | 0 | 0 | 0 |
| *(cont. from previous page)*  **CRLM stroma *versus* paired primary CRC stroma** | SPARCL1 | NM_004684.4 | 4.32341353 | 0.4444906 | 9.72667034 | 0 | 0 | 0 |
|  | SPDEF | NM_012391.1 | 4.20256762 | 0.62906889 | 6.68061585 | 1.12E-10 | 1.38E-10 | 8.63E-08 |
|  | SPHK2 | NM_020126.3 | 4.49880257 | 0.37860596 | 11.8825455 | 0 | 0 | 0 |
|  | SPINK5 | NM_006846.3 | 3.35315598 | 0.61234596 | 5.47591752 | 1.58E-07 | 1.76E-07 | 0.00012168 |
|  | SPINT1 | NM_001032367.1 | 4.56779986 | 0.37454071 | 12.1957365 | 0 | 0 | 0 |
|  | SPOCK3 | NM_001204355.1 | 3.70021641 | 0.62263454 | 5.94283834 | 1.04E-08 | 1.19E-08 | 7.98E-06 |
|  | SPP1 | NM_000582.2 | 7.80858617 | 0.70846243 | 11.0218776 | 0 | 0 | 0 |
|  | SRC | NM_005417.3 | 4.46165921 | 0.33395491 | 13.3600649 | 0 | 0 | 0 |
|  | SRF | NM_003131.2 | 5.29972064 | 0.32758986 | 16.1779143 | 0 | 0 | 0 |
|  | SRGN | NR_036430.1 | 7.25204873 | 0.47762435 | 15.1835826 | 0 | 0 | 0 |
|  | SRPK2 | NM_182692.1 | 4.51092345 | 0.38516775 | 11.7115813 | 0 | 0 | 0 |
|  | SRPX2 | NM_014467.2 | 5.02625748 | 0.5368056 | 9.36327311 | 0 | 0 | 0 |
|  | SSTR2 | NM_001050.2 | 3.58662918 | 0.60918164 | 5.88761868 | 1.41E-08 | 1.62E-08 | 1.09E-05 |
|  | ST14 | NM_021978.3 | 5.14385467 | 0.40604252 | 12.6682661 | 0 | 0 | 0 |
|  | STAB1 | NM_015136.2 | 4.86648058 | 0.42295198 | 11.5059883 | 0 | 0 | 0 |
|  | STAB2 | NM_017564.9 | 3.91585338 | 0.55366166 | 7.0726468 | 5.51E-12 | 7.09E-12 | 4.24E-09 |
|  | STAT1 | NM_139266.1 | 6.85624778 | 0.46575012 | 14.7208718 | 0 | 0 | 0 |
|  | STAT3 | NM_139276.2 | 7.00732678 | 0.2953826 | 23.7228832 | 0 | 0 | 0 |
|  | SULF1 | NM_001128204.1 | 7.57430543 | 0.41736658 | 18.1478485 | 0 | 0 | 0 |
|  | SV2B | NM_001167580.1 | 3.3909289 | 0.63109603 | 5.37307908 | 2.87E-07 | 3.16E-07 | 0.00022087 |
|  | SYK | NM_003177.3 | 4.75502824 | 0.40230221 | 11.8195431 | 0 | 0 | 0 |
|  | SYNE1 | NM_015293.1 | 3.91519819 | 0.54369996 | 7.20102717 | 2.20E-12 | 2.89E-12 | 1.70E-09 |
|  | TACSTD2 | NM_002353.2 | 4.87222046 | 0.71857724 | 6.78037127 | 3.91E-11 | 4.90E-11 | 3.01E-08 |
|  | TAL1 | NM_003189.2 | 4.15315564 | 0.60598994 | 6.85350592 | 2.99E-11 | 3.76E-11 | 2.30E-08 |
|  | TBX1 | NM_080646.1 | 3.50602386 | 0.62266654 | 5.63066042 | 6.57E-08 | 7.41E-08 | 5.06E-05 |
|  | TBX4 | NM_018488.2 | 3.76170067 | 0.64324678 | 5.84798993 | 2.01E-08 | 2.30E-08 | 1.55E-05 |
|  | TBXA2R | NM_001060.3 | 4.96785664 | 0.49190056 | 10.0993107 | 0 | 0 | 0 |
|  | TCEB1 | NM_001204857.1 | 5.7417774 | 0.38789078 | 14.8025622 | 0 | 0 | 0 |
|  | TCEB2 | NM_007108.2 | 3.33498755 | 0.44615907 | 7.47488466 | 2.57E-13 | 3.44E-13 | 1.98E-10 |
|  | TCF20 | NM_005650.1 | 4.83436615 | 0.41047073 | 11.7776147 | 0 | 0 | 0 |
|  | TCF3 | NM_003200.3 | 3.83112687 | 0.34066251 | 11.2461066 | 0 | 0 | 0 |
|  | TCF7L2 | NM_003199.1 | 5.97209177 | 0.35913327 | 16.6291797 | 0 | 0 | 0 |
|  | TDGF1 | NM_003212.2 | 4.29802883 | 0.69700189 | 6.16645218 | 2.87E-09 | 3.37E-09 | 2.21E-06 |
|  | TEK | NM_000459.3 | 3.21033469 | 0.63845776 | 5.02826478 | 1.85E-06 | 2.01E-06 | 0.00142369 |
|  | TGFB1 | NM_000660.3 | 5.15108903 | 0.41945745 | 12.2803613 | 0 | 0 | 0 |
|  | TGFB2 | NM_003238.2 | 3.86025079 | 0.57095981 | 6.76098517 | 5.72E-11 | 7.11E-11 | 4.41E-08 |
|  | TGFBI | NM_000358.2 | 7.87515293 | 0.54413196 | 14.4728734 | 0 | 0 | 0 |
|  | TGFBR2 | NM_001024847.1 | 5.74032264 | 0.37799266 | 15.1863338 | 0 | 0 | 0 |
|  | THBS1 | NM_003246.2 | 7.2516316 | 0.51599006 | 14.0538204 | 0 | 0 | 0 |
|  | THBS2 | NM_003247.2 | 8.5538436 | 0.49034325 | 17.4446037 | 0 | 0 | 0 |
|  | THY1 | NM_006288.2 | 5.77192873 | 0.35944484 | 16.0578987 | 0 | 0 | 0 |
| *(cont. from previous page)*  **CRLM stroma *versus* paired primary CRC stroma** | TIE1 | NM_005424.2 | 4.42203469 | 0.47531396 | 9.30339745 | 0 | 0 | 0 |
|  | TIMP1 | NM_003254.2 | 9.92382236 | 0.49410302 | 20.0845207 | 0 | 0 | 0 |
|  | TIMP2 | NM_003255.4 | 7.41712492 | 0.38197763 | 19.4176944 | 0 | 0 | 0 |
|  | TIMP4 | NM_003256.2 | 3.61259429 | 0.49931596 | 7.23508672 | 1.59E-12 | 2.08E-12 | 1.22E-09 |
|  | TJP2 | NM_004817.2 | 4.56589151 | 0.36455082 | 12.524705 | 0 | 0 | 0 |
|  | TJP3 | NM_014428.1 | 4.4926235 | 0.47160812 | 9.52617921 | 0 | 0 | 0 |
|  | TLK2 | NM_006852.2 | 5.48469574 | 0.3956926 | 13.8610017 | 0 | 0 | 0 |
|  | TLR4 | NR_024168.1 | 5.44279956 | 0.52933809 | 10.2822746 | 0 | 0 | 0 |
|  | TMC6 | NM_001127198.1 | 4.24229024 | 0.40577193 | 10.4548637 | 0 | 0 | 0 |
|  | TMEM100 | NM_018286.2 | 3.95749638 | 0.71549988 | 5.53109298 | 1.13E-07 | 1.27E-07 | 8.74E-05 |
|  | TMEM30B | NM_001017970.2 | 4.37684272 | 0.45466331 | 9.62655803 | 0 | 0 | 0 |
|  | TMPRSS4 | NM_019894.3 | 3.04518942 | 0.56477436 | 5.39186912 | 2.63E-07 | 2.91E-07 | 0.0002028 |
|  | TMPRSS6 | NM_153609.2 | 3.50390017 | 0.61047708 | 5.73960967 | 3.78E-08 | 4.29E-08 | 2.91E-05 |
|  | TMUB2 | NM_024107.2 | 4.50186673 | 0.38521893 | 11.6865147 | 0 | 0 | 0 |
|  | TNC | NM_002160.3 | 3.99970847 | 0.49890223 | 8.01701865 | 4.33E-15 | 6.19E-15 | 3.33E-12 |
|  | TNF | NM_000594.2 | 3.93183885 | 0.56341954 | 6.97852773 | 1.07E-11 | 1.37E-11 | 8.26E-09 |
|  | TNFRSF12A | NM_016639.1 | 4.26745774 | 0.34135964 | 12.5013541 | 0 | 0 | 0 |
|  | TNFRSF1A | NM_001065.2 | 5.70451149 | 0.3050894 | 18.6978357 | 0 | 0 | 0 |
|  | TNFSF10 | NM_003810.2 | 5.78025393 | 0.34483401 | 16.7624241 | 0 | 0 | 0 |
|  | TNFSF12 | NM_003809.2 | 5.19533411 | 0.41415811 | 12.5443254 | 0 | 0 | 0 |
|  | TNFSF13 | NM_003808.3 | 5.34289123 | 0.31945596 | 16.7249699 | 0 | 0 | 0 |
|  | TNN | NM_022093.1 | 4.15468102 | 0.53684882 | 7.73901495 | 3.02E-14 | 4.19E-14 | 2.33E-11 |
|  | TNS1 | NM_022648.4 | 6.40799296 | 0.39776995 | 16.1097966 | 0 | 0 | 0 |
|  | TNXB | NM_032470.3 | 4.4421739 | 0.45473026 | 9.7688108 | 0 | 0 | 0 |
|  | TOM1L1 | NM_005486.2 | 4.12771432 | 0.47301469 | 8.72639776 | 0 | 0 | 0 |
|  | TP53 | NM_000546.2 | 5.03485711 | 0.5441601 | 9.25252909 | 0 | 0 | 0 |
|  | TPM2 | NM_003289.3 | 7.65702378 | 0.37308786 | 20.5233796 | 0 | 0 | 0 |
|  | TPS1 | NM_024164.5 | 5.88011069 | 0.57175986 | 10.2842314 | 0 | 0 | 0 |
|  | TPSD1 | NM_012217.2 | 4.12939818 | 0.57820323 | 7.14177644 | 3.33E-12 | 4.31E-12 | 2.56E-09 |
|  | TRIM39 | NM_021253.3 | 4.29541588 | 0.42470143 | 10.113966 | 0 | 0 | 0 |
|  | TWIST1 | NM_000474.3 | 3.58623513 | 0.53056444 | 6.75928288 | 5.53E-11 | 6.88E-11 | 4.26E-08 |
|  | TWIST2 | NM_057179.2 | 3.56866558 | 0.50558799 | 7.05844606 | 5.62E-12 | 7.22E-12 | 4.33E-09 |
|  | TXNIP | NM_006472.1 | 8.24094648 | 0.45778266 | 18.0018756 | 0 | 0 | 0 |
|  | TYMP | NM_001953.3 | 6.49414577 | 0.44462999 | 14.6057303 | 0 | 0 | 0 |
|  | UBA52 | NM_003333.3 | 6.9311464 | 0.37368569 | 18.5480646 | 0 | 0 | 0 |
|  | USP39 | NM_001256725.1 | 4.04509454 | 0.4310432 | 9.38442961 | 0 | 0 | 0 |
|  | UTS2 | NM_006786.3 | 3.3835673 | 0.60826994 | 5.5626081 | 1.04E-07 | 1.16E-07 | 8.01E-05 |
|  | VAMP8 | NM_003761.3 | 4.96427848 | 0.30539638 | 16.2551977 | 0 | 0 | 0 |
|  | VASH1 | NM_014909.4 | 4.88554043 | 0.47098646 | 10.3729955 | 0 | 0 | 0 |
|  | VAV2 | NM_003371.3 | 4.18929765 | 0.44035443 | 9.51346765 | 0 | 0 | 0 |
|  | VCAM1 | NM_001078.3 | 5.30559964 | 0.45454613 | 11.6723018 | 0 | 0 | 0 |
| *(cont. from previous page)*  **CRLM stroma *versus* paired primary CRC stroma** | VCAN | NM_004385.3 | 6.98469548 | 0.56656421 | 12.3281621 | 0 | 0 | 0 |
|  | VEGFA | NM_001025366.1 | 6.19054434 | 0.41106434 | 15.0597942 | 0 | 0 | 0 |
|  | VEGFB | NM_003377.3 | 5.43347892 | 0.36715312 | 14.7989454 | 0 | 0 | 0 |
|  | VEGFC | NM_005429.2 | 3.81639296 | 0.5762935 | 6.62230787 | 1.32E-10 | 1.61E-10 | 1.01E-07 |
|  | VEZF1 | NM_007146.2 | 4.56029503 | 0.41728828 | 10.9284044 | 0 | 0 | 0 |
|  | VHL | NM_000551.2 | 5.51891302 | 0.31702872 | 17.4082431 | 0 | 0 | 0 |
|  | VIM | NM_003380.2 | 9.01470017 | 0.45912131 | 19.6346803 | 0 | 0 | 0 |
|  | VIT | NM_053276.3 | 3.61576288 | 0.50457082 | 7.16601669 | 2.76E-12 | 3.60E-12 | 2.13E-09 |
|  | VPS13A | NM_033305.2 | 4.39021869 | 0.38937459 | 11.2750519 | 0 | 0 | 0 |
|  | VSIG4 | NM_001100431.1 | 6.06311851 | 0.47797038 | 12.6851344 | 0 | 0 | 0 |
|  | VWA1 | NM_199121.2 | 3.40561941 | 0.50716081 | 6.71506812 | 8.24E-11 | 1.02E-10 | 6.35E-08 |
|  | VWA2 | NM_001272046.1 | 4.28866551 | 0.68634755 | 6.2485333 | 1.38E-09 | 1.64E-09 | 1.06E-06 |
|  | WARS | NM_004184.3 | 5.4238504 | 0.38325089 | 14.1522187 | 0 | 0 | 0 |
|  | WIPF1 | NM_001077269.1 | 5.4468967 | 0.45794435 | 11.8942329 | 0 | 0 | 0 |
|  | WNT5A | NM_003392.3 | 4.25738801 | 0.46579548 | 9.14003712 | 0 | 0 | 0 |
|  | WNT5B | NM_032642.2 | 4.1023316 | 0.62674576 | 6.54544769 | 1.97E-10 | 2.39E-10 | 1.52E-07 |
|  | WWTR1 | NM_001168278.1 | 5.56438541 | 0.37516378 | 14.8318835 | 0 | 0 | 0 |
|  | ZC3H12A | NM_025079.2 | 4.69195608 | 0.40673892 | 11.5355474 | 0 | 0 | 0 |
|  | ZC3H14 | NM_001160103.1 | 4.82164494 | 0.30193706 | 15.9690397 | 0 | 0 | 0 |
|  | ZCCHC24 | XM_005269604.1 | 4.32827195 | 0.43789172 | 9.88434309 | 0 | 0 | 0 |
|  | ZEB1 | NM_001128128.1 | 5.1631343 | 0.44146006 | 11.6955866 | 0 | 0 | 0 |
|  | ZEB2 | NM_014795.3 | 5.71769197 | 0.45432998 | 12.5848882 | 0 | 0 | 0 |
|  | ZFPM2 | NM_012082.3 | 3.36640617 | 0.61655873 | 5.45999267 | 1.79E-07 | 1.99E-07 | 0.00013804 |
|  | ZFYVE16 | NM_001105251.2 | 4.62578356 | 0.40208765 | 11.5044159 | 0 | 0 | 0 |
|  | ZFYVE9 | NM_004799.2 | 4.0079651 | 0.38812295 | 10.3265348 | 0 | 0 | 0 |
|  | ZKSCAN5 | NM_014569.3 | 2.46337335 | 0.45759287 | 5.3833299 | 3.08E-07 | 3.40E-07 | 0.00023741 |
|  | ZNF143 | NM_003442.5 | 2.81747053 | 0.35268638 | 7.9886004 | 6.22E-15 | 8.85E-15 | 4.79E-12 |
|  | ZNF346 | NM_012279.2 | 4.05286801 | 0.38539492 | 10.5161429 | 0 | 0 | 0 |
|  |  |  |  |  |  |  |  |  |
| **CRLM tumour *versus* paired primary CRC tumour** | ALB | NM_000477.5 | 4.24706312 | 0.64585024 | 6.57592563 | 1.63E-10 | 1.26E-07 | 1.26E-07 |
|  | COL4A2 | NM_001846.2 | 1.03726331 | 0.23750678 | 4.36729983 | 5.01E-05 | 0.01697716 | 0.03856664 |

**Supplementary Table 7 –** Full list of enriched pathways after DGE analysis for case primary CRC stroma versus control primary CRC stroma and CRLM stroma versus paired primary stroma.

| **Comparison** | **Pathway Enrichment** | **Pathway Name** | **p-values** | **Matches** |
| --- | --- | --- | --- | --- |
| **Case primary CRC stroma *versus* control primary CRC stroma** | LIPASYN-PWY | phospholipases | 0.03245283 | HS04103 |
|  |  |  |  |  |
| **CRLM stroma *versus* paired primary CRC stroma** | Signalling-Pathways | Signal transduction pathways | 3.84E-13 | MAP2K2 // "MAP2K1" // "MAPK3" // "MAPK1" // "RAF1" // "ZFYVE16" // "SMAD4" // "FSTL1" // "CHRDL1" // "SMAD9" // "SMAD5" // "SMAD1" // "SMURF2" |
|  | PWY66-11 | BMP Signalling Pathway | 8.46E-08 | ZFYVE16 // "SMAD4" // "FSTL1" // "CHRDL1" // "SMAD9" // "SMAD5" // "SMAD1" // "SMURF2" |
|  | GLYCOLYSIS-VARIANTS | Glycolysis | 1.34E-07 | GPI // "HK2" // "HK3" // "HKDC1" // "PKM2" // "ENO1" // "ENO2" // "ENO3" // "PGK1" // "ALDOA" |
|  | PWY66-400 | glycolysis | 1.34E-07 | GPI // "HK2" // "HK3" // "HKDC1" // "PKM2" // "ENO1" // "ENO2" // "ENO3" // "PGK1" // "ALDOA" |
|  | PWY66-14 | MAP kinase cascade | 1.92E-06 | MAP2K2 // "MAP2K1" // "MAPK3" // "MAPK1" // "RAF1" |
|  | PWY66-407 | superpathway of conversion of glucose to acetyl CoA and entry into the TCA cycle | 1.02E-04 | GPI // "HK2" // "HK3" // "HKDC1" // "PKM2" // "ENO1" // "ENO2" // "ENO3" // "PGK1" // "ALDOA" |
|  | Gluconeogenesis | Gluconeogenesis | 1.31E-04 | GPI // "ENO1" // "ENO2" // "ENO3" // "PGK1" // "ALDOA" // "FBP1" |
|  | PWY66-399 | gluconeogenesis | 1.31E-04 | GPI // "ENO1" // "ENO2" // "ENO3" // "PGK1" // "ALDOA" // "FBP1" |
|  | PWY-6352 | 3-phosphoinositide biosynthesis | 3.78E-04 | PIK3CG // "PIK3R6" // "PIK3R5" // "PIK3CA" // "PIK3R1" // "PIK3CD" // "PIK3R2" |
|  | CARBO-BIOSYNTHESIS | Sugars Biosynthesis | 4.61E-04 | HK2 // "HK3" // "HKDC1" // "GPI" // "ENO1" // "ENO2" // "ENO3" // "PGK1" // "ALDOA" // "FBP1" |
|  | PWY-6371 | superpathway of inositol phosphate compounds | 6.33E-04 | MTMR14 // "PLCG2" // "PLCG1" // "PIK3CG" // "PIK3R6" // "PIK3R5" // "PIK3CA" // "PIK3R1" // "PIK3CD" // "PIK3R2" // "PTEN" |
|  | Energy-Metabolism | Generation of Precursor Metabolites and Energy | 7.18E-04 | GPI // "HK2" // "HK3" // "HKDC1" // "PKM2" // "ENO1" // "ENO2" // "ENO3" // "PGK1" // "ALDOA" // "LDHA" |
|  | Cyclitols-Biosynthesis | Cyclitols Biosynthesis | 0.001300731 | PTEN // "MTMR14" // "PLCG2" // "PLCG1" // "PIK3CG" // "PIK3R6" // "PIK3R5" // "PIK3CA" // "PIK3R1" // "PIK3CD" // "PIK3R2" |
|  | SUGAR-DERIVS | Sugar Derivatives Biosynthesis | 0.001300731 | PTEN // "MTMR14" // "PLCG2" // "PLCG1" // "PIK3CG" // "PIK3R6" // "PIK3R5" // "PIK3CA" // "PIK3R1" // "PIK3CD" // "PIK3R2" |
|  | PWY66-393 | aspirin-triggered lipoxin biosynthesis | 0.002674571 | PTGS2 // "ALOX5" |
|  | PWY66-395 | aspirin triggered resolvin D biosynthesis | 0.002674571 | ALOX5 // "PTGS2" |
|  | PWY66-394 | aspirin triggered resolvin E biosynthesis | 0.002674571 | PTGS2 // "ALOX5" |
|  | PWY-5661-1 | GDP-glucose biosynthesis II | 0.004071428 | HK2 // "HK3" // "HKDC1" |
|  | PWY-5514 | UDP-<i>N</i>-acetyl-D-galactosamine biosynthesis II | 0.004539552 | GPI // "HK2" // "HK3" // "HKDC1" |
| *(cont. from previous page)*  **CRLM stroma *versus* paired primary CRC stroma** | UDP-Nac-Galactosamine Biosynthesis | UDP-<i>N</i>-acetyl-D-galactosamine Biosynthesis | 0.004539552 | GPI // "HK2" // "HK3" // "HKDC1" |
|  | SECONDARY METABOLITE BIOSYNTHESIS | Secondary Metabolites Biosynthesis | 0.00674924 | PTEN // "MTMR14" // "PLCG2" // "PLCG1" // "PIK3CG" // "PIK3R6" // "PIK3R5" // "PIK3CA" // "PIK3R1" // "PIK3CD" // "PIK3R2" |
|  | Phospholipid-Biosynthesis | Phospholipid Biosynthesis | 0.007164108 | PIK3CG // "PIK3R6" // "PIK3R5" // "PIK3CA" // "PIK3R1" // "PIK3CD" // "PIK3R2" // "MTMR14" // "PLCG2" // "PLCG1" // "PTEN" // "CDS1" |
|  | PWY66-374 | C20 prostanoid biosynthesis | 0.009059869 | PTGDS // "PTGIS" // "PTGS2" |
|  | GDP-Sugar-Biosynthesis | GDP-sugar Biosynthesis | 0.009583571 | HK2 // "HK3" // "HKDC1" // "GPI" |
|  | UDP-Sugar-Biosynthesis | UDP-sugar Biosynthesis | 0.020583024 | GPI // "HK2" // "HK3" // "HKDC1" |
|  | Metabolic-Regulators | Metabolic Regulators Biosynthesis | 0.020583024 | PFKFB1 // "PFKFB4" // "NOS3" // "CKMT1A" |
|  | LIPASYN-PWY | phospholipases | 0.021153692 | PLA2G2A // "PLA2G3" // "PLA2G10" // "PLA2G2D" // "PLCG2" // "PLCG1" |
|  | PWY66-423 | fructose 2,6-bisphosphate synthesis | 0.024136873 | PFKFB1 // "PFKFB4" |
|  | Trehalose-Degradation | Trehalose Degradation | 0.024136873 | HK2 // "HK3" |
|  | PWY0-1182 | trehalose degradation | 0.024136873 | HK2 // "HK3" |
|  | PWY-7899 | protein neddylation | 0.03499708 | RBX1 // "CUL1" |
|  | PWY-6367 | D-<i>myo</i>-inositol-5-phosphate metabolism | 0.081628844 | MTMR14 // "PLCG2" // "PLCG1" |
|  | PWY-7205 | CMP phosphorylation | 0.091789566 | NME4 // "NME1" |
|  | SUGAR-NUCLEOTIDES | Sugar Nucleotides Biosynthesis | 0.09616357 | HK2 // "HK3" // "HKDC1" // "GPI" |
