# Supplementary material for: Cancer‐associated stroma reveals prognostic biomarkers and novel insights into the tumour microenvironment of colorectal cancer and colorectal liver metastases
Source: Cancer Med. 2021 Dec 7;11(2):492–506. doi: 10.1002/cam4.4452 (PMC8729056; doi:10.1002/cam4.4452)
Supplement: Supplementary file 4 — Method S1 [file CAM4-11-492-s003.docx]

**Supplementary Methods**

- 1. *Immunohistochemisty (IHC) staining protocol*

| **Antigen** | **Manufacturer** | **Product number** | **Host** | **Clone** | **Optimised Dilution** | **Optimised Buffer** |
| --- | --- | --- | --- | --- | --- | --- |
| **FAP** | Sigma-Aldrich | HPA059739 | Rabbit | polyclonal | 1:400 | HIER pH6 |
| **pSMAD2 (pS255)** | Abcam | ab188334 | Rabbit | IgG monoclonal [EPR2856(N)] | 1:1600 | Tris/EDTA pH9 |
| **IGFBP7** | Sigma-Aldrich | HPA002196 | Rabbit | polyclonal | 1:200 | HIER pH6 |
| **POSTN** | Sigma-Aldrich | HPA012306 | Rabbit | polyclonal | 1:600 | HIER pH6 |
| **CALD1** | Sigma-Aldrich | HPA008066 | Rabbit | polyclonal | 1:600 | HIER pH6 |
| **TGF-b** | Sigma-Aldrich | T0438 | Mouse | IgG1 monoclonal | 1:50 | pH 9 |

**Supplementary Methods Table 1 – Optimised primary antibody conditions.**

Sigma-Aldrich (St Louis, Missouri, United States)

Abcam (Cambridge, United Kingdom)

4μm TMA sections were deparaffinized in xylene, rehydrated in graded ethanol concentrations, and quenched for 15min in 0.3% hydrogen peroxide. All IHC was performed manually. All slides underwent HIER (heat induced epitope retrieval) for 30min according to Supplementary Methods Table 1. 10% horse blocking serum was used for 40min at room temperature prior to primary antibody incubation overnight at 4^o^C. Secondary antibody incubation using the appropriate peroxidase (EnVision mouse/rabbit kit; DAKO, Glostrup, Denmark) was added for 30 minutes at room temperature, before DAB (3,3’Diaminobenzidine) chromogen, Mayer’s haematoxyline counter stain and Scott’s blueing solution.

- 1. *Full list of R packages used;*
- BaylorEdPsych (Beaujean 2012)
- broomExtra (Patil 2019)
- caret (Jed Wing et al. 2019)
- coin (Hothorn et al. 2006)
- colorspace (Zeileis et al. 2019)
- colourpicker (Attali 2017)
- corrplot (Wei 2017)
- cvms (Olsen and Zachariae 2019)
- dendextend (Galili 2015)
- DescTools (al. 2019)
- factoextra (Kassambara and Mundt 2017)
- FactoMineR (Lê, Josse, and Husson 2008)
- finalfit (Harrison, Drake, and Ots 2019)
- funModeling (Casas 2019)
- GGally (Schloerke et al. 2018)
- ggfortify (Tang, Horikoshi, and Li 2016)
- ggpubr (Kassambara 2019a)
- ggstatsplot (Patil 2018)
- GoodmanKruskal (Pearson 2016)
- groupdata2 (Olsen 2019)
- Hmisc (Harrell Jr, Charles Dupont, and others. 2019)
- knitr (Xie 2019)
- labelled (Larmarange 2019)
- lme4 (Bates et al. 2015)
- lsr (Navarro 2015)
- mctest (Imdad and Aslam 2018)
- mice (van Buuren and Groothuis-Oudshoorn 2011)
- missRanger (Mayer 2019)
- mitools (Lumley 2019)
- mixOmics (F et al. 2017)
- modelr (Wickham 2019)
- mvnmle (Gross and Douglas Bates 2018)
- naniar (Tierney et al. 2019)
- ordinal (Christensen 2019)
- psfmi (Heymans 2019)
- rmarkdown (Allaire et al. 2019)
- rms (Harrell Jr 2019)
- rstatix (Kassambara 2019b)
- summarytools (Comtois 2019)
- survival (Therneau T 2015)
- survivalAnalysis (Wiesweg 2019a)
- survminer (Kassambara, Kosinski, and Biecek 2019)
- tidymodels (Kuhn and Wickham 2019)
- tidytidbits (Wiesweg 2019b)
- tidyverse (Wickham et al. 2019)
- vcd (Meyer, Zeileis, and Hornik 2017)
- vcdExtra (Friendly 2017)

**References**

al., Andri Signorell et mult. 2019. *DescTools: Tools for Descriptive Statistics*. <https://cran.r-project.org/package=DescTools>.

Allaire, JJ, Yihui Xie, Jonathan McPherson, Javier Luraschi, Kevin Ushey, Aron Atkins, Hadley Wickham, Joe Cheng, Winston Chang, and Richard Iannone. 2019. *Rmarkdown: Dynamic Documents for R*. <https://github.com/rstudio/rmarkdown>.

Attali, Dean. 2017. *Colourpicker: A Colour Picker Tool for Shiny and for Selecting Colours in Plots*. <https://CRAN.R-project.org/package=colourpicker>.

Bates, Douglas, Martin Mächler, Ben Bolker, and Steve Walker. 2015. “Fitting Linear Mixed-Effects Models Using lme4.” *Journal of Statistical Software* 67 (1): 1–48. <https://doi.org/10.18637/jss.v067.i01>.

Beaujean, A. Alexander. 2012. *BaylorEdPsych: R Package for Baylor University Educational Psychology Quantitative Courses*. <https://CRAN.R-project.org/package=BaylorEdPsych>.

Casas, Pablo. 2019. *FunModeling: Exploratory Data Analysis and Data Preparation Tool-Box*. <https://CRAN.R-project.org/package=funModeling>.

Christensen, R. H. B. 2019. “Ordinal—Regression Models for Ordinal Data.”

Comtois, Dominic. 2019. *Summarytools: Tools to Quickly and Neatly Summarize Data*. <https://github.com/dcomtois/summarytools>.

F, Rohart, Gautier B, Singh A, and Le Cao K-A. 2017. “MixOmics: An R Package for ’Omics Feature Selection and Multiple Data Integration.” *PLoS Computational Biology* 13 (11): e1005752. <http://www.mixOmics.org>.

Friendly, Michael. 2017. *VcdExtra: ’Vcd’ Extensions and Additions*. <https://CRAN.R-project.org/package=vcdExtra>.

Galili, Tal. 2015. “Dendextend: An R Package for Visualizing, Adjusting, and Comparing Trees of Hierarchical Clustering.” *Bioinformatics*. <https://doi.org/10.1093/bioinformatics/btv428>.

Gross, Kevin, and with help from Douglas Bates. 2018. *Mvnmle: ML Estimation for Multivariate Normal Data with Missing Values*. <https://CRAN.R-project.org/package=mvnmle>.

Harrell Jr, Frank E. 2019. *Rms: Regression Modeling Strategies*. <https://CRAN.R-project.org/package=rms>.

Harrell Jr, Frank E, with contributions from Charles Dupont, and many others. 2019. *Hmisc: Harrell Miscellaneous*. <https://CRAN.R-project.org/package=Hmisc>.

Harrison, Ewen, Tom Drake, and Riinu Ots. 2019. *Finalfit: Quickly Create Elegant Regression Results Tables and Plots When Modelling*. <https://github.com/ewenharrison/finalfit>.

Heymans, Martijn. 2019. *Psfmi: Prediction Model Selection and Performance Evaluation in Multiple Imputed Datasets*. <https://CRAN.R-project.org/package=psfmi>.

Hothorn, Torsten, Kurt Hornik, Mark A. van de Wiel, and Achim Zeileis. 2006. “A Lego System for Conditional Inference.” *The American Statistician* 60 (3): 257–63. <https://doi.org/10.1198/000313006X118430>.

Imdad, M. U., and M. Aslam. 2018. *mctest: Multicollinearity Diagnostic Measures*. <https://CRAN.R-project.org/package=mctest>.

Jed Wing, Max Kuhn. Contributions from, Steve Weston, Andre Williams, Chris Keefer, Allan Engelhardt, Tony Cooper, Zachary Mayer, et al. 2019. *Caret: Classification and Regression Training*. <https://CRAN.R-project.org/package=caret>.

Kassambara, Alboukadel. 2019a. *Ggpubr: ’Ggplot2’ Based Publication Ready Plots*. <https://CRAN.R-project.org/package=ggpubr>.

Kassambara 2019b. *Rstatix: Pipe-Friendly Framework for Basic Statistical Tests*. <https://CRAN.R-project.org/package=rstatix>.

Kassambara, Alboukadel, Marcin Kosinski, and Przemyslaw Biecek. 2019. *Survminer: Drawing Survival Curves Using ’Ggplot2’*. <https://CRAN.R-project.org/package=survminer>.

Kassambara, Alboukadel, and Fabian Mundt. 2017. *Factoextra: Extract and Visualize the Results of Multivariate Data Analyses*. <https://CRAN.R-project.org/package=factoextra>.

Kuhn, Max, and Hadley Wickham. 2019. *Tidymodels: Easily Install and Load the ’Tidymodels’ Packages*. <https://CRAN.R-project.org/package=tidymodels>.

Larmarange, Joseph. 2019. *Labelled: Manipulating Labelled Data*. <https://CRAN.R-project.org/package=labelled>.

Lê, Sébastien, Julie Josse, and François Husson. 2008. “FactoMineR: A Package for Multivariate Analysis.” *Journal of Statistical Software* 25 (1): 1–18. <https://doi.org/10.18637/jss.v025.i01>.

Lumley, Thomas. 2019. *Mitools: Tools for Multiple Imputation of Missing Data*. <https://CRAN.R-project.org/package=mitools>.

Mayer, Michael. 2019. *MissRanger: Fast Imputation of Missing Values*. <https://CRAN.R-project.org/package=missRanger>.

Meyer, David, Achim Zeileis, and Kurt Hornik. 2017. *Vcd: Visualizing Categorical Data*.

Navarro, Daniel. 2015. *Learning Statistics with R: A Tutorial for Psychology Students and Other Beginners. (Version 0.5)*. Adelaide, Australia: University of Adelaide. <http://ua.edu.au/ccs/teaching/lsr>.

Olsen, Ludvig Renbo. 2019. *Groupdata2: Creating Groups from Data*. <https://github.com/ludvigolsen/groupdata2>.

Olsen, Ludvig Renbo, and Benjamin Hugh Zachariae. 2019. *Cvms: Cross-Validation for Model Selection*. <https://CRAN.R-project.org/package=cvms>.

Patil, Indrajeet. 2018. *Ggstatsplot: ’Ggplot2’ Based Plots with Statistical Details*. <https://doi.org/10.5281/zenodo.2074621>.

Patil, Indrajeet 2019. *BroomExtra: Enhancements for ’Broom’ Package Family*. <https://CRAN.R-project.org/package=broomExtra>.

Pearson, Ron. 2016. *GoodmanKruskal: Association Analysis for Categorical Variables*. <https://CRAN.R-project.org/package=GoodmanKruskal>.

Schloerke, Barret, Jason Crowley, Di Cook, Francois Briatte, Moritz Marbach, Edwin Thoen, Amos Elberg, and Joseph Larmarange. 2018. *GGally: Extension to ’Ggplot2’*. <https://CRAN.R-project.org/package=GGally>.

Tang, Yuan, Masaaki Horikoshi, and Wenxuan Li. 2016. “Ggfortify: Unified Interface to Visualize Statistical Result of Popular R Packages.” *The R Journal* 8 (2). <https://journal.r-project.org/>.

Therneau T (2015). _A Package for Survival Analysis in S_. version 2.38, <URL: <https://CRAN.R-project.org/package=survival>>.

Tierney, Nicholas, Di Cook, Miles McBain, and Colin Fay. 2019. *Naniar: Data Structures, Summaries, and Visualisations for Missing Data*. <https://CRAN.R-project.org/package=naniar>.

van Buuren, Stef, and Karin Groothuis-Oudshoorn. 2011. “mice: Multivariate Imputation by Chained Equations in R.” *Journal of Statistical Software* 45 (3): 1–67. <https://www.jstatsoft.org/v45/i03/>.

Wickham, Hadley. 2019. *Modelr: Modelling Functions That Work with the Pipe*. <https://CRAN.R-project.org/package=modelr>.

Wickham, Hadley, Mara Averick, Jennifer Bryan, Winston Chang, Lucy D’Agostino McGowan, Romain François, Garrett Grolemund, et al. 2019. “Welcome to the tidyverse.” *Journal of Open Source Software* 4 (43): 1686. <https://doi.org/10.21105/joss.01686>.

Wei, Taiyun and Simko, Viliam (2017). R package "corrplot": Visualization of a Correlation Matrix (Version 0.84). <https://github.com/taiyun/corrplot>

Wiesweg, Marcel. 2019a. *SurvivalAnalysis: High-Level Interface for Survival Analysis and Associated Plots*. <https://CRAN.R-project.org/package=survivalAnalysis>.

Wiesweg, Marcel 2019b. *Tidytidbits: A Collection of Tools and Helpers Extending the Tidyverse*. <https://CRAN.R-project.org/package=tidytidbits>.

Xie, Yihui. 2019. *Knitr: A General-Purpose Package for Dynamic Report Generation in R*. <https://yihui.name/knitr/>.

Zeileis, Achim, Jason C. Fisher, Kurt Hornik, Ross Ihaka, Claire D. McWhite, Paul Murrell, Reto Stauffer, and Claus O. Wilke. 2019. “colorspace: A Toolbox for Manipulating and Assessing Colors and Palettes.” ArXiv 1903.06490. arXiv.org E-Print Archive. <http://arxiv.org/abs/1903.06490>.
